# Dietary Diversity Indicators and Their Associations with Dietary Adequacy and Health Outcomes: A Systematic Scoping Review

**DOI:** 10.1093/advances/nmab009

**Published:** 2021-03-03

**Authors:** Eric O Verger, Agnes Le Port, Augustin Borderon, Gabriel Bourbon, Mourad Moursi, Mathilde Savy, François Mariotti, Yves Martin-Prevel

**Affiliations:** MoISA, Univ Montpellier, CIRAD, CIHEAM-IAMM, INRAE, Institut Agro, IRD, Montpellier, France; MoISA, Univ Montpellier, CIRAD, CIHEAM-IAMM, INRAE, Institut Agro, IRD, Montpellier, France; MoISA, Univ Montpellier, CIRAD, CIHEAM-IAMM, INRAE, Institut Agro, IRD, Montpellier, France; Independent Consultant, Paris, France; Intake—FHI Solutions, Washington, DC, USA; MoISA, Univ Montpellier, CIRAD, CIHEAM-IAMM, INRAE, Institut Agro, IRD, Montpellier, France; Université Paris-Saclay, AgroParisTech, INRAE, UMR PNCA, Paris, France; MoISA, Univ Montpellier, CIRAD, CIHEAM-IAMM, INRAE, Institut Agro, IRD, Montpellier, France

**Keywords:** dietary diversity, indicators, nutrition, diet quality, health outcomes, body weight, noncommunicable diseases, adolescent, adult

## Abstract

Dietary diversity has long been recognized as a key component of diet quality and many dietary diversity indicators (DDIs) have been developed. This systematic scoping review aimed to present a comprehensive inventory of DDIs and summarize evidence linking DDIs and dietary adequacy or health outcomes in adolescents and adults. Two search strategies were developed to identify peer-reviewed articles published in English up until June 2018 and were applied to Medline, Web of Science, and Scopus. A 2-stage screening process was used to select the studies to be reviewed. Four types of DDIs were identified among 161 articles, the majority of them belonging to the food group–based indicator type (*n =* 106 articles). Fifty studies indicated that DDIs were proxies of nutrient adequacy, but there was a lack of evidence about their relation with nutrients to limit. Associations between DDIs and health outcomes were largely inconsistent among 137 studies, especially when the outcomes studied were body weight (*n =* 60) and noncommunicable diseases (*n =* 41). We conclude that the ability of DDIs to reflect diet quality was found to be principally limited to micronutrient adequacy and that DDIs do not readily relate to health outcomes. These findings have implications for studies in low- and lower-middle-income economies where DDIs are often used to assess dietary patterns and overall diet quality.

## Introduction

Food diversity has long been recognized as a key element of high-quality diets, based on the principle that no single food can provide the right amount of nutrients necessary to maintain optimal health. This theory has been historically documented on many occasions, such as the high prevalence of beriberi, as well as night blindness and scurvy, among the Imperial Japanese Army during the first half of the 20th century, due to rice-based rations that included little or no other foods (1). Eating a variety of foods has been a longstanding recommendation of US dietary guidelines since 1980, initially phrased as “select foods each day from each of several major groups,” then as “choose a variety of nutrient-dense foods across and within all food groups in recommended amounts” (2, 3). Many other national and international food-based dietary guidelines have included dietary diversity as a key feature, albeit with slightly different definitions depending on the country or countries and cultural dietary patterns (4, 5). One result of these historical evolutions and national differences was a lack of consensus about what dietary diversity represents and how to measure and operationalize it (6).

Dietary diversity indicators (DDIs) have been identified as promising measurement tools, particularly for use in developing countries, because of their simplicity of implementation and their potential to be used at a large scale compared with other food-consumption indicators involving the collection of complex quantitative information (6). Thereafter, significant progress has been made in developing and validating simple DDIs with consistent and relevant meaning across different contexts and over time. As a result, many DDIs have been developed and used in the literature, either as proxies of nutritional adequacy of the diet or as recommendations to maintain optimal health (6–10). The most recent example is the Minimum Dietary Diversity for Women of Reproductive Age, which is calculated based on a single 24-h recall (11) and has been shown to be effective in predicting adequacy for 11 micronutrients in 9 large datasets from different countries (12).

While several comprehensive or systematic reviews have been carried out, the most recent ones focusing on obesity and body adiposity (8–10), there is a lack of comprehensive information about how dietary diversity is conceptualized and measured across contexts, and whether the proposed indicators are associated with dietary adequacy and health outcomes. Scoping reviews aim at identifying, mapping, and synthesizing the available evidence in a given field of interest (13) and are particularly relevant for the following purposes: *1*) clarify key concepts and definitions, *2*) identify key characteristics or factors related to a concept, and *3*) highlight knowledge gaps (14). The objectives of the present systematic scoping review are therefore as follows: *1*) to present a comprehensive inventory of DDIs developed for adolescents and adults, *2*) to summarize the evidence of the associations between DDIs and measures of dietary adequacy, and *3*) to summarize the evidence of the associations between DDIs and health outcomes.

## Methods

### Search strategies

Based on the Preferred Reporting Items for Systematic Reviews and Meta-Analyses Extension for Scoping Reviews (PRISMA-ScR) guidelines (15), 2 structured search strategies were developed to include title-abstract-keywords in 2 different search strings. The first search strategy focused on articles studying dietary diversity in association with measures of the dietary adequacy and included the title-abstract-keywords “diet diversity, diet variety, food diversity, food variety, nutrient intake, nutrient adequacy, diet quality,” including relevant variations in keywords. The second search strategy focused on articles studying dietary diversity in association with health outcomes and included the title-abstract-keywords “diet diversity, diet variety, food diversity, food variety, health outcome, risk factor, life expectancy, mortality, morbidity, comorbidity, cancer, disease, overweight, obesity, adiposity, anthropometric status, diabetes, blood glucose, cardiometabolic, metabolic syndrome, hypertension, blood pressure, dyslipidemia, anemia, low birth weight, incidence, vascular,” including relevant variations in keywords. The searches were limited to peer-reviewed articles published in English up until June 2018 and were run using 3 databases: Medline, Web of Science, and Scopus. Additionally, we examined reference lists in papers from the studies included to identify other relevant studies.

### Data extraction

All studies identified as suitable were extracted using Zotero (version 4.0.28.7). We conducted a 2-stage screening process to select the studies to be fully reviewed. During the first stage, titles and abstracts were examined by 2 authors (EOV and AB) and irrelevant studies were excluded from further review. At the second stage, a full-text screen was performed by 2 authors (EOV and ALP) and evaluated using the Population, Interventions, Comparators, Outcomes, and Study design (PICOS) criteria for inclusion and exclusion (16). All disagreements regarding eligibility were resolved by discussion.

### Inclusion and exclusion criteria

The PICOS criteria for inclusion and exclusion for title, abstract, and full-text screening are described in **Table 1**. Because our intent was to present a comprehensive inventory of DDIs developed for adolescents and adults, we did not exclude articles based on their methodological quality. Furthermore, our scoping review was not meant to assess the validity or quality of the studies, nor of the indicators they used.

**TABLE 1:** PICOS criteria for inclusion and exclusion of studies^1^

Parameter	Description
Population	Inclusion criteria: adults and adolescents (age ≥10 y)
	Exclusion criteria: children (age <10 y)
Interventions	Inclusion criteria: any study measuring participants' dietary variety or dietary diversity using an indicator based on the number of foods or food groups^2^ consumed over a reference period, regardless of the mathematical operation required to compute this information
Comparators	Inclusion criteria: participants with higher dietary diversity compared with participants with lower dietary diversity
Outcomes	Inclusion criteria:
	*1*) Any measure of dietary adequacy, including nutrient intake, nutrient adequacy index, and food-based diet quality index
	*2*) Any health outcome, including (but not restricted to) body weight and body composition, noncommunicable diseases and intermediate biomarkers of health, biomarkers of nutritional status, mental health and cognitive functions, and mortality
Study design	Inclusion criteria: all study designs
	Exclusion criteria: reviews, expert opinion, comments, letters to the editor, studies on animals, conference reports

1 PICOS, Population, Interventions, Comparators, Outcomes and Study design criteria.

2 A food group is a collection of foods that share similar nutritional properties or biological classifications.

### Synthesis of the results

Due to the wide heterogeneity among DDIs, measures of dietary adequacy, health outcomes, study designs, and statistical models, we used a qualitative and descriptive approach to review the available evidence regarding whether dietary diversity is associated with dietary adequacy and various health outcomes. The association between DDIs and any measure of dietary adequacy was described as “positive” (the higher the DDI, the higher the dietary adequacy), “mixed” (significance and direction of the association varied depending on subgroups or type of DDIs that were used), “null” (no association), or “negative” (the higher the DDI, the lower the dietary adequacy). The association between DDIs and any health outcome was described as “favorable” (e.g., the higher the DDI, the lower the risk for metabolic syndrome or the lower the risk for wasting), “mixed” (significance and direction of the association varied depending on subgroups or type of DDIs used), “null” (no association), or “unfavorable” (e.g., the higher the DDI, the higher the risk for obesity).

## Results

### Comprehensive inventory of dietary diversity indicators

**FIGURE 1:**
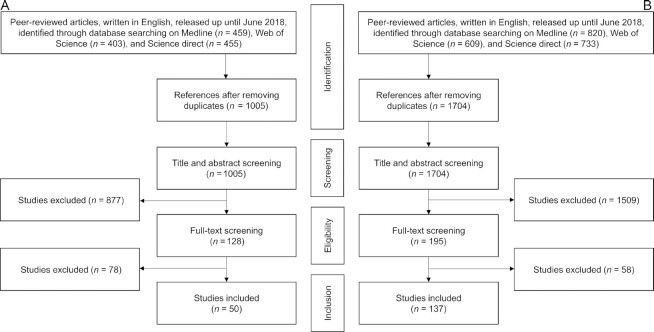
Systematic literature review flowchart for article selection. (A) Flowchart for selection and inclusion of studies in the systematic review of the evidence of dietary diversity and its relation with measures of dietary adequacy. (B) Flowchart for selection and inclusion of studies in the systematic review of the evidence of dietary diversity and its relationship with health outcomes.

Based on the 2 structured search strategies, 50 articles studying the association between dietary diversity and dietary adequacy and 137 articles studying the association between dietary diversity and any health outcomes were included (**Figure 1**). Cross-referencing the 2 sets of papers led to 161 unique studies published from 1989 to June 2018. From these, 4 types of DDI have been identified: the “food item–based indicators” (FIIs), the “food group–based indicators” (FGIs), the “dietary guidelines–based indicators” (DGIs) and the “other indicators” (OIs). **Table 2** summarizes the characteristics of these 4 types of DDI, further described in the following sections.

**TABLE 2:** Summary of the characteristics of the 4 types of dietary diversity indicators identified from the 161 articles included in the systematic literature review^1^

Type of dietary diversity indicator	Number of studies	Description
Food item-based indicators (FIIs)	56	Simple count of the different food items consumed. Consuming the same food item 1 or more times scored 1; not consuming a food item scored 0. Most of the time, any food item was counted, except in 7 studies where only food items considered by the authors as healthy, unhealthy, or traditional were counted.
Food group–based indicators (FGIs)	106	Simple count of the different food groups² consumed. Most of the time, foods were classified in 5, 9, 10, or 12 groups. Most of the time, consuming some foods from the same group 1 or more times scored 1; not consuming a food group scored 0. In a few cases, intra-food-group diversity was taken into account (24–34).
Dietary guidelines–based indicators (DGIs)	11	Two subtypes:– Healthy Food Diversity (HFD): multiplication of the Berry index by the health value ofthe individual's diet (33–38)
		– Count of different food groups consumed with the minimum recommended numberof servings consumed to be included in the count (39–43)
Other indicators (OIs)	12	Four subtypes:
		– Indicators reflecting how the foods consumed were distributed: Berry index (35, 46, 47),QUANTIDD index (48, 49), Entropy index (50), and Dissimilarity index (46)
		– Indicators calculated as a ratio between the “variety” (defined by the authors aspercentage of different food items consumed) of some food groups and the variety of other food groups (51–54)
		– Composite score based on consumption of food groups and relative importance ofeach food group, with or without taking into account frequency of consumption (55, 56)
		– Functional Diversity: a complex indicator reflecting the diversity in nutrient composition of species (plant, livestock, and fish) consumed (47)

1 See Supplemental Results for more details about the “Other indicators”.

2 A food group is a collection of foods that share similar nutritional properties or biological classifications.

#### FIIs of dietary diversity

**FIGURE 2:**
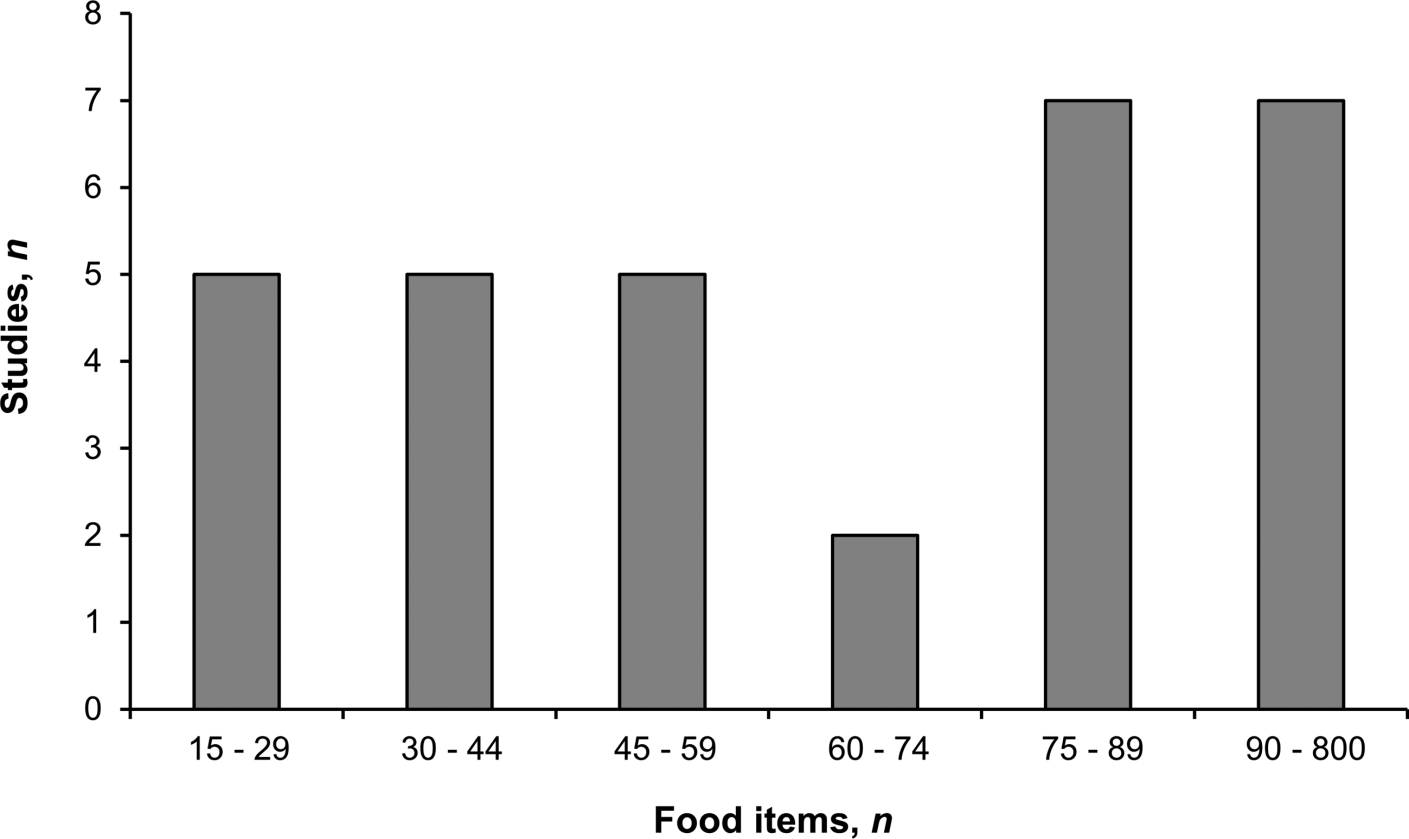
Distribution of studies according to the range of the theoretical maximum number of food items used in the food item–based indicators (when information available: *n* = 27 out of 56 studies).

The FIIs were based on the number of different food items consumed over a reference period. FIIs were used in 56 studies and were usually a simple count of food items that had been consumed (*n* = 49). In 7 studies, the FII was also a simple count of food items but only those considered by the authors as healthy (17–22), unhealthy (19–21), or traditional (23). We observed a large variability across studies in the number of different food items included in the FIIs (**Figure 2**), as well as variations in the reference periods (25 studies covered periods of 1–7 d, while 29 covered periods ranging from 7 d to 1 y).

#### FGIs of dietary diversity

**FIGURE 3:**
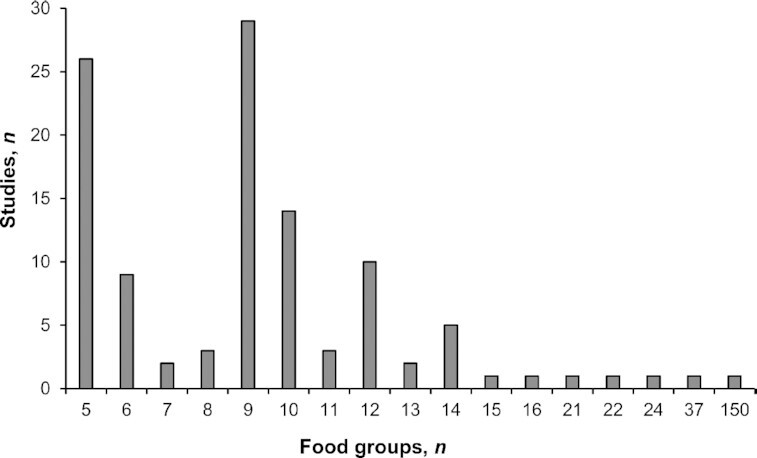
Distribution of studies according to the number of food groups used in the food group–based indicators (when information available: *n* = 102 out of 106 studies).

The FGIs were based on the number of different food groups consumed over a reference period, without allocating any weight to food groups. The way food groups were delineated varied greatly across the studies (resulting in varied numbers of food categories, of food items within categories, and of level of heterogeneity within the food groups). We decided to keep authors’ food groups, even when they were debatable. FGIs were used in 106 studies and were usually a simple count of the different groups consumed over a reference period (*n* = 90). Another approach was to first assess the diversity of food consumed among 5 main food groups, weighted by intra-food-group diversity (24–34). We observed a large variability across studies in the number of food groups considered (**Figure 3**). In the 55 studies that reported the rule to count a group in the indicator, we found large differences in the way to set the minimum amount (e.g., 15 or 25 g), or the serving size (e.g., half a serving or a serving), or the frequency of food consumption (e.g., once a week or once a day).

#### DGIs of dietary diversity

The DGIs assessed the number of different food groups consumed over a reference period and allocated different weights to each food group according to national food-based dietary guidelines or international recommendations. DDIs were used in only 11 studies and the most frequently used was the Healthy Food Diversity (HFD) index. First developed for the German context (35, 36) and later adapted to the US context (37–40), the HFD index is calculated by multiplying the Berry index (defined as 1 minus the sum of the squares of the share of each food in the total amount of energy intake) by the “health value” of the diet of each individual (calculated using health factors attributed to food groups according to the food-based dietary guidelines). Another type of DGI was based on the count of different groups consumed over a reference period, but in order for a food group to be counted in the index, individuals had to eat the minimum recommended number of servings according to national dietary guidelines (e.g., 3 servings daily for vegetables). This kind of DGI was used in the United States (41, 42), Sri Lanka (43, 44), and Malaysia (45).

#### OIs of dietary diversity

The OIs, which relied on different concepts, were used in 12 studies (see **Supplemental Results** for more details). Briefly, 1 main type of OI was used in 6 studies to reflect the distribution of foods consumed, like the Berry (or Simpson) index (35, 46, 47), the QUANTIDD index (48, 49), the Entropy index (50), and the Dissimilarity index (46). The other main type of OI was based on the ratio between the “variety” (defined by the authors as the percentage of different food items consumed) within some food groups and the “variety” within other food groups (51–54). Another type of OI was a composite score similar to a DDI but with different weights arbitrarily attributed to food groups (55, 56). Last, 1 OI was called the Functional Diversity, a complex indicator reflecting the diversity in the nutrient composition of species (plant, livestock, and fish) consumed by each individual (47).

#### Use of indicators of dietary diversity over time and across contexts

**FIGURE 4:**
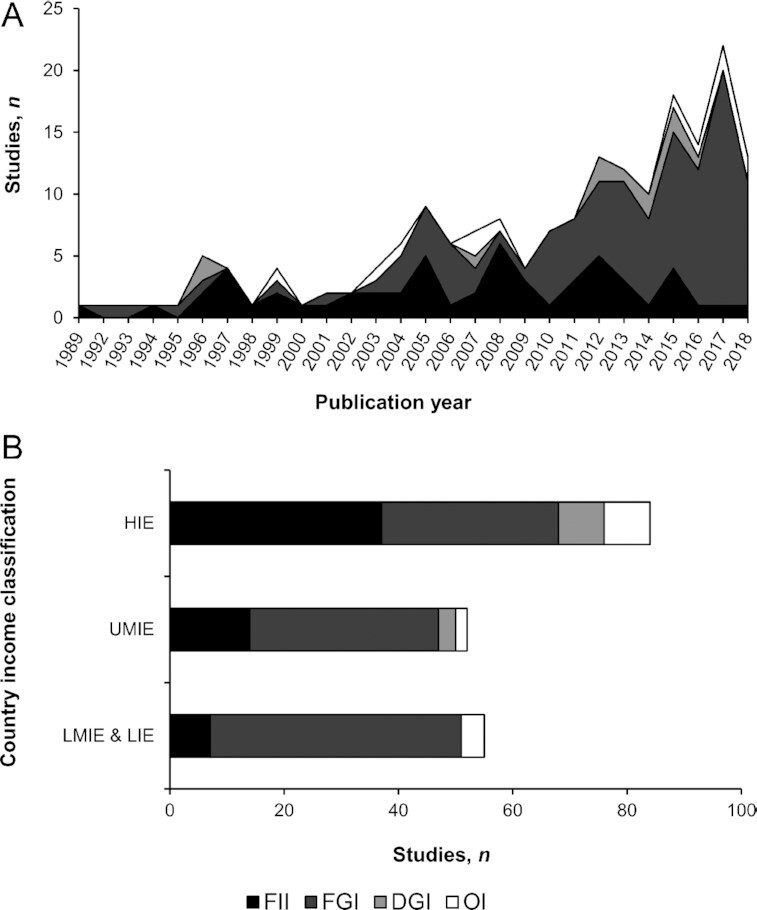
Number of studies using the different types of dietary diversity indicators for adolescents and adults across the 161 articles included in this review. (A) Number of studies per type and per year. (B) Number of studies per type and per country income classification. DGI, dietary guidelines–based indicator; FGI, food group–based indicator; FII, food item–based indicator; HIE, high-income economies; LIE, low-income economies; LMIE, lower-middle-income economies; OI, other indicator; UMIE, upper-middle-income economies.

A first analysis indicated a growing use of DDIs with time (**Figure 4**A), with a higher use of these indicators occurring in the context of high-income economies (HIEs) as compared with low-, lower-middle-, and upper-middle-income economies (LIEs, LMIEs, and UMIEs, respectively; Figure 4B). A second analysis of the 161 articles that took the type of DDI into account showed that FIIs and FGIs were the most frequent types of indicators. While their use began at the same time, with a similar occurrence of use over time, a shift in 2010 resulted in FGIs becoming the current most frequently used type of indicator (Figure 4A). In terms of country income classification, studies in the contexts of LIEs, LMIEs, and UMIEs have mainly used FGIs over the other types of DDI, whereas the use of the 4 types of DDI was more balanced in HIEs (Figure 4B).

### Review on the relation of DDIs with dietary adequacy

**FIGURE 5:**
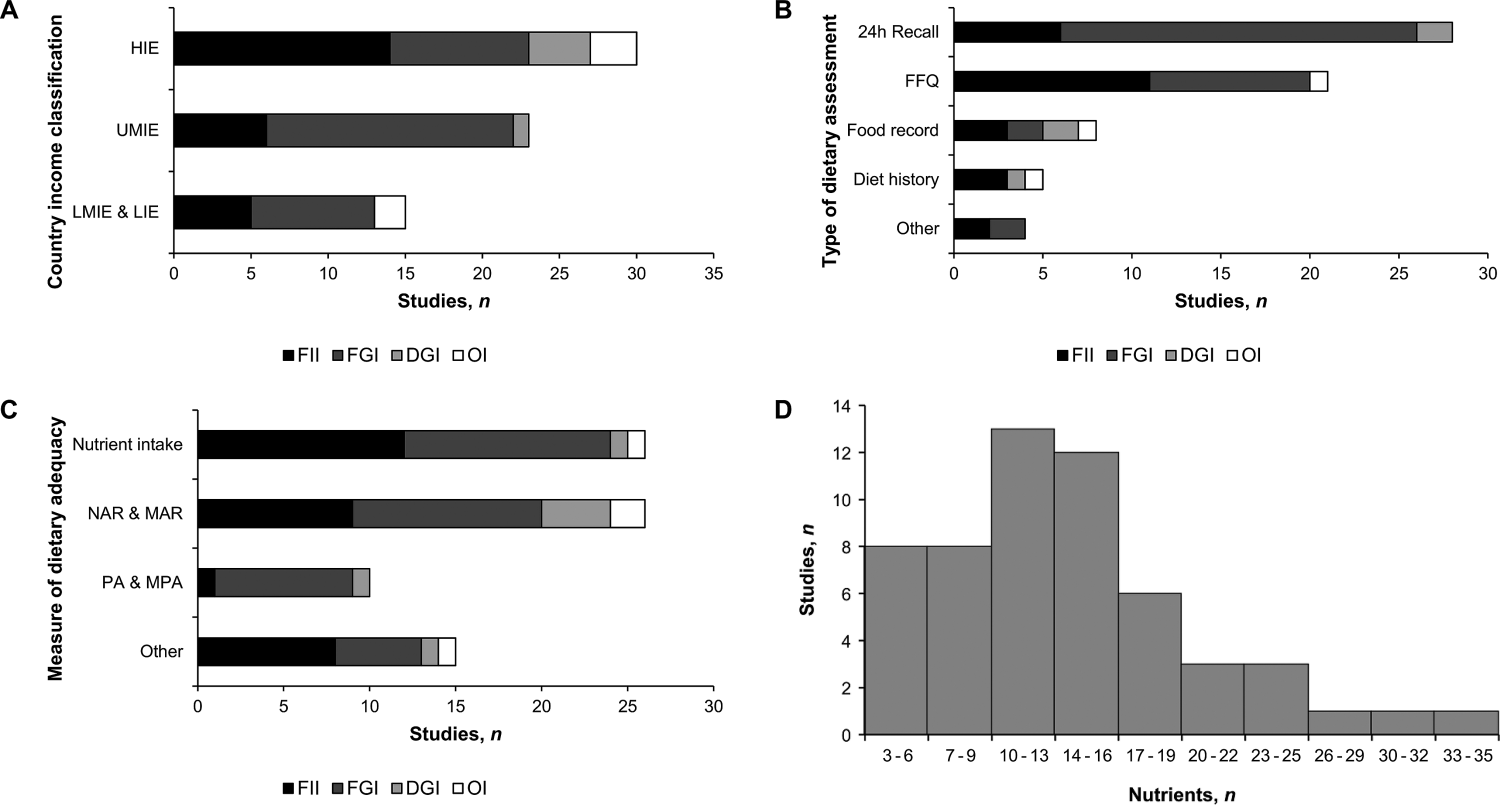
Studies investigating the relation between dietary diversity indicators and dietary adequacy (*n =* 50) by country income classification (A), type of dietary assessment (B), and measure of dietary adequacy(C). (D) Number of nutrients used in the measures of dietary adequacy. DGI, dietary guidelines–based indicator; FGI, food group–based indicator; FII, food item–based indicator; HIE, high-income economies; LIE, low-income economies; LMIE, lower-middle-income economies; MAR, mean adequacy ratio; MPA, mean probability of adequacy; NAR, nutrient adequacy ratio; OI, other indicator; PA, probability of adequacy; UMIE, upper-middle-income economies.

Fifty studies assessed the association between a DDI and a measure of dietary adequacy (**Supplemental Table 1**). Most of the studies used a cross-sectional design, were located in HIEs and UMIEs (**Figure 5**A), and calculated DDIs from either 24-h recalls or food-frequency questionnaires (FFQs; Figure 5B). The FGIs were the most frequently used indicators (*n* = 32), followed by FIIs (*n* = 24), DGIs (*n* = 5), and OIs (*n* = 4). There was a wide variation in terms of measures of dietary adequacy (Figure 5C) and in the number of nutrients used in the measures of dietary adequacy (Figure 5D).

**FIGURE 6:**
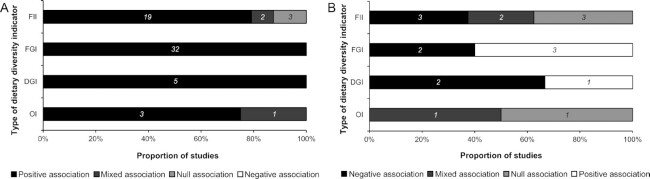
Results of studies investigating the relation between dietary diversity indicators and measures of dietary adequacy (*n =* 50) (A) or of excess nutrients (*n =* 14) (B), according to the type of indicator. DGI, dietary guidelines–based indicator; FGI, food group–based indicator; FII, food item–based indicator; OI, other indicator.

Among the 50 studies reviewed, all found some positive associations between the DDIs and the measures of dietary adequacy, except for 3 studies that found no associations with FIIs (57–59) and 3 others that found mixed associations with FIIs and OIs (19, 20, 46) (**Figure 6**A and **Supplemental Figure 1A**). Studies reporting mixed associations are those showing contradictory results (either significant or not and positive or negative associations) according to age groups (19) or to the type of DDIs used (20, 46) (see Supplemental Results for more details).

We found 19 studies that investigated the relation between DDIs and dietary adequacy with accounting for energy intake. When energy intake was controlled for, the relations were attenuated but still significant in 7 studies (25, 28, 60–64), became nonsignificant in 2 studies (58, 59), and were not attenuated in 2 studies (37, 47). However, while higher energy intake has long been recognized as a potential confounding factor when studying the relation between micronutrient intakes and indexes of overall diet quality (7), it could also play a mediating role in that relation. Indeed, some studies have shown that higher food and energy intakes come along with higher variety of foods consumed (65–67).

Only 14 studies explored how DDIs were associated with nutrients that can be unhealthy if consumed in excess, like SFAs or added sugars (called excess nutrients in the rest of the article). Four studies found that DDIs were negatively related to excess nutrients (i.e., the higher the DDIs, the lower the intakes) (37, 57, 68, 69), 4 found positive associations (41, 70–72), and 6 found mixed (17, 21, 35, 46) or null (58, 73) associations. There was no clear pattern to explain these contrasting results (Figure 6B and Supplemental Figure 1B). Two DDIs provided interesting results. First, the Dissimilarity index was found to be negatively related to various food-based diet-quality indicators, providing a specific measure of “unhealthy dietary diversity” due to its design which incorporated 12 different foods likely to have cardiometabolic effects (46). Next, the HFD index seemed to be the most suitable DDI to provide a specific measure of “healthy dietary diversity", being both positively related to measures of dietary adequacy and inversely related to excess nutrients (35, 37).

### Review on the associations of DDIs with health outcomes

The 137 studies included in the review on the association between DDIs and health outcomes were further classified into 6 categories of health outcomes: *1*) body weight and body composition (*n =* 60), *2*) noncommunicable diseases (NCDs) and intermediate biomarkers of health (*n =* 41), *3*) biomarkers of nutritional status (*n =* 19), *4*) mental health and cognitive functions (*n =* 17), *5*) mortality (*n =* 10), and *6*) other health outcomes (*n =* 18).

**FIGURE 7:**
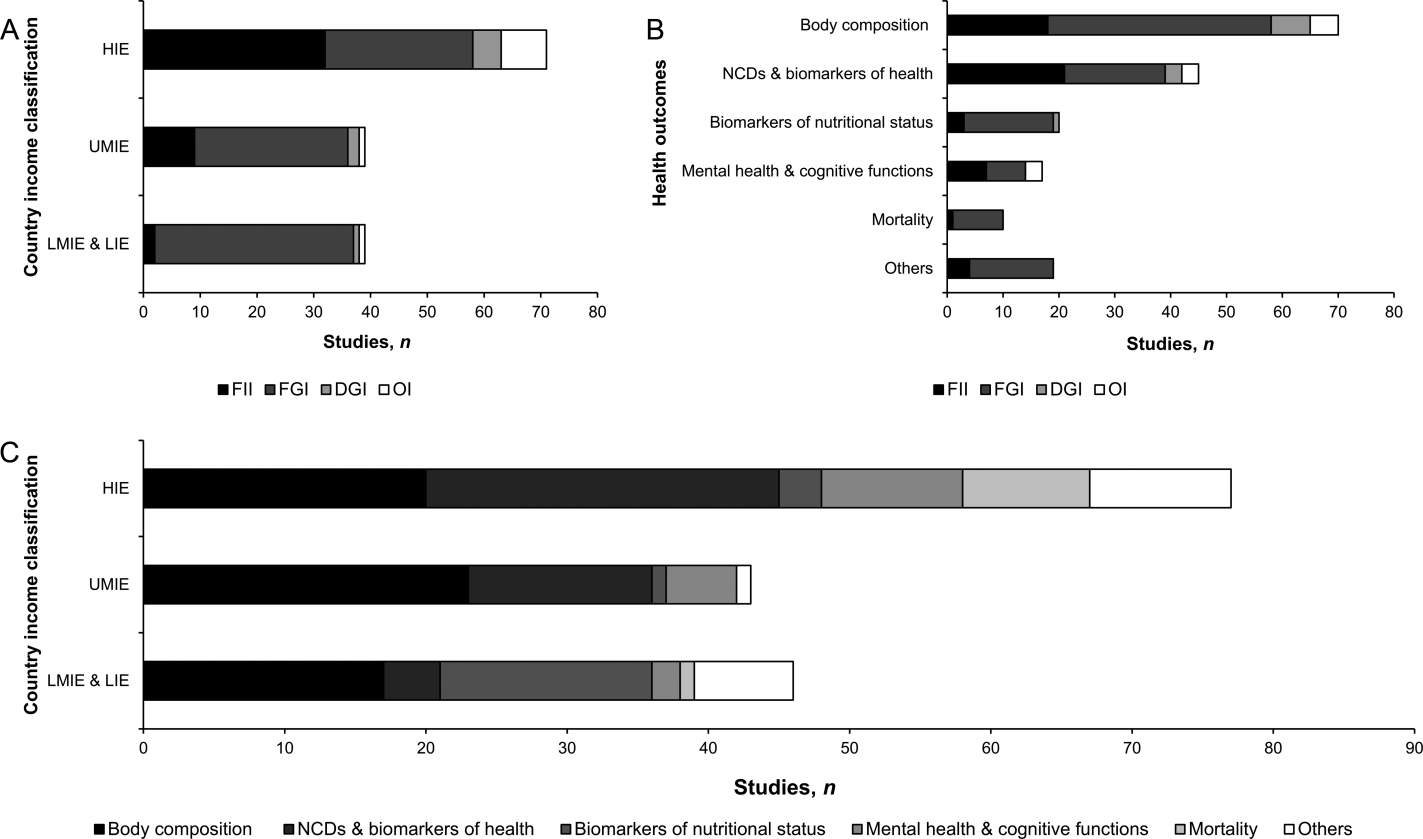
Studies investigating the associations between dietary diversity indicators and health outcomes (*n =* 137) by country income classification (A) or health outcomes (B). (C) Number of health outcomes studied by country income classification. DGI, dietary guidelines–based indicator; FGI, food group–based indicator; FII, food item–based indicator; HIE, high-income economies; LIE, low-income economies; LMIE, lower-middle-income economies; NCD, noncommunicable diseases; UMIE, upper-middle-income economies.

**FIGURE 8:**
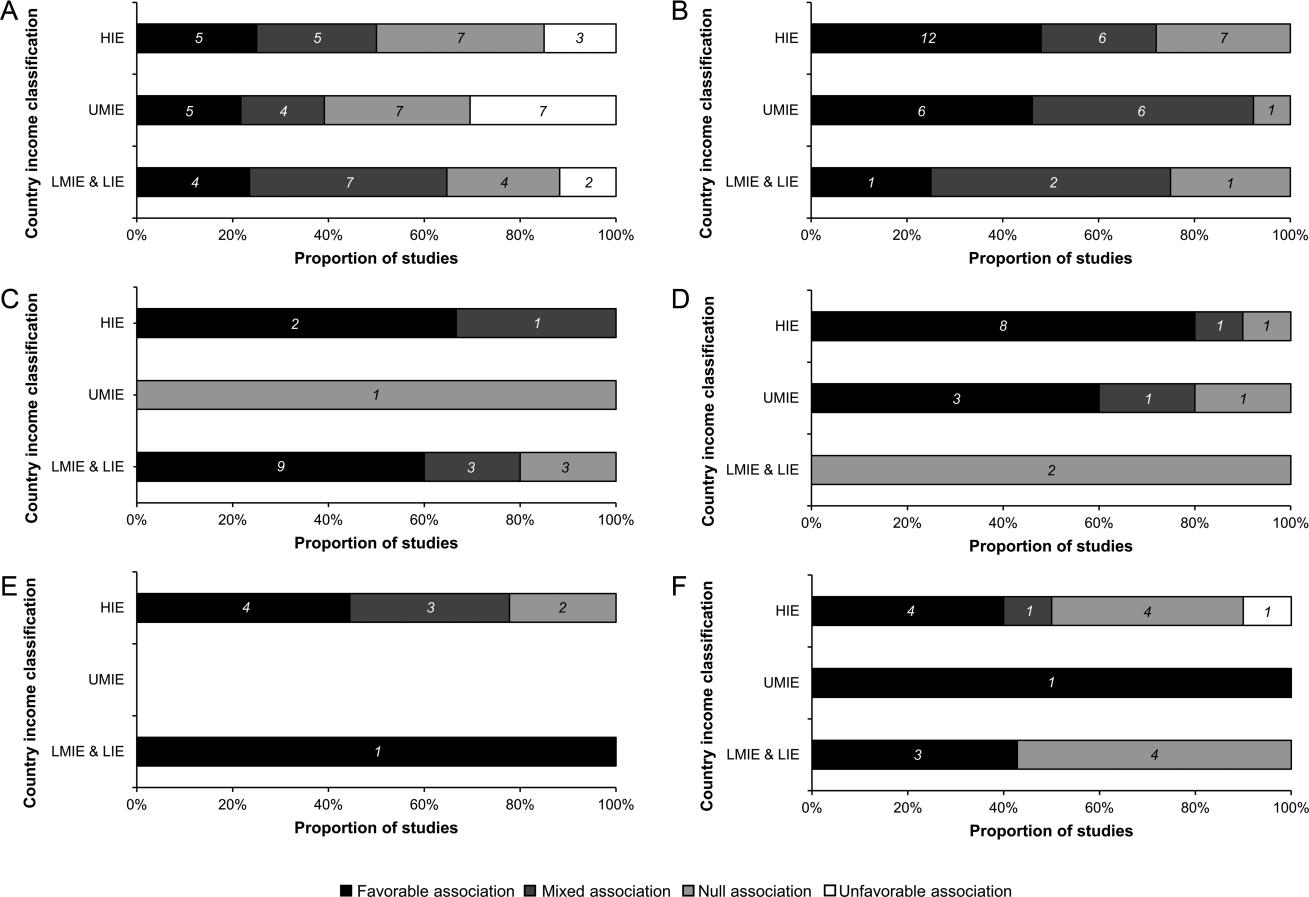
Results of studies investigating the associations between dietary diversity indicators and body composition (*n =* 60) (A), noncommunicable diseases and intermediate biomarkers of health (*n =* 41) (B), biomarkers of nutritional status (*n =* 19) (C), mental health and cognitive functions (*n =* 17) (D), mortality (*n =* 10) (E), and other health outcomes (*n =* 18) (F), according to country income classification. HIE, high-income economies; LIE, low-income economies; LMIE, lower-middle-income economies; UMIE, upper-middle-income economies.

Most studies used a cross-sectional design and were located in HIEs (**Figure 7**A). While body-weight and body-composition outcomes have been studied across different economical categories of countries, the number of studies investigating other health outcomes varied according to those categories (Figure 7C). For example, NCDs and intermediate biomarkers of health have been studied in HIEs more frequently than in UMIEs, and in UMIEs more than in LMIEs and LIEs, whereas biomarkers of nutritional status have been more frequently studied in LMIEs and LIEs than in UMIEs and HIEs. FGIs were by far the most frequently used indicators (*n* = 89), followed by FIIs (*n* = 43), DGIs (*n* = 8), and OIs (*n* = 10). The predominance of FGIs was noticeable across the 6 categories of health outcomes, except for NCDs and intermediate biomarkers of health, and for mental health and cognitive functions outcomes where FIIs were used just as much (Figure 7B). Thus, the analysis of the association between DDIs and health outcomes was driven by the economical context of the studies (**Figure 8**) rather than by the types of DDIs (**Supplemental Figure 2**). The following sections present a global analysis of each health outcome (except for the category “other health outcomes”) but their detailed presentation can be found in the Supplemental Results and **Supplemental Tables 2–7**.

#### DDIs and body weight and body composition

Fifty-one studies evaluated the association between DDIs and outcomes that were related to excess body weight (weight gain, being overweight, or obesity); there was a similar proportion of studies finding favorable, mixed, null, or unfavorable associations. Of note, the proportion of studies finding an unfavorable association was higher in UMIEs compared with other contexts (Figure 8A). Only 6 studies used a longitudinal design, finding either a favorable (40, 74), mixed (46, 75), null (76), or unfavorable (51) association. Furthermore, only 2 of them had a sample size >1000 participants and used multi-adjusted regressions (46, 76). Nevertheless, 3 studies using a specific DDI provided interesting results that deserve to be highlighted. The food variety ratio could be considered as corresponding to some “(un)balanced dietary diversity”: defined by the “variety” of snacks divided by the “variety” of grains and meats, it was found to be associated with an increased risk of becoming overweight (51) or obese (52), while defined by the “variety” of vegetables divided by the “variety” of sweets, snacks, condiments, lunch and dinner entrées (e.g., beef, fried fish, pizza), and carbohydrates, it was found to be inversely associated with body fatness (54).

There were only 9 studies related to being undernourished [defined as a BMI (kg/m^2^) <18.5 among adults], with similar proportion of studies finding favorable, mixed, or null associations. The sole study using a longitudinal design found a favorable association of increased dietary diversity over undernourishment (77). These 9 studies represented about half of the studies conducted in LMIEs and LIEs (Figure 8A).

#### DDIs and NCDs and intermediate biomarkers of health

Forty-one studies evaluated the association between DDIs and NCDs and intermediate biomarkers of health, with half of them finding a favorable association and the other half finding a mixed or null association. The number of studies and their types of results differed by country income classification (Figure 8B) and by type of DDI (Supplemental Figure 2B). Only 5 studies used a longitudinal design with large sample sizes and multi-adjusted regressions, of which 1 study found a favorable association with diabetes (78), 3 found null associations with diabetes (21, 46, 79) and 1 study found a mixed association with cardiovascular disease (80).

#### DDIs and biomarkers of nutritional status

Nineteen studies evaluated the association between DDIs and biomarkers of nutritional status, with about half of them finding a favorable association and the other half finding a mixed or null association. Most of these studies were conducted in LMIEs and LIEs (Figure 8C) and used FGIs (Supplemental Figure 2C). Only 3 studies used a longitudinal design with moderate sample sizes and multi-adjusted regressions, finding either favorable (81), mixed (82) or null (83) associations with anemia.

#### DDIs and mental health and cognitive functions

Seventeen studies evaluated the association between DDIs and mental health and cognitive functions, about two-third finding a favorable association and one-third finding a mixed or null association. Most of these studies were conducted in HIEs (Figure 8D). Only 3 studies used a longitudinal design with moderate sample sizes and multi-adjusted regressions, finding a favorable association with cognitive function (48, 49, 84).

#### DDIs and mortality

Ten studies used a longitudinal design with medium to large sample sizes and multi-adjusted regressions to evaluate the association between DDIs and all-cause or cause-specific mortality. Half found a favorable association (83–87) and the other half a mixed (21, 76, 88) or null (89, 90) association. The studies were mainly conducted in HIEs (Figure 8E) and mainly used FGIs (Supplemental Figure 2E).

## Discussion

To the best of our knowledge, this is the first systematic scoping review presenting an extensive and comprehensive inventory of DDIs developed for adolescents and adults worldwide and summarizing the evidence of the associations between these indicators and *1*) some measures of the dietary adequacy and *2*) some health outcomes. The first finding of this review is that there is a large number of DDIs used in the literature, greatly varying in their structure. While almost all DDIs were found to be positively associated with some measures of dietary adequacy, their inconsistent relation with nutrients to limit was of concern, which underlines their limit to reflect overall diet quality. Overall, higher DDIs tended to be associated with benefits for various health outcomes, except in contexts such as HIEs and UMIEs where higher DDIs were found to be associated with higher risk of overweight and obesity. However, many studies found mixed or null results. In addition, only one-quarter of the studies used longitudinal designs.

### Three decades of development of DDIs

The results of this review should be interpreted in the historical context of the evolution of DDIs. Originating in the late 1980s, DDIs have been extensively developed and used, as dietary diversity is recognized as a key feature of high-quality diets. Nevertheless, due to the lack of consensual definition of dietary diversity (or variety), a wide range of DDIs has been proposed with many more variations in terms of format (e.g., number of foods or food groups, based on single 24-h recall or FFQ) rather than concept (the vast majority of DDIs relied on counting foods or food groups consumed over a period of time).

A shift occurred in the late 2000s, with several research projects aiming to develop and validate simple FGIs based on single 24-h recalls for women of reproductive age that remain relevant and consistent across different contexts and over time (60). These methodological efforts are ongoing (12, 91).

Because some countries face a dramatic lack of expertise, infrastructure, and financial support (92), the Women's Dietary Diversity Score (93) and the Minimum Dietary Diversity for Women of Reproductive Age (11) have paved the way for simple assessments of dietary diversity at the population level. Subsequently, they have favored the implementation of nutrition and health interventions, and also of nutrition-sensitive agriculture interventions, since these DDIs can be used to assess changes in the populations’ diet before and after an intervention or to compare communities undergoing an intervention with control communities (94, 95). Nevertheless, these 2 FGIs for women of reproductive age were validated as proxies of nutrient adequacy but not as proxies of moderation. While LIEs and LMIEs are undergoing an accelerated (although heterogeneous) nutrition transition characterized by increased intakes of unhealthy fats, refined carbohydrates, and added sugar (96), focusing solely on DDIs may be a major limitation in these contexts. Indeed, concerns regarding the excessive intake of certain nutrients and foods in LIEs and LMIEs were raised 20 y ago, questioning the need of a shift in the definition of dietary quality to include both concepts of nutrient deficiency and overnutrition (6).

### DDIs and diet quality

This review found that the ability of DDIs to reflect overall diet quality was limited. Only 14 out of 50 studies explored how DDIs were associated with nutrients to limit, and only 4 studies found that DDIs could be considered as a proxy of moderation (37, 57, 68, 69).

In many HIEs, the food-based dietary guidelines changed from presenting separately the traditional concepts of dietary diversity, adequacy, moderation, and balance to including all these dimensions in each food-group recommendation (97). This shift was therefore also noticeable in the diet quality indices constructed from food-based dietary guidelines. For example, while US dietary guidelines evolved and specified the most advantageous types of variety, the original Healthy Eating Index (HEI), featuring dietary variety as a separate component, evolved into the HEI-2005, which no longer recognizes dietary variety as a separate component but instead includes new components that reflect the types of variety deemed to be the most beneficial (98).

A recent systematic review focusing on LIEs and LMIEs reported that individual DDIs were good proxies of nutrient adequacy, but also highlighted the necessity of accounting for moderation dimensions (99). The authors recommended evaluating healthy and unhealthy diet components separately, since using a single score for both components might reduce their ability to reflect diet quality and underestimate their associations with health outcomes (99). The development of a targeted systematic review and meta-analysis focusing on the strength of associations between DDIs and measures of diet quality would be of interest to better understand to what extent DDIs could reflect overall diet quality.

The use of simple DDIs may be convenient and relevant in many contexts, but such indicators should not be overinterpreted as reflecting other dimensions of dietary quality like moderation. Several DDIs providing more specific/detailed measurements of dietary diversity have been flagged throughout this review. The HFD index provides a specific measure of “healthy dietary diversity” (35, 37), the Dissimilarity index a specific measure of “unhealthy dietary diversity” (46), and the food variety ratio a specific measure of “(un)balanced dietary diversity” (51, 52, 54). However, these indicators were only used in specific contexts (Germany and United States for the HFD index, United States for the Dissimilarity index, and United States and Hong Kong for the food variety ratio) and their performances in other contexts would need to be tested. Furthermore, these indicators require intensive quantitative dietary data collection and sometimes demand high-skilled resources for data processing (HFD index and Dissimilarity index), thus limiting their use.

### Dietary diversity, food-group diversity, and health outcomes

This review found that the associations between DDIs and health outcomes were largely inconsistent. Less than one-quarter of the studies included in this review were based on a longitudinal design, which is more conducive than cross-sectional or case-control designs to establish the relation with health-related outcomes. When considering the sole studies based on a longitudinal design with a large sample size and multi-adjusted regression analysis, most of them found mixed or null associations between DDIs and health outcomes. Also, DDIs were mostly assessed from a single 24-h recall, multiple 24-h recalls, or FFQs. Therefore, some studies explored the relation with health outcomes using a 1-d dietary diversity while others used a “usual” dietary diversity. These differences could affect the nature and magnitude of the associations.

Although the design and context of the studies are important to take into account, it is also important to consider the conceptual framework linking the dietary diversity to health outcomes. In most studies, it is quite unclear whether the authors used a DDI because they hypothesized a benefit of dietary diversity in their particular study setting or because they simply took a DDI as a general proxy for diet quality.

About half of the studies analyzing the associations between DDIs and NCDs, intermediate biomarkers of health, or mortality found mixed or null results. For these health outcomes, the consumption of specific food groups seemed to be more important than the overall diversity of the diet. For example, fruits and vegetables contain numerous nutrients, phytochemicals, and other unidentified compounds that are likely to act synergistically via several biological mechanisms in reducing the risk of chronic diseases and premature mortality. Some studies found that vegetable diversity was more strongly associated with a decreased risk of cancer than total dietary diversity (see Supplemental Results) (100–105). A recent dose–response meta-analysis of 95 studies found that the consumption of several types of fruits and vegetables was individually and inversely associated with coronary artery disease, stroke, cardiovascular diseases, total cancer, and all-cause mortality (106).

Regarding the associations between DDIs and body weight and body composition, the results were highly heterogeneous, but a majority of studies found mixed or null results. Two previous reviews reported that dietary diversity was inconsistently associated with body adiposity in diverse populations (8) or not significantly associated with BMI status (9), and both questioned the definition of dietary diversity and the construction of the DDIs. According to Vadiveloo et al. (8), associations with body adiposity were more consistent when dietary diversity focused on recommended and low-energy foods alone (i.e., foods that do not increase the odds of overweight and obesity) or when dietary diversity focused on less healthful, energy-dense foods alone (i.e., foods increasing the odds of overweight and obesity). This suggests that the diversity within specific food groups is of higher importance than the overall diversity of the diet. Recently, the American Heart Association stated that existing evidence does not support greater dietary diversity as an effective strategy to promote healthy body weight, and emphasized the need for further data to formulate adequate recommendations on dietary diversity (10).

### Limitations

Some limitations of this review have to be mentioned. Despite the use of the PRISMA-ScR guidelines, multiple structured search strategies, and PICOS criteria, this systematic scoping review may not be exhaustive due to the broad nature of the initial objective to provide an inventory of the DDIs and their associations with the dietary adequacy and/or various health outcomes. Additional and more focused structured search strategies may have led to greater completeness. However, we believe the method we used struck the right balance between feasibility and completeness. Another limitation is that the review only included articles published in English. Scoping reviews usually provide a descriptive overview without critically appraising individual studies, and the need for quality assessment of included studies in the scoping review process is still debated (13). Because this systematic scoping review encompasses very heterogeneous studies in terms of objectives, outcomes, designs, and statistical models, it would have been difficult to provide a consistent quality assessment and we decided not to assess the validity or the quality of the studies nor that of the indicators used. However, when focusing on health outcomes, we chose to describe the results of studies that used a longitudinal design with moderate/large sample sizes and multi-adjusted regressions. Nevertheless, only rigorous systematic reviews and meta-analysis would enable drawing conclusions as to what extent DDIs are associated with measures of diet quality and specific health outcomes.

### Conclusions

The ability of DDIs to reflect diet quality was found to be principally limited to micronutrient adequacy. Furthermore, DDIs do not readily relate to health outcomes. Echoing the appeals from different authors, our findings call for the development and rigorous evaluation of a global diet quality index for use in LIEs and LMIEs (99, 107, 108). Developing sound, easy-to-collect, and easy-to-use indicators of “healthy dietary diversity”, “unhealthy dietary diversity”, and “(un)balanced dietary diversity” (which could be analyzed in conjunction or combined into a global diet quality index) could be a way forward to measuring the impact of nutrition policies and programs aiming to tackle malnutrition in all its forms and track progress made to achieve Sustainable Development Goal number 2 by 2030.

## Supplementary Material

nmab009_Supplemental_File

## Notes

This manuscript evolved from content for an invited presentation made at a 2018 meeting convened by Intake—Center for Dietary Assessment, at which YM-P was an invited speaker and Intake-funded participant. Open-access charges were supported by the Bill & Melinda Gates Foundation, Seattle, Washington.

Author disclosures: The authors report no conflicts of interest.

Supplemental Figures 1 and 2, Supplemental Tables 1–7, and Supplemental Results are available from the “Supplementary data” link in the online posting of the article and from the same link in the online table of contents at https://academic.oup.com/advances/.

Abbreviations used: DDI, dietary diversity indicator; DGI, dietary guidelines–based indicator; FFQ, food-frequency questionnaire; FGI, food group–based indicator; FII, food item–based indicator; HFD, Healthy Food Diversity; HIE, high-income economy; LIE, low-income economy; LMIE, lower-middle-income economy; NCD, noncommunicable disease; OI, other indicator; PICOS, Population, Interventions, Comparators, Outcomes, and Study; PRISMA-ScR, Preferred Reporting Items for Systematic Reviews and Meta-Analyses Extension for Scoping Reviews; UMIE, upper-middle-income economy.
