# Supplementary material for: Dietary Diversity Indicators and Their Associations with Dietary Adequacy and Health Outcomes: A Systematic Scoping Review
Source: Adv Nutr. 2021 Mar 3;12(5):1659–72. doi: 10.1093/advances/nmab009 (PMC8483968; doi:10.1093/advances/nmab009)
Supplement: nmab009_Supplemental_File [file nmab009_supplemental_file.docx]

**Dietary diversity indicators and their associations with dietary adequacy and health outcomes: a systematic scoping review**

Verger et al*.*

**Online Supporting Materials**

**Contents:**

**Pages 2-7: Supplemental Results**

**Page 8: Supplemental Figure 1.** Associations by country income classification of studies investigating the relationships between dietary diversity indicators and measures of nutritional adequacy of the diet and excess nutrients.

**Page 9: Supplemental Figure 2.** Associations by type of dietary diversity indicators of studies investigating the associations between dietary diversity indicators and health outcomes.

**Pages 10-24: Supplemental Table 1.** Summary of 50 studies evaluating the association between dietary diversity indicators and measures of dietary adequacy of the diet in participants aged 10 years and older.

**Pages 25-35: Supplemental Table 2.** Summary of 60 studies evaluating the association between dietary diversity indicators and body weight and body composition in participants aged 10 years and older.

**Pages 36-44: Supplemental Table 3.** Summary of 41 studies evaluating the association between dietary diversity indicators and non-communicable diseases and intermediate biomarkers of health in participants aged 10 years and older.

**Pages 45-48: Supplemental Table 4.** Summary of 19 studies evaluating the association between dietary diversity indicators and biomarkers of nutritional status in participants aged 10 years and older.

**Pages 49-51: Supplemental Table 5.** Summary of 17 studies evaluating the association between dietary diversity indicators and mental health and cognitive functions in participants aged 10 years and older.

**Pages 52-53: Supplemental Table 6.** Summary of 10 studies evaluating the association between dietary diversity indicators and mortality in participants aged 10 years and older^1^.

**Pages 54-57: Supplemental Table 7.** Summary of 18 studies evaluating the association between dietary diversity indicators and other health outcomes in participants aged 10 years and older.

**Pages 58-68: Supplemental References**

**Supplemental Results**

**Comprehensive inventory of dietary diversity indicators**

***Other indicators of dietary diversity***

One main type of OI was used in six studies to reflect the distribution of foods consumed. Three studies used the Berry (or Simpson) Index (1–3), which is calculated as follows:

$$Berry \left( or Simpson \right)Index=1- \sum s_{i}^{2}$$

where *s* is the quantitative share of single food *i* in terms of weight. Two studies used the QUANTIDD index (4,5), which is calculated as follows:

$$QUANTIDD= \frac{1- \sum_{j}^{n} s_{j}^{2}}{1-\frac{1}{n}}$$

where *s* is the proportion of food group *j* that contribute to total energy or nutrient intake and *n* is the number of food groups. One study used the Entropy index (6) which is calculated as follows:

$Entropy Index= \sum_{j}^{n} w_{j}\ln\left( \frac{1}{w_{j}} \right)$

where *s* is the quantitative share of single food group *j* in terms of weight and *n* is the number of food groups. One study used the Dissimilarity index (2), which is determined by the average distance of pairwise comparisons of all food items consumed by each participant, using 12 different food attributes based on likely evidence for their associations on cardio-metabolic health. The pairwise comparison of two food items x and y was based on the Jaccard distance, defined as (B_x+C_y)/(A_xy+B_x+C_y), where Axy = number of attributes shared by food items x and y; Bx = number of attributes unique to x; Cy = number of attributes unique to y.

The other main type of OI was based on the ratio between the “*variety*” (defined by the authors as the percentage of different food items consumed) within some food groups and the “*variety*” within other food groups and was used in four studies, but with different arrangements: ratio of the “*variety*” of snacks to the “*variety*” of grains and meats (7,8), or the “*variety*” of grains, meats, fruit, and vegetables (9), and ratio of the “*variety*” of vegetables to the “*variety*” of sweets, snacks, condiments, lunch and dinner entrées (e.g., beef, fried fish, pizza), and carbohydrates (10).

Another type of OI was based on the consumption of food groups over a reference period; one of the OIs took the frequency of consumption of each group into account and the other did not, and different weights were attributed to the groups according to their somewhat arbitrary nutritional value (11,12). Lastly, one OI was called the Functional Diversity, a complex indicator reflecting the diversity in the nutrient composition of species (plant, livestock, and fish) consumed by each individual (3).

**Review on the relationship of dietary diversity indicators with dietary adequacy**

Three studies found mixed associations between a DDI and a measure of dietary adequacy (2,13,14). Gregory et al. (14) used three different FIIs, summing only either recommended food items consumed, unrecommended food items consumed, or any unique food consumed; the FII summing unrecommended food items was not associated to any diet quality indicator, while the other two were positively associated. Otto et al. (2) used one FII, one OI representing how evenly foods consumed were distributed, and one OI representing how foods consumed were different based on their associations on cardio-metabolic health (Dissimilarity index), and found that their associations with diet quality indicators were respectively positive, null, and negative.

**Review on the associations of dietary diversity indicators with health outcomes**

***Dietary diversity indicators and body weight and body composition***

Among the 51 studies assessing excess body weight (weight gain, being overweight, or obesity), ten reported favorable associations of a diverse diet (15–20,10,21–23), 14 mixed associations (24–26,2,6,27–35), 15 null associations (36,14,37–49), and 12 unfavorable associations (i.e. higher risks of overweight, obesity, or weight gain associated with higher DDI values (13,50–53,12,7,8,54–57)).

Among the nine studies assessing undernutrition, four reported favorable associations (58–61), two mixed associations (62,63), and three null associations (64–66).

Stratifying the associations by country income classification showed that the nine studies assessing undernutrition represented about half of the studies conducted in LMIE and LIE (*n=*17) and that the proportion of studies finding an unfavorable association was higher in UMIE compared to other contexts (**Figure 8A**). Interestingly, all but one study that found unfavorable associations in UMIE used FGIs (**Supplemental Figure 2A**).

***Dietary diversity indicators and NCDs and intermediate biomarkers of health***

Ten studies focused on cancer, with six reporting favorable associations (67–72), two mixed associations (73,74), and two null associations (41,75). All of these studies used case-control designs, were mainly held in HIE (*n=*9), and used FIIs. Some of these studies explored variety within specific food groups and generally found stronger associations with cancer risk when compared to overall dietary diversity. For example, higher meat variety was associated with higher colon cancer risk (41,73) and laryngeal cancer risk (75), while higher vegetable variety was associated with lower colorectal cancer risk (67), laryngeal cancer risk (75), esophageal cancer risk (71), gastric cancer risk (68), and oral and pharyngeal cancer risk (69,70).

Ten studies focused on metabolic syndrome, with six reporting favorable associations (24,50,18,76–78) and four reporting mixed associations (14,51,43,79). All ten studies used a cross-sectional design, were mainly held in UMIE (*n=*9), and mainly used FGIs (*n=*7).

Nine studies focused on diabetes, with three reporting favorable associations (80–82), two mixed associations (83,84), and four null associations (2,9,85,86). While these studies were mostly held in HIE (*n=*7), there were differences in terms of study design (four studies were cross-sectional, one case-control, and four longitudinal) and types of DDIs (five studies exclusively used FGIs, two used FIIs, one used OI, and one used both FII and OI).

Five studies focused on hypertension, with one reporting a favorable association (87), two mixed associations (88,89), and two null associations (29,86).

Five studies focused on biomarkers of risk for metabolic syndrome, with two reporting favorable associations (1,15), two mixed associations (38,27), and one null association (39).

Two studies focused on cardiovascular disease, with one reporting a favorable association using a cross-sectional design (90) and one mixed association using a longitudinal design with large sample sizes and multi-adjusted regressions (91).

One study focused on non-alcoholic fatty liver diseases and reported a null association (92).

***Dietary diversity indicators and biomarkers of nutritional status***

Twelve studies focused on anemia, two of which incorporated the nutritional status of other micronutrients (folate, retinol and zinc), while five dealt with micronutrient status alone (folate, potassium, retinol, vitamins B12, C, and E, and zinc). Among the studies focusing on anemia, seven reported favorable associations (31,93–98), three mixed associations (99–101), and four null associations (30,42,102,103). Among the studies focusing solely on micronutrient status, four reported favorable associations (15,104–106) and three mixed associations (38,100,101).

***Dietary diversity indicators and mental health and cognitive functions***

Eight studies focused on mental health (e.g. depression, stress, or anxiety), six on cognitive function in the elderly, and three on anorexia nervosa treatment. Among the studies focusing on mental health, three reported favorable associations (107–109), two mixed associations (110,111), and three null associations (11,43,112). All the six studies focusing on cognitive function reported a favorable association (4,5,42,113–115). Three studies focused on the success of anorexia nervosa treatment, using longitudinal design but with very small sample sizes, and reported either a favorable association (116,117) or no association (118).

***Dietary diversity indicators and other health outcomes***

Studies that evaluated the association between DDIs and other health outcomes (**Supplemental Table 7**), were conducted evenly in HIE, LMIE, and LIE (**Figure 8F**), and primarily used FGIs (**Supplemental Figure 2F**). This group of studies was extremely heterogeneous in terms of health outcomes (from one to four studies on the same health outcome), study design, sample size (from 70 to 2509 participants), and methods of analysis:

- Four studies focused on birth outcomes, with two reporting favorable associations (31,93) and two null associations (112,119);
- Two studies focused on sarcopenia, with one reporting a mixed association (120) and one a unfavorable association (121);
- Two studies focused on leprosy, both reporting null associations (122,123);
- One study focused on bone mineral status and reported a null association (124);
- One study focused on combinations of BRCA mutations and reported a null association (125);
- One study focused on dark adaptation and reported a favorable association (126);
- One study focused on the severity of frailty among older persons and reported a favorable association (127);
- One study focused on functional disability and reported a favorable association (128);
- One study focused on leukocyte telomere length and reported a null association (129);
- One study focused on lower urinary tract symptoms and reported a null association (130);
- One study focused on nutritional status as assessed by Patient-Generated Subjective Global Assessments and reported a favorable association (131);
- One study focused on the symptomatology of breast cancer and reported a favorable association (107);
- One study focused on sustained visual and auditory attention and reported a favorable association (132).


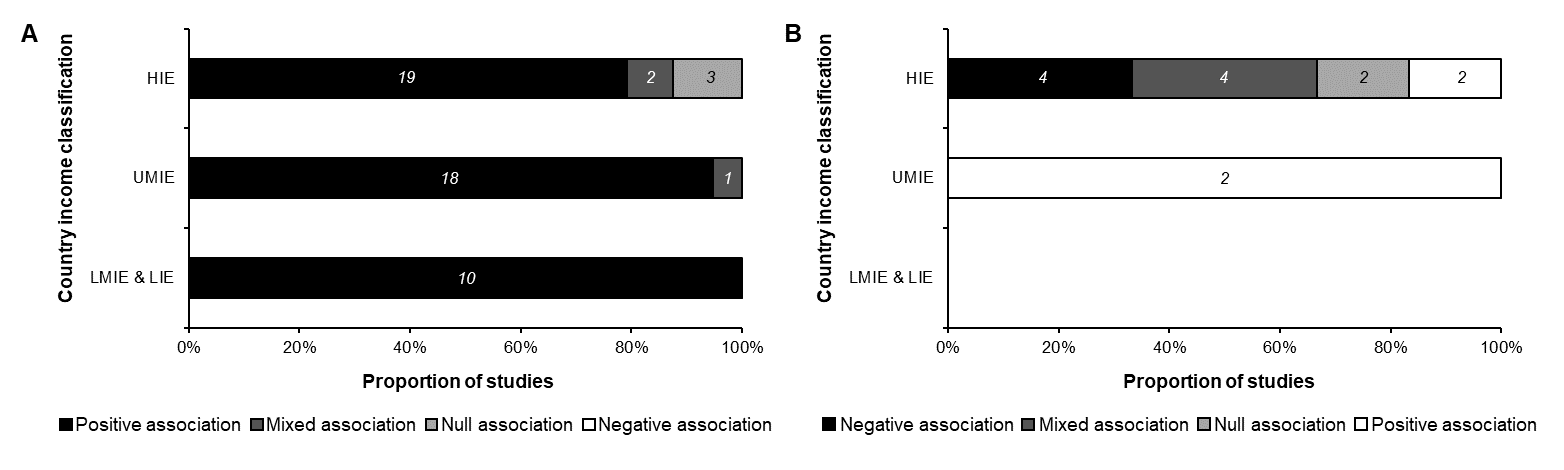


**Supplemental Figure 1.** Associations by country income classification of studies investigating the relationships between dietary diversity indicators and measures of nutritional adequacy of the diet (*n=*50, A) and excess nutrients (*n=*14, B). HIE: High-Income Economies. UMIE: Upper-Middle-Income Economies. LMIE: Lower-Middle-Income Economies. LIE: Low-Income Economies.


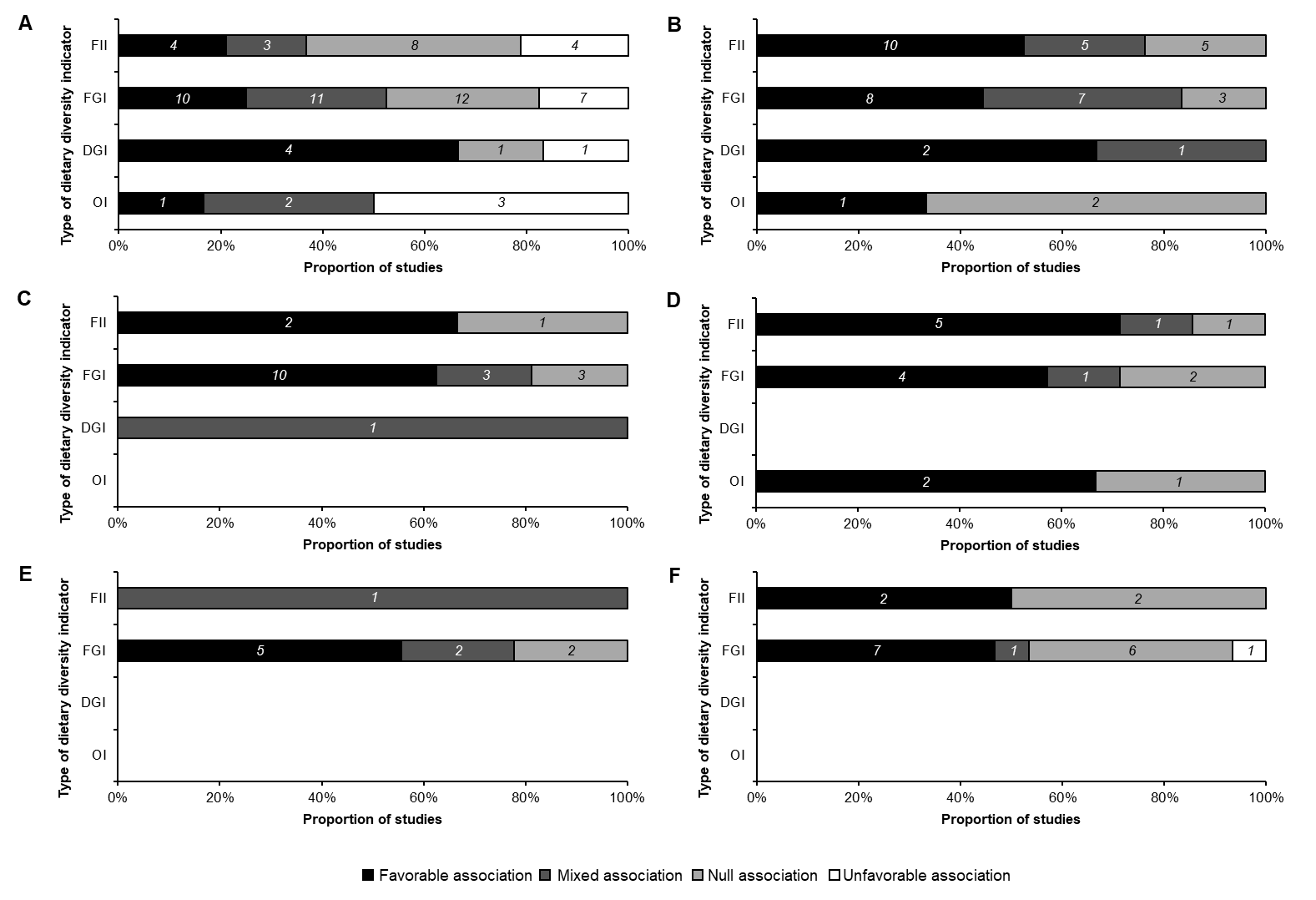


**Supplemental Figure 2.** Associations by type of dietary diversity indicators of studies investigating the associations between dietary diversity indicators and body composition (*n=*60, A), non-communicable diseases and intermediate biomarkers of health (*n=*41, B), biomarkers of nutritional status (*n=*19, C), mental health and cognitive functions (*n=*17, D), mortality (*n=*10, E), and other health outcomes (*n=*18, F). FII: Food Item-based Indicator. FGI: Food Group-based Indicator. DGI: Dietary Guidelines-based Indicator. OI: Other Indicator.

**Supplemental Table 1.** Summary of 50 studies evaluating the association between dietary diversity indicators and measures of dietary adequacy of the diet in participants aged 10 years and older^1^.

| Study (ref) | Year | Design | Country | Population | Dietary assessment | Dietary diversity indicator | Statistical methods | Associations - Nutrient adequacy | Associations- Nutrient moderation |
| --- | --- | --- | --- | --- | --- | --- | --- | --- | --- |
| Kant et al. (133) | 1993 | Long. | USA | Male and female adults (25-74 y.); *n=*10424 | One quantitative 24-hr recall; Interviewer-administered; 3D food models | FGI: DDS. Count of five food groups (dairy; meat; grain; fruit; and vegetable) consumed daily. Range from 0 to 5. Minimum reported amount for inclusion (15 g for solids or 30 g for liquids and mixed dishes when dairy, 30 g or 60 g when other groups). | Intakes of 3 macronutrients and energy; No energy adjustment | Positive association: DDS was positively correlated with energy and fiber intakes. |  |
| McCann et al. (73) | 1994 | Case. | USA | Male and female adults; *n=*856 | Quantitative FFQ to estimate average food use during the year preceding diagnosis or interview (128 food items); Self-administered; Portion size | FII: Total diet diversity. Count of foods consumed among six food groups (fruits; vegetables; grains; dairy; meats; nutrient non-dense foods) after having standardized for the number of food in each food group (z-score). Range from 0 to 114. Minimum reported amount for inclusion (once a month). | Intakes of 5 macro- and micronutrients and energy; No energy adjustment | Positive association: Total diversity was positively correlated with energy, fiber, carotene, vitamin C and calcium intakes. |  |
| Kant et al. (134) | 1995 | Long. | USA | Male and female adults (25-74 y.); *n=*10337 | One quantitative 24-h recall; Interviewer-administered; 3D food models | FGI: DDS. Count of five food groups (dairy; meat; grain; fruit; and vegetable) consumed daily. Range from 0 to 5. Minimum reported amount for inclusion (15 g for solids or 30 g for liquids and mixed dishes when dairy, 30 g or 60 g when other groups). | Percent Recommended Dietary Allowance of 5 micronutrient; No energy adjustment | Positive association: With increasing DDS, the mean percentage of recommended dietary allowance increased for riboflavin, thiamin, vitamin B6, vitamin C and calcium. |  |
| Hsu-Hage et al. (39) | 1996 | Cross. | Australia | Male and female adults (≥25 y.); *n=*545 | Assumption of a FFQ to estimate average food use during the last year | FII: Food variety. Count of unique foods consumed over the year. | Intakes of 17 macro- and micronutrients and energy; Percentage of individuals meeting the recommended Dietary Allowance of 10 macro- and micronutrients; No energy adjustment | Positive association: Adults with low food variety were at 2-fold risk for having an essential nutrient intake level fall below two third of RNI |  |
| Schuette et al. (135) | 1996 | Cross. | USA | Male and female adults (18-24 y.); *n=*2489 | One day quantitative food record (one typical weekday); Self-administered; Instructions to estimate serving sizes | FGI: Food group score V1. Count of five food groups (grains; fruits; vegetables; dairy; and meat) consumed daily. Range from 0 to 5. Minimum reported amount for inclusion (one serving); DGI: Food group score V2. Count of five food groups (grains; fruits; vegetables; dairy; and meat) consumed daily. Range from 0 to 5. Minimum reported amount for inclusion (6 servings of bread, cereal, rice and pasta, 3 servings of vegetables, 3 servings of fruits, 2 servings of milk, yogurt and cheese, 2 servings of meat, poultry, fish, dry beans, eggs and nuts based on the recommendations from the Food Guide Pyramid) | Mean adequacy ratio based on 5 micronutrients; No energy adjustment | Positive association: The two versions of the food group score had equally high sensitivity in identifying nutritionally inadequate diet (Mean adequacy ratio < 75). |  |
| Song et al. (136) | 1996 | Cross. | USA | Male and female adults (18-24 y.); *n=*2489 | One day quantitative food record (one typical weekday); Self-administered; Instructions to estimate serving sizes | DGI: Food group score. Count of five food groups (grains; fruits; vegetables; dairy; and meat) consumed daily. Range from 0 to 5. Minimum reported amount for inclusion (6 servings of bread, cereal, rice and pasta, 3 servings of vegetables, 3 servings of fruits, 2 servings of milk, yogurt and cheese, 2 servings of meat, poultry, fish, dry beans, eggs and nuts based on the recommendations from the Food Guide Pyramid) | Intakes of 19 macro- and micronutrients and energy; Mean adequacy ratio based on 5 micronutrients; No energy adjustment | Positive association: Diet with a food group score 0-4 vs. 5 differed significantly in distribution of mean adequacy ratio scores (higher proportion of diet nutritionally adequate when higher food group score). | Positive association: Positive correlations between food group score and higher intakes of fat and added sugars |
| Drewnowski et al. (137) | 1997 | Cross. | USA | Male and female adults (20-30 and 60-75 y.); *n=*48 | One 24-hr recall (Interviewer-administered) followed by 14 consecutive days of dietary record (Self-administered); Measuring cups, spoons and glasses | FII: DVS. Count of different food items consumed over 15 days. | Intakes of 10 macro- and micronutrients and energy; A five-point Diet Quality Index; No energy adjustment | Null association: DVS was positively correlated with vitamin C intakes, but not with dietary fiber and vitamin A intakes, nor with DQI-I. | Negative association: Higher DVS was correlated with lower sodium, sugar and SFA intakes. |
| Kant et al. (36) | 1997 | Cross. | USA | Male and female adults (≥18 y.); *n=*10799 | Quantitative FFQ to estimate average food use during the last year (68 food items); Interviewer-administered; Portion sizes (small, medium and large) | FII:   - DVS. Count of food items consumed among the nutrient-dense category. Range from 0 to 54. Minimum reported amount for inclusion (once a week). - DVSR. Count of recommended food items consumed among the nutrient-dense category. Range from 0 to 27. Minimum reported amount for inclusion (once a week). | Intakes of 4 macro- and micronutrients and energy; No energy adjustment | Positive association: DVS and DVSR were positively correlated with energy, vitamin C, carotenoid and calcium intakes. | Mixed association: DVSR was inversely correlated with percent energy from fat while DVS was not correlated. |
| Slattery et al. (41) | 1997 | Case. | USA | Male and female adults (30-79 y.); *n=*4403 | Diet history questionnaire with a referent period of a year (800 food items, 69 food groups); Interviewer-administered; Food models and plastic cups and spoons | FII: Total diet diversity. Count of different food items reported, excluding non-nutrient contributing food items such as coffee, tea, and water. | Intakes of 8 macro- and micronutrients and energy; No energy adjustment | Positive association: Total diversity was positively correlated with energy, fat, protein, carbohydrates, dietary fiber, carotene, vitamin C, folates and calcium intakes. |  |
| Kant et al. (138) | 1999 | Cross. | USA | Male and female adults (≥19 y.); *n=*7841 | One 24-hr recall (Interviewer-administered) followed by 2-day food records (Self-administered) | FII: OVS. Count of unique foods consumed among the five major food groups over 3 days. FGI: DDS. Count of five food groups (dairy; meat; grain; fruit; and vegetable) consumed over 3 days. Range from 0 to 5. | Intakes of 9 macro- and micronutrients and energy; Nutrient Adequacy Score (NAS100) is the number of nutrients consumed at least at the level of 100% of the RDA for 11 nutrients ; No energy adjustment | Positive association: OVS and DDS were positively correlated with energy and nutrient intakes, and with NAS100 (r=0.66 and r=0.52, respectively) |  |
| Löwik et al. (139) | 1999 | Cross. | Netherlands | Male and female adults (18-60 y.); *n=*various | Two days food record; Self-administered; | FII: Food variety. Count of different foods consumed during the two days. Range from 0 to 43. | Intakes of 14 macro- and micronutrients and energy; Energy adjustment | Null association: Higher food variety was associated with higher energy and nutrient intakes. After energy adjustment, there was no longer association. | Null association: Higher food variety was associated with higher energy and nutrient intakes. After energy adjustment, there was no longer association. |
| Ogle et al. (140) | 2001 | Cross. | Vietnam | Female adults (19-60 y.); *n=*217 | Quantitative FFQ to estimate average food use during the last week (12 food groups); Interviewer-administered; Photos of foods and dishes | FII: FVS. Count of different food items consumed during the week FGI: DDS. Count of twelve food groups (cereals; starchy roots; green leafy vegetables; other vegetables; fish, seafood; meat; eggs; nuts, legumes; fruits, juice; oils, fats; sauces; beverages, biscuits, sweets) consumed during the week. | Intakes of 12 macro- and micronutrients and energy; Nutrient adequacy ratio for 11 macro- and micronutrients and energy; Energy adjustment | Positive association: FVS was positively associated with energy and most of nutrient intakes. Having DDS ≥ was positively associated with higher adequacy ratios for energy, protein, niacin, vitamin C and zinc. |  |
| Bernstein et al. (27) | 2002 | Cross. | USA | Male and female adults (72-98 y.); *n=*98 | Three consecutive days food records; Interviewer-administered; Weighted | FII: Dietary variety score. Count of different foods consumed during 3 days. | Intakes of 18 macro- and micronutrients and energy; Energy adjustment | Null association: Higher dietary variety was significantly associated with many of the nutrients. After energy adjustment, only vitamin E and copper remained associated with dietary variety score. |  |
| Torheim et al. (141) | 2003 | Cross. | Mali | Male and female adults (15-59 y.); *n=*75 in Study A and 70 in Study B | Quantitative FFQ to estimate average food use during the last week (69 food items in study A, 164 food items in study B); Interviewer-administered; Standard recipes Two-day dietary record; Interviewer-administered; Weighed | FII: FVS. Count of different food items consumed during the diet-recording period. Minimum reported amount for inclusion (0.1 g). FGI: DDS. Count of ten food groups (cereals; legumes; oil/sugar; fruit; vegetables; meat; milk; fish; egg; and green leaves) consumed during the diet-recording period. Minimum reported amount for inclusion (0.1 g). | Nutrient adequacy ratio for 9 macro- and micronutrients and energy; Mean adequacy ratio based on 9 macro- and micronutrients and energy; No energy adjustment | Positive association: FVS and FGDS were positively correlated with most of the nutrient adequacy ratios and the mean adequacy ratio (r ranging from 0.24 to 0.53 according the study site and dietary assessment method). |  |
| Foote et al. (142) | 2004 | Cross. | USA | Male and female adults (≥19 y.); *n=*9769 | One quantitative 24-hr recall; Interviewer-administered | FGI: Total variety. Count of five food groups (grains; fruits; vegetables; dairy; and meat/protein) consumed daily (sub-component of the HIE). Range from 0 to 5. Minimum reported amount for inclusion (half-serving). | Mean probability of adequacy based on 15 micronutrients; Energy adjustment | Positive association: Total variety was positively correlated with mean probability of adequacy (r=0.68). Energy adjustment partially decreased the correlation (r=0.44 and r=0.46 for men and women). |  |
| Mirmiran et al. (16) | 2004 | Cross. | Iran | Male and female adolescents (10-18 y.); *n=*304 | Two non-consecutive quantitative 24-hr recalls within a 10-day period; Interviewer-administered; Household portions | FGI: DDS. Count of food items consumed among each of the five main food groups (grain, bread; vegetables; fruits; meats; dairy) with a maximal score up to 2. Sum of the scores from the five main food groups. Range from 0 to 10. Minimum reported amount for inclusion (half-serving over two days). | Intakes of 7 macronutrients and energy; Nutrient adequacy ratio for 12 macro- and micronutrients and energy; Mean adequacy ratio based on 12 macro- and micronutrients; No energy adjustment | Positive association: DDS was positively correlated with most of the nutrient adequacy ratios and the mean adequacy ratio (r=0.42). |  |
| Thiele et al. (143) | 2004 | Cross. | Germany | Male and female adults (18-79 y.); *n=*4030 | Dietary history questionnaire that evaluate eating habits over the past four weeks; Interviewer-administered; | FII: Food diversity. Count of different foods consumed. Range from 0 to 132. | Deficient index (13 vitamins, 12 minerals, proteins, carbohydrates, two essential fatty acids and dietary fiber) and Excess index (fat, cholesterol, the ratio of saturated to unsaturated fatty acids, sugar, alcohol and sodium); No energy adjustment | Positive association: Food diversity was positively associated deficient index. | Null association: Food diversity was not associated with the excess index. |
| Torheim et al. (144) | 2004 | Cross. | Mali | Male and female adults (15-45 y.); *n=*502 | Quantitative FFQ to estimate average food use during the last week (104 food items); Self-administered | FII: FVS. Count of food items consumed during the week. Range from 0 to 76.  FGI: DDS. Count of ten food groups (cereals; legumes; oil/sugar; fruit; vegetables; meat; milk; fish; egg; and green leaves) consumed during the week. Range from 0 to 10. | Mean adequacy ratio based on 9 macro- and micronutrients and energy; No energy adjustment | Positive association: FVS and DDS were positively correlated with the mean adequacy ratio (r=0.34 and r=0.30, respectively). |  |
| Azadbakht et al. (24) | 2005 | Cross. | Iran | Male and female adults (18-74 y.); *n=*581 | Semi-quantitative FFQ to estimate average food use during the last year (168 food items); Interviewer-administered; Standard serving size | FGI: DDS. Count of food items consumed among each of the five main food groups (grain, bread; vegetables; fruits; meats; dairy) with a maximal score up to 2. Sum of the scores from the five main food groups. Range from 0 to 10. Minimum reported amount for inclusion (half-serving per day). | Intakes of 7 macro- and micronutrients and energy; Energy adjustment | Positive association: DDS was positively correlated with fiber, calcium and vitamin C intakes. |  |
| Azadbakht et al. (145) | 2005 | Cross. | Iran | Male adults (≥18 y.); *n=*295 | Two non-consecutive quantitative 24-hr recalls within a 10-day period (only weekdays); Interviewer-administered; Household portions | FGI: Total variety score. Count of food items consumed among each of the five main food groups (grain, bread; vegetables; fruits; meats; dairy) with a maximal score up to 2. Sum of the scores from the five main food groups. Range from 0 to 10. Minimum reported amount for inclusion (half-serving over two days). | Probability of adequacy for 16 macro- and micronutrients and energy; Mean probability of adequacy based on 14 macro- and micronutrients; Energy adjustment | Positive association: Total variety score was positively correlated with mean probability of adequacy (r=0.65). Energy adjustment partially decreased the correlation (r=0.48). |  |
| Kant et al. (15) | 2005 | Cross. | USA | Male and female adults (≥20 y.); *n=*8719 | One quantitative 24-hr recall; Interviewer-administered; Abstract food models, special charts, measuring cups, and rulers | FII: FRS. Count of foods considered to be recommended consumed (low fat dairy; lean meats, poultry, fish, and alternatives; low fat whole grains such as whole wheat breads and cereals; all fruits and juices; all vegetables that were not fried, pickled, or creamed). Minimum reported amount for inclusion (15 g for non-beverages and 30 g for beverages).  FGI: DDS-R. Count of five food groups (dairy; meat; grain; fruit; and vegetable) consumed daily. Range from 0 to 5. Minimum reported amount for inclusion (15 g for non-beverages and 30 g for beverages). | Intakes of 15 macro- and micronutrients and energy; No energy adjustment | Positive association: Correlations of DDS-R and RFS with the micronutrients were positive and of a comparable magnitude. |  |
| Roberts et al. (13) | 2005 | Cross. | USA | Male and female adults (21-90 y.); *n=*1174 | Two non-consecutive quantitative 24-hr recall; Interviewer-administered | FII:   - Micronutrient-dense variety. Count of unique food and caloric beverage items consumed from important sources of protein or micronutrients. - Energy-dense variety. Count of unique food and caloric beverage items consumed from foods high in energy density. | Percent Estimated Average Requirement of 14 micronutrients and mean percentage of estimated average requirements for all 14 micronutrients; No energy adjustment | Mixed association: Micronutrient-dense variety was positively correlated with mean micronutrient intakes in both younger and older adults. Energy-dense variety was not correlated with mean micronutrient intakes in younger adults but was negatively correlated with mean micronutrient intakes in older adults. |  |
| Azadbakht et al. (50) | 2006 | Cross. | Iran | Male and female adults (≥18 y.); *n=*581 | Semi-quantitative FFQ to estimate average food use during the last year (168 food items); Interviewer-administered; Standard serving size | FGI: DDS. Count of food items consumed among each of the five main food groups (grain, bread; vegetables; fruits; meats; dairy) with a maximal score up to 2. Sum of the scores from the five main food groups. Range from 0 to 10. Minimum reported amount for inclusion (half-serving per day). | Intakes of 7 macro- and micronutrients and energy; Energy adjustment | Positive association: DDS was positively correlated with fiber, calcium and vitamin C intakes. |  |
| Mirmiran et al. (146) | 2006 | Cross. | Iran | Female adults (18-80 y.); *n=*286 | Two non-consecutive quantitative 24-hr recalls within a 10-day period (randomly selected weekdays); Interviewer-administered; Household portions | FGI: DDS. Count of food items consumed among each of the five main food groups (grain, bread; vegetables; fruits; meats; dairy) with a maximal score up to 2. Sum of the scores from the five main food groups. Range from 0 to 10. Minimum reported amount for inclusion (half-serving over two days). | Mean probability of adequacy based on 14 macro- and micronutrients; Energy adjustment | Positive association: DDS was positively correlated with mean probability of adequacy (r=0.60). Energy adjustment partially decreased the correlation (r=0.43). |  |
| Murphy et al. (147) | 2006 | Cross. | USA | Male and female adults (≥19 y.); *n=*9761 | One quantitative 24-hr recall; Interviewer-administered | FII:   - Food code variety. Count of different USDA food codes consumed daily. - Commodity variety. Count of different food items consumed daily. Minimum reported amount for inclusion (half-serving).   FGI:   - FGP5. Count of five food groups (grains, fruits, vegetables, dairy and meat) consumed daily. Range from 0 to 5. Minimum reported amount for inclusion (half-serving). - FGP22. Count of twenty-two food groups consumed daily. Range from 0 to 22. Minimum reported amount for inclusion (half-serving). | Intakes of 3 nutrients (added sugars, SFA, sodium); Mean probability of adequacy based on 15 micronutrients; Energy adjustment | Positive association: Energy-adjusted correlations of the four DDIs with mean probability of adequacy were positive (r from 0.22 to 0.46) | Negative association: Energy-adjusted correlations of the four DDIs with added sugars and saturated fat intakes were negative. |
| Ponce et al. (54) | 2006 | Cross. | Mexico | Male adults (35-65 y.); *n=*325 | Two non-consecutive quantitative 24-hr recalls within a 2 weeks period; Interviewer-administered; Cups, spoons, plates, and glasses | FGI: Dietary diversity. Count of twenty-four food groups consumed over two days. Range from 0 to 24 (analyzed in quartiles) | Micronutrient adequacy score computed based on 75% of the U. S. recommended dietary allowances for the 13 micronutrients; No energy adjustment | Positive association: Dietary diversity was positively correlated with micronutrient adequacy score (r=0.335). | Positive association: Dietary diversity was positively correlated with the percentage of energy intake from total fat, saturated fat and cholesterol intake. |
| Drescher et al. (1) | 2007 | Cross. | Germany | Male and female adults; *n=*4030 | Validated computerized dietary history method about the diet of the preceding month; Interviewer-administered | FII: Count Index. Count of different foods consumed.  DGI: HFD Index. Represents the number of different species consumed and how evenly the amounts consumed are distributed. Range from 0 to 1. OI: Berry Index. Multiplication of the Berry Index by the health value of the diet (which is the sum of volume share of each food multiplied by a health factor based on the German Nutrition Society). Range from 0 to 1. | Nutrient adequacy ratio for 30 macro- and micronutrients; Energy adjustment | Positive association: HFD Index, Berry Index and Count Index were positively correlated with most of the nutrient adequacy ratios (except vitamin B12). | Mixed association: HFD Index was positively correlated with the adequacy ratio of saturated to unsaturated fatty acids while Berry Index and Count Index were not. |
| Oldewage-Theron et al. (148) | 2008 | Cross. | South Africa | Male and female adults (≥60 y.); *n=*149 | Quantitative FFQ to estimate average food use during the past week; Interviewer-administered | FII: FVS. Count of food items consumed during the week.  FGI: FGDS. Count of nine food groups (cereals, roots and tubers; dairy; eggs; fats and oils; legumes and nuts; other vegetables; other fruit; vitamin A-rich fruit and vegetables; and flesh product) consumed during the week. Range from 0 to 9. | Nutrient adequacy ratio for 24 macro- and micronutrients and energy; Mean adequacy ratio based on 21 micronutrients; No energy adjustment | Positive association: FVS and FGDS were positively correlated with most of the nutrient adequacy ratios and the mean adequacy ratio (non-significant, only trend) |  |
| Roche et al. (149) | 2008 | Cross. | Peru | Female adults (15-49 y.); *n=*49 | Two non-consecutive quantitative 24-hr recalls (separation of 3-4 days); Interviewer-administered; Foods with known weights, cups, plates and bowls | FII: TFDS. Count of unique local/traditional foods consumed during the two days. | Intakes of 13 macro- and micronutrients and energy; Energy adjustment | Positive association: TFDS was positively correlated with most of nutrient intakes (from 0.16 to 0.66). |  |
| Gregory et al. (14) | 2009 | Cross. | Guatemala | Male and female adults; *n=*1220 | FFQ to estimate average food use during the last 3 months (52 food items); Interviewer-administered | FII:   - RFS. Count of recommended food items consumed. Range from 0 to 16. Minimum reported amount for inclusion (once a week). - NRFS. Count of not recommended food items consumed. Range from 0 to 15. Minimum reported amount for inclusion (once a week).   FVS. Count of any unique food consumed (excluding alcoholic or sweetened beverages or condiments such as jam, honey, mayonnaise or butter). Range from 0 to 39. Minimum reported amount for inclusion (once a week). | DQI-I; No energy adjustment | Mixed association: RFS and FVS were positively correlated associated with DQI-I but not NRFS. |  |
| Arimond et al. (150) | 2010 | Cross. | Burkina Faso, Mali, Mozambique, Bangladesh, and the Philippines | Female adults (15-49 y.) including pregnant and lactating women; *n=*3146 | Two quantitative 24-hr recalls (1st : minimum *n=*100, 2nd : minimum *n=*40); Interviewer-administered | FGI: Various versions of FGI. Count of six, nine, thirteen or twenty-one food groups consumed daily. Range from 0 to 6, 9, 13, or 21. Minimum reported amount for inclusion (1 g or 15 g). | Mean probability of adequacy based on 11 micronutrients; Energy adjustment | Positive association: The various versions of FGI were positively correlated with mean probability of adequacy (r from 0.21 to 0.53). Energy adjustment partially decreased the correlation (r from 0.12 to 0.46). |  |
| Vandevijvere et al. (40) | 2010 | Cross. | Belgium | Male and female adolescents (≥15 y.); *n=*3083 | Two non-consecutive 24-hr recalls within a 2 to 8-weeks period; Interviewer-administered; Picture book | FGI: Total dietary diversity score. Count of ten food groups (water, coffee, tea and broth; bread and cereals; grains and potatoes; vegetables; fruits; dairy products; cheese; meat, fish, eggs, legumes, nuts and meat substitutes; spreadable fats; and energy-dense, nutrient-poor foods) consumed daily and average over the 2 days. Range from 0 to 10. Minimum reported amount for inclusion (one serving). | Overall scores for dietary adequacy, moderation and balance (based on food group consumption); Energy adjustment | Positive association: Total dietary diversity was positively associated with dietary adequacy, moderation and balance. | Negative association: Total dietary diversity was positively associated with dietary adequacy, moderation and balance. |
| Oldewage-Theron et al. (151) | 2011 | Cross. | South Africa | Female adults (31-50 y.); *n=*426 | Quantitative FFQ to estimate average food use during the past week; Interviewer-administered | FII: FVS. Count of food items consumed during the week. FGI: FGDS. Count of nine food groups (cereals, roots and tubers; dairy; eggs; fats and oils; legumes and nuts; other vegetables; other fruit; vitamin A-rich fruit and vegetables; and flesh product) consumed during the week. Range from 0 to 9. | Nutrient adequacy ratio for 23 macro- and micronutrients and energy; Mean adequacy ratio based on 21 micronutrients; No energy adjustment | Positive association: FVS and FGDS were positively correlated with most of the nutrient adequacy ratios and the mean adequacy ratio (r=0.22 and r=0.24, respectively). |  |
| Rathnayake et al. (152) | 2012 | Cross. | Sri Lanka | Male and female adults (≥60 y.); *n=*200 | One quantitative 24-hr recall; Interviewer-administered; Cups, table spoons, coconut spoons, size of box of matches, common household measures and photographs of servings | FII: FVS. Count of unique food items consumed daily.  FGI:   - DDS. Count of six food groups (cereals, roots; vegetables; fruits; legumes, lentils; meat, fish, egg; and milk, dairy products) consumed daily. Range from 0 to 6. - DDS-half-serving. Count of the same six food groups consumed daily. Range from 0 to 6. Minimum reported amount for inclusion (half-serving).   DGI: DSS. Weighted sum of the same six food groups consumed daily. Range from 0 to 20. Minimum reported amount for inclusion (4 servings of cereals, roots (4 points), 2 servings of vegetables (4 points), 2 servings of fruits (4 points), 2 servings of milk, dairy products (4 points), 1 serving of legumes, lentils (2 points), 1 serving of meat ,fish, egg (2 points) based on the recommendations from dietary guidelines of Sri Lanka). | Nutrient adequacy ratio for 11 macro- and micronutrients and energy; Mean adequacy ratio based on 11 macro- and micronutrients and energy; No energy adjustment | Positive association: FVS, DDS, DDS-half serving and DSS were positively correlated with most of the nutrient adequacy ratios and the mean adequacy ratio (r=0.45, r=0;48, r=0.50 and r=0.58, respectively). |  |
| Arsenault et al. (153) | 2013 | Cross. | Bangladesh | Lactating and non-lactating female adults; *n=*478 | Two non-consecutive quantitative days of dietary information (12-h weighed food records and 12-hr recall) during 1 week; Interviewer-administered; 2D plates and local cups and utensils | FGI: MDDS and MDDS-R. Count of nine food groups (starchy staples; legumes and nuts; dairy; organ meats; eggs; flesh foods; vitamin A-rich dark green leafy vegetables; other vitamin A-rich fruits and vegetables; and other fruits and vegetables) consumed daily and average over the 2 days. Range from 0 to 9. Minimum reported amount for inclusion (1 g for MDDS and 15 g for MDDS-R). | Mean probability of adequacy based on 11 micronutrients; Energy adjustment | Positive association: MDDS and MDDS-R were positively correlated with mean probability of adequacy (r=0.25 and r=0.32, respectively). Energy adjustment partially decreased the correlation (r= 0.18). |  |
| Azadbakht et al. (17) | 2014 | Cross. | Iran | Female adolescents (11-13 y.); *n=*265 | Semi-quantitative FFQ to estimate average food use during the last year (53 food items); Self-administered | FGI: DDS. Count of food items consumed among each of the five main food groups (grain, bread; vegetables; fruits; meats; dairy) with a maximal score up to 2. Sum of the scores from the five main food groups. Range from 0 to 10. | Intakes of 18 macro- and micronutrients and energy; Energy adjustment | Positive association: DDS was positively associated with most of nutrient intakes (vitamin A, vitamin D, vitamin B6, folate, zinc, potassium, calcium, magnesium, vitamin B12 and protein). |  |
| Vadiveloo et al. (154) | 2014 | Cross. | USA | Male and female adults (≥20 y.); *n=*7470 | Two non-consecutive quantitative 24-hr recalls within a 2-weeks period; Interviewer-administered; Food models and regular household measures | DGI: US HFD index. Multiplication of the Berry Index by the health value of the diet (which is the sumof volume share of each food multiplied by a health factor based on the US Dietary Guidelines). Range from 0 to 1. | Probability of nutrient adequacy for 15 micronutrients; Mean probability of adequacy based on 15 micronutrients; DASH; Energy adjustment | Positive association: US HFD index was positively correlated with mean probability of adequacy (r=0.41) and DASH (r=0.35). Energy adjustment did not attenuate correlations with mean probability of adequacy. | Negative association: US HFD index was inversely correlated with sodium, total fat and cholesterol intakes. |
| Henjum et al. (155) | 2015 | Cross. | Nepal | Lactating female adults (17-44 y.); *n=*500 | Three non-consecutive quantitative 24-hr recalls (including weekends); Interviewer-administered; Electronic scales and common household utensils | FGI: DDS. Count of eight food groups (starchy staples; dark green leafy vegetables; other vitamin A-rich fruits and vegetables; other fruits and vegetables; meat and fish; eggs; legumes, nuts and seeds; and milk and milk products) consumed daily and average over the 3 days. Range from 0 to 8. Minimum reported amount for inclusion (15 g) | Mean probability of adequacy based on 11 micronutrients; Energy adjustment | Positive association: DDS was positively correlated with mean probability of adequacy (r=0.30). Energy adjustment partially decreased the correlation but remained significant. |  |
| Masset et al. (85) | 2015 | Long. | UK | Male and female adults (35-55 y.); *n=*7251 | Quantitative FFQ to estimate average food use during the last year (127 food items); Self-administered; Common unit or portion size | FII:   - FVS. Count of food items reported to be consumed. Minimum reported amount for inclusion (once a week). - RFV(Ofcom) and RFV(SAIN,LIM). Count of foods identified as ‘healthy’ by the Ofcom and SAIN,LIM models. Minimum reported amount for inclusion (once a week). - NRFV(Ofcom) and NRFV(SAIN,LIM). Count of foods identified as ‘less healthy’ aby the Ofcom and SAIN,LIM models (the higher, the worst). Minimum reported amount for inclusion (once a week). | Mean adequacy ratio based on 19 micronutrients; Mean excess ratio based on 3 nutrients; No energy adjustment | Positive association: All the scores were positively associated with mean adequacy ratio. | Mixed association: All the scores (except RFV(SAIN,LIM)) were positively associated with mean excess ratio. |
| Otto et al. (2) | 2015 | Long. | USA | Male and female adults (45-84 y.); *n=*5160 | Quantitative FFQ to estimate average food use during the last year (120 food items); Self-administered; Portion sizes (small, medium and large) | FII: Food count. Count of food items consumed. Range from 0 to 120. Minimum reported amount for inclusion (once a week). OI:   - Evenness. Represents the number of different foods consumed and how evenly the amounts consumed are distributed. Range from 0 to 1. Minimum reported amount for inclusion (once a week). - Dissimilarity. Average distance for pairwise comparisons of all food items consumed by each participant using 12 different food attributes based on likely evidence for associations on cardio-metabolic health. Range from 0 to 1. Minimum reported amount for inclusion (once a week). | Diet quality indicators (DASH, aHIE and an a priori dietary pattern score previously developed in the Coronary Artery Risk Development in Young Adults Study); No energy adjustment | Mixed association: Food count was positively correlated with diet quality indicators (r from 0.17 to 0.20), Evenness was not correlated with diet quality indicators (r from 0.03 to 0.06) and Dissimilarity was negatively correlated with diet quality indicators (r from -0.34 to -0.37) | Mixed association: Food count was positively correlated with diet quality indicators (r from 0.17 to 0.20), Evenness was not correlated with diet quality indicators (r from 0.03 to 0.06) and Dissimilarity was negatively correlated with diet quality indicators (r from -0.34 to -0.37) |
| Bianchi et al. (156) | 2016 | Cross. | France | Male and female adults (18-74 y.); *n=*1330 | Three non-consecutive quantitative 24-hr recalls within a 2-weeks period; Interviewer-administered | FGI: Div-S. Count of subgroups consumed divided by the number of subgroups among the seven main food group (cereals; fruit and nuts; vegetables; fats; dairy products; sugar products; meat, fish, and eggs). Mean of these seven ratios multiplied by 100. Score from 0 to 100. | The PANDiet is a 100-point score that associations from the mean of 2 subscores: the Adequacy subscore (Adeq-S) and the Moderation subscore (Mod-S); No energy adjustment | Positive association: Div-S was positively associated with adequacy score (B=0.50). | Positive association: Div-S was negatively associated with moderation score (B=-0.17). |
| Shiraseb et al. (132) | 2016 | Cross. | Iran | Female adults (20-50 y.); *n=*400 | One quantitative 24-hr recall; Interviewer-administered; Common household utensils | FGI: DDS. Count of nine food groups (starchy staples and white roots; vitamin A-rich fruits and vegetables; dark green leafy vegetables; other fruits and vegetables; organ meat; meat and fish; eggs; legumes, nuts and seeds; and milk and milk products) consumed daily. Range from 0 to 9. Minimum reported amount for inclusion (half-serving). | Intakes of 15 macro- and micronutrients; No energy adjustment | Positive association: DDS was positively correlated with intake of energy from carbohydrate, selenium, dietary fiber, iron, zinc and pyridoxine. |  |
| Tavakoli et al. (37) | 2016 | Cross. | Iran | Female adults (≥60 y.); *n=*292 | Two non-consecutive quantitative 24-hr recalls within a 1-wk period (weekdays); Interviewer-administered; Visual aids, food portion models and common household utensils | FGI: DDS. Count of food items consumed among each of the five main food groups (grain, bread; vegetables; fruits; meats; dairy) with a maximal score up to 2. Sum of the scores from the five main food groups. Range from 0 to 10. Minimum reported amount for inclusion (half-serving over two days). | Nutrient adequacy ratio for 12 macro- and micronutrients and energy; Mean adequacy ratio based on 12 macro- and micronutrients; No energy adjustment | Positive association: DDS was positively correlated with all of the nutrient adequacy ratios and the mean adequacy ratio (r=0.65). |  |
| Nithya et al. (63) | 2017 | Cross. | India | Male and female adolescents (≥13 y.); *n=*1645 | One quantitative 24-hr recall | FGI: Food score. Count of thirteen food groups (reference FAO 2013). Range from 1 to 13. | Nutrient adequacy ratio for 10 macro- and micronutrients and energy; Mean adequacy ratio based on 10 macro- and micronutrients and energy; No energy adjustment | Positive association: Food score was positively correlated with all the nutrient adequacy ratios and the mean adequacy ratio (r=0.44). |  |
| Otsuka et al. (5) | 2017 | Long. | Japan | Male and female adults (60-81 y.); *n=*570 | Three consecutive days quantitative food record (2 weekdays and 1 weekend day); Self-administered; Weighted | OI: QUANTIDD. Calculated by the proportion of foods that contribute to total energy (or amount) and the number of food groups among 17. Range from 0 to 1. | Intakes of 18 macro- and micronutrients and energy; No energy adjustment | Positive association: QUANTIDD was positively correlated with protein, calcium, magnesium, iron, zinc, coper, vitamin B groups and vitamin C intakes. |  |
| Poorrezaeian et al. (111) | 2017 | Cross. | Iran | Female adults (20-49 y.); *n=*360 | One quantitative 24-hr recall; Interviewer-administered | FGI: DDS. Count of nine food groups (cereals and white roots; milk and dairy products; vitamin A-rich vegetable and fruits; green leafy vegetables; other vegetable and fruits; meat, fish and seafood; organ meat; eggs; nut, seeds and legumes) consumed daily. Range from 0 to 9. Minimum reported amount for inclusion (half-serving). | Intakes of 23 macro- and micronutrients and energy; No energy adjustment | Positive association: DDS was positively correlated with energy and most of the nutrient intakes. | Positive association: DDS was positively correlated with SFA intakes. |
| Zhang et al. (57) | 2017 | Cross. | China | Male and female adults (≥18 y.); *n=*1105 | Three consecutive quantitative 24-hr recalls (including weekdays and one weekend day); Interviewer-administered | FGI: DDS. Count of nine food groups (cereals, tubers, and roots; vegetables; fruits; meat; beans nuts and seeds; eggs, fish and seafood; dairy; and oil) consumed over the 3 days. Range from 0 to 9. | Nutrient adequacy ratio for 15 macro- and micronutrients and energy; No energy adjustment | Positive association: DDS was positively correlated with most of the nutrient adequacy ratios. |  |
| Gholizadeh et al. (78) | 2018 | Cross. | Iran | Male and female adults (20-60 y.); *n=*300 | Semi-quantitative FFQ to estimate average food use during the last year (168 food items); Interviewer-administered; Standard serving size | FGI: DDS. Count of nine food groups (starch; milk; green leafy vegetables; vegetables and fruits rich in vitamin A; other fruits; meat; fish; legumes; oils and fats) consumed over the year. Range from 0 to 9. | Intakes of 7 macro- and micronutrients and energy; No energy adjustment | Positive association: DDS was positively correlated with energy, fiber, vitamin B2 and calcium intakes. |  |
| Lachat et al. (3) | 2018 | Cross. | DRCongo, Ecuador, Kenya, Sri Lanka, Vietnam | Female adults (15-49 y.); *n=*2188 | One quantitative 24-hr recall in all studies; Interviewer-administered | FII: Species richness. Count of different species consumed. OI:   - Simpson’s index of diversity. Represents the number of different species consumed and how evenly the amounts consumed are distributed. Range from 0 to 1. - Functional diversity. Reflects the diversity in nutrient composition of different species consumed. Range from 0 to 1. | Mean adequacy ratio based on 6 micronutrients; Energy adjustment | Positive association: Species richness, Simpson’s index of diversity and Functional diversity were all positively associated with mean adequacy ratio. Energy adjustment did not attenuate correlations |  |
| Nguyen et al. (157) | 2018 | Cross. | Bangladesh | Pregnant female adolescents and adults (13-43 y.); *n=*600 | One qualitative 24-hr recall (for DDI) and one quantitative 24-hr recall (for measure of nutritional adequacy); Interviewer-administered; Standard pots, plates, bowls, cups, spoons, and other common household utensils | FGI: WDDS-10. Count of ten food groups (starchy staple foods; beans and peas; nuts and seeds; dairy products; flesh foods; eggs; dark green vegetables; vitamin A–rich fruits and vegetables; other vegetables; and other fruits) consumed daily. Range from 0 to 10. | Mean probability of adequacy based on 11 micronutrients; Energy adjustment | Positive association: WDDS-10 was positively correlated with mean probability of adequacy. Energy adjustment partially decreased the correlation. |  |

1 Case., Case-control; Cross., Cross-sectional; Long., Longitudinal

**Supplemental Table 2.** Summary of 60 studies evaluating the association between dietary diversity indicators and body weight and body composition in participants aged 10 years and older^1^.

| Study (ref) | Year | Design | Country | Population | Dietary assessment | Dietary diversity indicator | Statistical methods | Associations |
| --- | --- | --- | --- | --- | --- | --- | --- | --- |
| Hsu-Hage et al. (39) | 1996 | Cross. | Australia | Male and female adults (≥25 y.); *n=*545 | Assumption of a FFQ to estimate average food use during the last year | FII: Food variety. Count of unique foods consumed over the year. | Pearson's correlation adjusted for age | Null association: Food variety was not correlated with BMI, abdominal circumference, hip circumference nor body fatness |
| Kant et al. (36) | 1997 | Cross. | USA | Male and female adults (≥18 y.); *n=*10799 | Quantitative FFQ to estimate average food use during the last year (68 food items); Interviewer-administered; Portion sizes (small, medium and large) | FII:   - DVS. Count of food items consumed among the nutrient-dense category. Range from 0 to 54. Minimum reported amount for inclusion (once a week). - DVSR. Count of recommended food items consumed among the nutrient-dense category. Range from 0 to 27. Minimum reported amount for inclusion (once a week). | Pearson’s correlation | Null association: Self-reported BMI was not correlated with DVS (r=0.006) and DVSR (r= -0.01) |
| Slattery et al. (41) | 1997 | Case. | USA | Male and female adults (30-79 y.); *n=*4403 | Diet history questionnaire with a referent period of a year (800 food items, 69 food groups); Interviewer-administered; Food models and plastic cups and spoons | FII: Total diet diversity. Count of different food items reported, excluding non-nutrient contributing food items such as coffee, tea, and water. | Multi-adjusted logistic regression | Null association: Diet diversity was not correlated with BMI. |
| McCrory et al. (10) | 1999 | Cross. | USA | Male and female adults (20-80 y.); *n=*71 | Quantitative FFQ to estimate average food use during the last 6 months; Interviewer-administered; Portion sizes (small, medium, or large) | OI: Variety ratio. Percentage of different food items consumed within vegetables over the last 6 months divided by the sum of percentages of different food items consumed within sweets, snacks, condiments, entrées, and carbohydrates over the last 6 months. | Multi-adjusted linear regression | Favorable association: Variety ratio was inversely associated body fatness (partial r=-0.39). |
| Bernstein et al. (27) | 2002 | Cross. | USA | Male and female adults (72-98 y.); *n=*98 | Three consecutive days food records; Interviewer-administered; Weighted | FII: Dietary variety score. Count of different foods consumed during 3 days. | Multi-adjusted linear regression | Mixed association: Dietary variety score significantly predicted BMI classification in women (B: 0.34) but not in men. |
| Yao et al. (56) | 2003 | Cross. | China | Male and female adults (35-49 y.); *n=*130 | Three days quantitative food record (2 weekdays and 1 weekend day); Interviewer-administered; Weighted whenever possible (else estimated) | FII: Dietary variety. Count of unique recipe ingredients (in home prepared dishes) consumed over 3 days. | Multi-adjusted logistic regression | Unfavorable association: Dietary variety was positively associated with percentage of body fat (partial r=0.186). |
| Mirmiran et al. (16) | 2004 | Cross. | Iran | Male and female adolescents (10-18 y.); *n=*304 | Two non-consecutive quantitative 24-hr recalls within a 10-day period; Interviewer-administered; Household portions | FGI: DDS. Count of food items consumed among each of the five main food groups (grain, bread; vegetables; fruits; meats; daity) with a maximal score up to 2. Sum of the scores from the five main food groups. Range from 0 to 10. Minimum reported amount for inclusion (half-serving over two days). | T-test | Favorable association: BMI was greater in adolescents with higher DDS (19.81 ± 4.07 vs 18.95 ± 3.30 kg/m²). |
| Sea et al. (8) | 2004 | Case. | Hong Kong SAR, China | Male and female adults (18-50 y.); *n=*120 | Quantitative FFQ to estimate average food use during the past week (259 food items); Interviewer-administered; Food replica, food containers and pictures of food portions | OI: Food variety ratio. Percentage of different food items consumed within snacks over the week divided by the sum of percentages of different food items consumed within grains and meats over the week. | Pearson's correlation adjusted for age and sex | Unfavorable association: Food variety ratio was positively correlated with obesity indices (r=0.58 for body fat, r=0.59 for waist circumference and r=0.52 for hip circumference). |
| Azadbakht et al. (24) | 2005 | Cross. | Iran | Male and female adults (18-74 y.); *n=*581 | Semi-quantitative FFQ to estimate average food use during the last year (168 food items); Interviewer-administered; Standard serving size | FGI: DDS. Count of food items consumed among each of the five main food groups (grain, bread; vegetables; fruits; meats; dairy) with a maximal score up to 2. Sum of the scores from the five main food groups. Range from 0 to 10. Minimum reported amount for inclusion (half-serving per day). | Multi-adjusted logistic regression | Mixed association: The prevalence of obesity was higher among adults in the upper category of DDS compared to lower one while higher DDS was associated with lower waist circumference. |
| Clausen et al. (42) | 2005 | Cross. | Botswana | Male and female adults (≥60 y.); *n=*372 | Qualitative FFQ to estimate average food use during an unknown period (21 food items); Interviewer-administered | FII: FVS. Sum of the frequency scores (0 to 7) of 16 selected food consumed. Range from 0 to 112. Minimum reported amount for inclusion (once a week). | T-test | Null association: FVS was not different across BMI groups. |
| Kant et al. (15) | 2005 | Cross. | USA | Male and female adults (≥20 y.); *n=*8719 | One quantitative 24-hr recall; Interviewer-administered; Abstract food models, special charts, measuring cups, and rulers | FII: FRS. Count of foods considered to be recommended consumed (low fat dairy; lean meats, poultry, fish, and alternatives; low fat whole grains such as whole wheat breads and cereals; all fruits and juices; all vegetables that were not fried, pickled, or creamed). Minimum reported amount for inclusion (15 g for non-beverages and 30 g for beverages).  FGI: DDS-R. Count of five food groups (dairy; meat; grain; fruit; and vegetable) consumed daily. Range from 0 to 5. Minimum reported amount for inclusion (15 g for non-beverages and 30 g for beverages). | Multi-adjusted linear regression | Favorable association: BMI was inversely associated with FRS (B:-0.18) and (B:-0.38). |
| Roberts et al. (13) | 2005 | Cross. | USA | Male and female adults (21-90 y.); *n=*1174 | Two non-consecutive quantitative 24-hr recall; Interviewer-administered | FII:   - Micronutrient-dense variety. Count of unique food and caloric beverage items consumed from important sources of protein or micronutrients. - Energy-dense variety. Count of unique food and caloric beverage items consumed from foods high in energy density. | Multi-adjusted linear regression | Unfavorable association: Energy-dense variety foods predicted BMI at all ages in multiple regression models controlling for confounding variables (R²=0.574). Micronutrient-dense variety also predicted BMI but to a lesser extent. |
| Savy et al. (59) | 2005 | Cross. | Burkina Faso | Female adults (15-45 y.); *n=*588 | One qualitative 24-hr recall; Interviewer-administered | FII: FVS. Count of food items consumed daily.  FGI: DDS. Count of fourteen food groups (cereals; roots, tubers; pulses and nuts; green leafy vegetables; other vegetables; fruits; sugar; meat, poultry, insects; eggs; fish, sea food; milk, dairy products; oils and fats; condiments; drinks and miscellaneous) consumed daily. Range from 0 to 14. | Multi-adjusted logistic regression | "Favorable association: Women were more likely to be underweight in the lowest tertile of FVS (OR: 1.9, 95%CI: 1.0, 3.7) and lower tertile of DDS (OR: 2.9, 95%CI: 1.5, 5.6). FVS and DDS were positively associated with BMI, MUAC and body fat percentage. Most of the association became non-significant when sociodemographic and economic characteristics were accounted for. |
| Shariff et al. (21) | 2005 | Cross. | Malaysia | Female adults (≥20 y.); *n=*200 | Quantitative FFQ to estimate average food use during the last month (58 food items); Interviewer-administered; Set of calibrated household measurements | FII: FVS. Count of unique foods consumed. Range from 0 to 58. Minimum reported amount for inclusion (2 times a week). | Univariate logistic regression | Favorable association: Higher FVS was favorable against overweight (OR: 0.9, 95%CI: 0.8, 0.9) and lower FVS was significantly associated with at-risk WC (OR: 0.9, 95%CI: 0.8, 0.9). |
| Azadbakht et al. (50) | 2006 | Cross. | Iran | Male and female adults (≥18 y.); *n=*581 | Semi-quantitative FFQ to estimate average food use during the last year (168 food items); Interviewer-administered; Standard serving size | FGI: DDS. Count of food items consumed among each of the five main food groups (grain, bread; vegetables; fruits; meats; dairy) with a maximal score up to 2. Sum of the scores from the five main food groups. Range from 0 to 10. Minimum reported amount for inclusion (half-serving per day). | Multi-adjusted logistic regression | Unfavorable association: Probability of being obese increased across DDS quartiles. |
| Ponce et al. (54) | 2006 | Cross. | Mexico | Male adults (35-65 y.); *n=*325 | Two non-consecutive quantitative 24-hr recalls within a 2 weeks period; Interviewer-administered; Cups, spoons, plates, and glasses | FGI: Dietary diversity. Count of twenty-four food groups consumed over two days. Range from 0 to 24. | ANOVA | Unfavorable association: DDS was higher in obese adults than adults with normal weight. |
| Savy et al. (60) | 2006 | Long. | Burkina Faso | Female adults (15-45 y.); *n=*450 and 400 | One qualitative 24-hr recall; Interviewer-administered | FGI: DDS. Count of nine food groups (cereals, roots, tubers; pulses, nuts; vitamin A–rich fruits, vegetables; other vegetables; other fruits; meat, poultry, fish; eggs; milk, dairy products; and oils, fats) consumed daily. Range from 0 to 9. | Multi-adjusted logistic regression | Favorable association: Less underweight women in the higher category of DDS than in other categories in both seasons. |
| Benefice et al. (12) | 2007 | Cross. | Bolivia | Female adults; *n=*195 | One 24 hr-recall combined with FFQ to estimate average food use during the last week; Interviewer-administered; | OI: Food diversity index. Count of seven food groups (fish; cereals; tubers; plantain; fruits; meat; and milk) consumed over a week taking into account weighting to confer more importance to foods of animal origin (weight = 4), then cereals (weight = 3), fruits (weight = 2), and tubers and plantain (weight = 1). Range from 0 to 19. | Multi-adjusted logistic regression | Unfavorable association: Women with higher FDI were more prone to overweight than other. |
| Savy et al. (62) | 2007 | Cross. | Burkina Faso | Female adults (15-45 y.); *n=*550 | Three consecutive qualitative 24-hr recall; Interviewer-administered | FGI: DDS. Count of nine food groups (cereals, roots, tubers; pulses, nuts; vitamin A–rich fruits, vegetables; other vegetables; other fruits; meat, poultry, fish; eggs; milk, dairy products; and oils, fats) consumed daily. Range from 0 to 9. | Multi-adjusted logistic regression | Mixed association: DDS was positively associated with being less likely to be undernourished (BMI < 18.5 kg/m²) when based on one 24hr-recall (OR: 4.6, 95%CI: 1.8, 11.8), but was not associated when based on three 24hr-recalls (OR: 2.4, 95%CI: 1.0, 5.8). |
| Savy et al. (64) | 2008 | Cross. | Burkina Faso | Female adults (20-59 y.); *n=*481 | One qualitative 24-hr recall; Interviewer-administered | FGI:   - DDS-9. Count of nine food groups (cereals, roots, tubers; pulses, nuts; vitamin A–rich fruits, vegetables; other vegetables; other fruits; meat, poultry, fish; eggs; milk, dairy products; and oils, fats) consumed daily. Range from 0 to 9. - DDS-22. Count of twenty-two food groups consumed daily. Range from 0 to 22. | Multi-adjusted linear and logistic regression | Null association: DDS-9 and DDS-22 were not associated with BMI, MUAC nor body fat percentage. |
| Woo et al. (7) | 2008 | Long. | Hong Kong SAR, China | Male and female adults (25-74 y.); *n=*343 | Quantitative FFQ to estimate average food use during the last week (266 food items); Self-administered; Photographs of servings | OI: Food variety ratio. Percentage of different food items consumed within snacks over the week divided by the sum of percentages of different food items consumed within grains and meats over the week. | Multi-adjusted logistic regression | Unfavorable association: Increased food variety ratio was associated with increased risk for developing overweight (OR: 1.36, 95%CI: 1.01, 1.82). |
| Kent et al. (28) | 2009 | Cross. | Australia | Male and female adults; *n=*1522 | Qualitative FFQ to estimate average food use during an unknown period (42 food items in 1976, 44 food items in 1986, unknown in 2005); Self-administered | FII: Food variety. Assumption of a Count of different food items consumed. | Multi-adjusted linear regression | Mixed association: Food variety was not associated with BMI (except for men in 1976 where it was negatively related to BMI (B: -0.18). |
| Kimura et al. (43) | 2009 | Cross. | China | Male and female adults (≥60 y.); *n=*240 | FFQ to estimate average food use during the last week (11 food items); Interviewer-administered | FGI: FDSK-11. Count of eleven food groups (grain; meat; fish and shellfish; eggs; milk; beans and bean products; potatoes; vegetables; seaweed; nuts; and fruits) consumed. Range from 0 to 11. | T-test | Null association: No association was found between FDSK-11 and BMI |
| Gregory et al. (14) | 2009 | Cross. | Guatemala | Male and female adults; *n=*1220 | FFQ to estimate average food use during the last 3 months (52 food items); Interviewer-administered | FII:   - RFS. Count of recommended food items consumed. Range from 0 to 16. Minimum reported amount for inclusion (once a week). - NRFS. Count of not recommended food items consumed. Range from 0 to 15. Minimum reported amount for inclusion (once a week).   FVS. Count of any unique food consumed (excluding alcoholic or sweetened beverages or condiments such as jam, honey, mayonnaise or butter). Range from 0 to 39. Minimum reported amount for inclusion (once a week). | Multi-adjusted linear regression | Null association: RFS, NRFS and FVS were not associated to BMI nor waist circumference. |
| Saibul et al. (44) | 2009 | Cross. | Malaysia | Female adults (18-55 y.); *n=*182 | Three quantitative 24-hr recall (2 weekdays and a weekend day); Interviewer-administered; Common household measurements | FII: FVS. Count of different foods consumed during 3 days. Range from 0 to 69. | Multi-adjusted logistic regression | Null association: FVS was not associated with overweight and obesity of mothers. |
| Haemamalar et al. (45) | 2010 | Cross. | Malaysia | Male and female adults (≥16 y.); *n=*57 | Qualitative FFQ to estimate average food use during an unknown period (37 food items); Interviewer-administered | FGI: DDS. Count of thirty-seven food groups consumed. Range from 0 to 37. Minimum reported amount for inclusion (twice a week). | Pearson’s correlation | Null association: DDS was not significantly correlated with BMI (r=0.02) nor waist circumference (r=0.08). |
| Jovanović et al. (46) | 2010 | Cross. | Croatia | Female adults (51-70 y.); *n=*124 | Quantitative FFQ to estimate average food use during an unknown period; Interviewer-administered | FGI: Dietary variety. Count of five food groups consumed (grains; fruits; vegetables; dairy; and meat/protein), taking into account the variety of food within the main food groups (sub-component of the HIE). Range from 0 to 10. | ANOVA | Null association: No significant difference in dietary variety between BMI groups. |
| Vandevijvere et al. (40) | 2010 | Cross. | Belgium | Male and female adolescents (≥15 y.); *n=*3083 | Two non-consecutive 24-hr recalls within a 2 to 8-weeks period; Interviewer-administered; Picture book | FGI: Total dietary diversity score. Count of ten food groups (water, coffee, tea and broth; bread and cereals; grains and potatoes; vegetables; fruits; dairy products; cheese; meat, fish, eggs, legumes, nuts and meat substitutes; spreadable fats; and energy-dense, nutrient-poor foods) consumed daily and average over the 2 days. Range from 0 to 10. Minimum reported amount for inclusion (one serving). | Multi-adjusted logistic regression | Null association: Total dietary diversity score was not associated with BMI |
| Azadbakht et al. (22) | 2011 | Cross. | Iran | Female adults (18-28 y.); *n=*289 | Semi-quantitative FFQ to estimate average food use during the last year (168 food items); Interviewer-administered; Standard serving size | FGI: DDS. Count of food items consumed among each of the five main food groups (grain, bread; vegetables; fruits; meats; dairy) with a maximal score up to 2. Sum of the scores from the five main food groups. Range from 0 to 10. Minimum reported amount for inclusion (half-serving per day). | Multi-adjusted logistic regression | Favorable association: Probability of being overweight and obesity (OR:0.41, 0.31 and 0.21) and having abdominal adiposity (OR: 0.55, 0.36 and 0.21), decreased with quartiles of DDS. |
| Lee et al. (47) | 2011 | Long. | Taiwan | Male and female adults (≥65 y.); *n=*1743 | One quantitative 24-hr recall; Interviewer-administered | FGI: DDS. Count of six food groups (dairy; eggs, legumes, fish, meat; grain; fruit; vegetable; and oil, fat) consumed daily. Range from 0 to 6. Minimum reported amount for inclusion (half-serving). | Multi-adjusted Cox's proportional-hazards regression | Null association: DDS was not associated with BMI. |
| Mayega et al. (29) | 2012 | Cross. | Uganda | Male and female adults (35-60 y.); *n=*1653 | FFQ to estimate average food use during the last week; Interviewer-administered | FGI: Dietary diversity. Count of nine food groups (cereals; tubers and plantains; pulses; vegetables; fruits; milk and dairy products; meats, offal and poultry; fish; and oils, fat) consumed. Range from 0 to 9. | Multi-adjusted logistic regression | Mixed association: Compared to adults with low dietary diversity, those with moderate dietary diversity were less likely to be overweight (AOR: 0.7, 95%CI: 0.49, 0.97) but not those with high dietary diversity (AOR: 0.8, 95%CI: 0.46, 1.34). |
| Truthmann et al. (38) | 2012 | Cross. | Germany | Male and female adolescents (12-17 y.); *n=*5198 | Semi-quantitative FFQ to estimate average food use during the last few weeks (45 food items); Self-administered; Five portion sizes illustrated by pictures | DGI: HFD Index. Multiplication of the Berry Index by the health value of the diet (which is the sumt of volume share of each food multiplied by a health factor based on the German Nutrition Society). Range from 0 to 1. | Multi-adjusted linear regression | Null association: No association was found between obesity and HFD Index. |
| Hadgu et al. (58) | 2013 | Cross. | Ethiopia | Female adults (≥20 y.); *n=*376 | One qualitative 24-hr recall; Interviewer-administered | FGI: DDS. Count of nine food groups consumed daily. Range from 0 to 9. | Multi-adjusted logistic regression | Favorable association: Women with low DDS were more likely to be undernourished (BMI < 18.5 kg/m²) (AOR: 1.19, 95%CI: 1.08, 1.75). |
| Jayawardena et al. (53) | 2013 | Cross. | Sri lanka | Male and female adults (≥18 y.); *n=*481 | One quantitative 24-h recall; Interviewer-administered; Food photographs and common household utensils | FII: FVS. Count of different foods consumed daily.  FGI: DDS. Count of twelve food groups (starch; vegetables; green leafy vegetables; fruits; fish; meat; legumes; milk; beverages; oils and fats; sweets and miscellaneous) consumed daily. Range from 0 to 12.  DGI: DDSP. Count of eight food groups considered as major in the Sri Lankan food pyramid consumed daily. Range from 0 to 8. Minimum reported amount for inclusion (one serving). | ANOVA | Unfavorable association: BMI and waist circumference increased with increasing FVS, DDS and DDSP. |
| Kadiyala et al. (61) | 2013 | Cross. | Uganda | Male and female adults; *n=*902 | One 24-h recall; Interviewer-administered; | FGI: IDDS. Count of twelve food groups (cereals; roots and tubers; pulse, legumes, nuts; vegetables; fruits; meat and poultry; eggs; fish and seafood; milk and milk products; oils and fats; sugar and sweets; and condiments and miscellaneous). Range from 0 to 12. | Multi-adjusted linear and logistic regression | Favorable association: IDDS independently predict BMI and MUAC. Adults with high IDDS were less likely to be undernourished (BMI < 18.5 kg/m²) (AOR: 0.563) and wasted (MUAC < 230mm for males and 220mm for females) (AOR: 0.179 but non-significant) |
| Keding et al. (55) | 2013 | Cross. | Tanzania | Female adults (15-45 y.); *n=*210 | One semi-quantitative 24-hr recall; Interviewer-administered; Three containers to estimate portion sizes | FII: FVS. Count of food items consumed daily.  FGI: DDS. Count of fourteen food groups consumed daily. Range from 0 to 14. | Univariate linear regression | Unfavorable association: Higher FVS and DDS, and especially FVS, were associated with higher BMI values (food diversity increasing by consumption of sugar, cakes and tea). |
| Vakili et al. (48) | 2013 | Cross. | Iran | Female adolescents (15-18 y.); *n=*506 | One qualitative 24-hr recall (closed-list); Self-administered | FGI: DDS. Count of fourteen food groups consumed daily. Range from 0 to 14. | Univariate logistic regression | Null association: Higher DDS was not associated with abnormal BMI, waist circumference nor waist hip ratio. |
| Ali et al. (30) | 2014 | Cross. | Pakistan | Pregnant female adults (17-40 y.); *n=*350 | One qualitative dietary recall of the foods and drinks consumed over the previous 3 days; Interviewer-administered; | FGI: Dietary diversity score. Count of ten food groups consumed daily and average over the 3 days. Range from 0 to 10. Minimum reported amount for inclusion (almost once a day). | Univariate linear regression | Mixed association: Even though weight gain during second and third trimesters had a positive relationship with dietary diversity, ≥74% of pregnant women gained less than recommended level of weight gain. |
| Tiew et al. (26) | 2014 | Cross. | Malaysia | Male and female adults (≥18 y.); *n=*113 | Quantitative FFQ to estimate average food use during the last month (28 food items); Interviewer-administered; Household measurement tools | FGI: FGS. Count of five food groups (grains; fruits; vegetables; fish, meat, eggs, and legumes; and dairy) consumed daily. Range from 0 to 5. Minimum reported amount for inclusion (half-serving).  DGI: SS. Count of five food groups (grains; fruits; vegetables; fish, meat, eggs, and legumes; and dairy) consumed daily. Achieving the minimum recommended number of servings per group provided up to 4 points (four servings of grains and two for each other groups based on the recommendations from dietary guidelines of Malaysia). Range from 0 to 20. Minimum reported amount for inclusion (half-serving). | Multi-adjusted linear regression | Mixed association: Adults of normal weight were found to have higher FGS and SS than their overweight, pre-obese or obese counterparts. On the other hand, waist-to-hip ratio was positively associated with SS (OR: 9.58, 95%CI: 1.24, 17.92) but not FVS. |
| Azadbakht et al. (17) | 2015 | Cross. | Iran | Female adolescents (11-13 y.); *n=*265 | Semi-quantitative FFQ to estimate average food use during the last year (53 food items); Self-administered | FGI: DDS. Count of food items consumed among each of the five main food groups (grain, bread; vegetables; fruits; meats; dairy) with a maximal score up to 2. Sum of the scores from the five main food groups. Range from 0 to 10. | ANCOVA | Favorable association: Higher tercile of DDS had significantly lower BMI, waist circumference and hip circumference. Prevalence of overweight and obesity and abdominal adiposity were significantly higher in the lowest tercile of DDS. |
| Benzekri et al. (65) | 2015 | Cross. | Senegal | Male and female adults (19-67 y.); *n=*109 | One 24-hr recall; Interviewer-administered | FGI: IDDS. Count of twelve food groups (cereals; roots and tubers; pulse, legumes, nuts; vegetables; fruits; meat and poultry; eggs; fish and seafood; milk and milk products; oils and fats; sugar and sweets; and condiments and miscellaneous). Range from 0 to 12. | T-test | Null association: IDDS was not associated with malnutrition (BMI<18.5 kg/m²). |
| Florêncio et al. (23) | 2015 | Long. | Brazil | Female adults (18-45 y.); *n=*85 | Three non-consecutive 24-hr recalls on random days including a weekend day; Interviewer-administered; Photographic food manual | FGI: Dietary diversity score. Count of twelve food groups (cereals; roots and tubers; pulse, legumes, nuts; vegetables; fruits; meat and poultry; eggs; fish and seafood; milk and milk products; oils and fats; sugar and sweets; and condiments and miscellaneous). Range from 0 to 12. | Multi-adjusted linear regression | Favorable association: Dietary diversity score was inversely associated with weight gain (B:-1.039, 95%CI: -2.010, -0.067). |
| Otto et al. (2) | 2015 | Long. | USA | Male and female adults (45-84 y.); *n=*5160 | Quantitative FFQ to estimate average food use during the last year (120 food items); Self-administered; Portion sizes (small, medium and large) | FII: Food count. Count of food items consumed. Range from 0 to 120. Minimum reported amount for inclusion (once a week).  OI:   - Evenness. Represents the number of different foods consumed and how evenly the amounts consumed are distributed. Range from 0 to 1. Minimum reported amount for inclusion (once a week). - Dissimilarity. Average distance for pairwise comparisons of all food items consumed by each participant using 12 different food attributes based on likely evidence for associations on cardio-metabolic health. Range from 0 to 1. Minimum reported amount for inclusion (once a week). | Multi-adjusted linear regression | Mixed association: 5-year change in waist circumference was positively associated with Dissimilarity (unfavorable association), but not Food Count or Evenness (null association). |
| Vadiveloo et al. (19) | 2015 | Cross. | USA | Male and female adults (≥20 y.); *n=*7470 | Two non-consecutive quantitative 24-hr recalls within a 2-week period (1 weekday and 1 weekend day); Interviewer-administered | DGI: US HFD index. Multiplication of the Berry Index by the health value of the diet (which is the sum of volume share of each food multiplied by a health factor based on the US Dietary Guidelines). Range from 0 to 1. | Multi-adjusted logistic regression | Favorable association: Odds of obesity, waist-to-height ratio ≥0.5, android-to-gynoid ratio >1 and high fat mass index were lower among adults in quintile 5 vs. quintile 1 of the US HFD index (about 31–55% in women and 40–48% in men). |
| Vadiveloo et al. (18) | 2015 | Cross. | USA | Male and female adults (≥20 y.); *n=*7470 | Two non-consecutive quantitative 24-hr recalls within a 2-week period; Interviewer-administered | DGI: US HFD index. Multiplication of the Berry Index by the health value of the diet (which is the sum of volume share of each food multiplied by a health factor based on the US Dietary Guidelines). Range from 0 to 1. | Multi-adjusted logistic regression | Favorable association: Elevated waist circumference were lower in higher tercile of US HFD index compared to lower tercile (OR: 0.75, 95%CI: 0.66, 0.86) |
| Abubakari et al. (31) | 2016 | Cross. | Ghana | Pregnant female adults (≥16 y.); *n=*578 | FFQ to estimate average food use since women became pregnant or were pregnant (55 food items); Interviewer-administered | FGI: WDDS. Count of nine food groups (starchy staples and white roots; vitamin A-rich fruits and vegetables; dark green leafy vegetables; other fruits and vegetables; organ meat; meat and fish; eggs; legumes, nuts and seeds; and milk and milk products) consumed daily. Range from 0 to 9. | Univariate logistic regression | Mixed association: Higher DDS were observed among mothers who were overweight and obese but lower DDS was observed in mothers who were underweight before pregnancy. Higher DDS was also observed among mothers who gained excessive weight during pregnancy. |
| Amugsi et al. (32) | 2016 | Cross. | Ghana, Namibia and Sao Tome and Principe | Female adults (15-45 y.); *n=*6372 | One 24-hr recall; Interviewer-administered | FGI: DDS. Count of nine food groups (starchy staples and white roots; vitamin A-rich fruits and vegetables; dark green leafy vegetables; other fruits and vegetables; organ meat; meat and fish; eggs; legumes, nuts and seeds; and milk and milk products) consumed daily. Range from 0 to 9. | Multi-adjusted linear regression | Mixed association: DDS was positively associated with maternal BMI in Ghana and Namibia but not in Sao Tome. |
| Tavakoli et al. (37) | 2016 | Cross. | Iran | Female adults (≥60 y.); *n=*292 | Two non-consecutive quantitative 24-hr recalls within a 1-wk period (weekdays); Interviewer-administered; Visual aids, food portion models and common household utensils | FGI: DDS. Count of food items consumed among each of the five main food groups (grain, bread; vegetables; fruits; meats; dairy) with a maximal score up to 2. Sum of the scores from the five main food groups. Range from 0 to 10. Minimum reported amount for inclusion (half-serving over two days). | Univariate linear regression | Null association: DDS was not associated with BMI and waist circumference. |
| Vadiveloo et al. (20) | 2016 | Long. | USA | Male and female adults (30-70 y.); *n=*356 | Three non-consecutive quantitative 24-hr recalls; Self-administered | DGI: US HFD index. Multiplication of the Berry Index by the health value of the diet (which is the sum of volume share of each food multiplied by a health factor based on the US Dietary Guidelines). Range from 0 to 1. | Multi-adjusted linear regression | Favorable association: Increasing US HFD Index during a 6-month energy-restricted diet was associated with greater reduction in weight, waist circumference and body fat, both in short-term (6 months) and long-term (2 years), compared to adults with stable or decreased US HFD index. |
| Benzekri et al. (66) | 2017 | Cross. | Senegal | Male and female adults (19-67 y.); *n=*95 | One 24-hr recall; Interviewer-administered | FGI: IDDS. Count of twelve food groups (cereals; roots and tubers; pulse, legumes, nuts; vegetables; fruits; meat and poultry; eggs; fish and seafood; milk and milk products; oils and fats; sugar and sweets; and condiments and miscellaneous). Range from 0 to 12. | Multi-adjusted logistic regression | Null association: IDDS was not predictive of malnutrition (BMI<18.5 kg/m²) (OR: 1.02, p= 0.91). |
| Gali et al. (49) | 2017 | Cross. | Ethiopia | Male and female adolescents; *n=*510 | Qualitative FFQ to estimate average food use during an unknown period; Interviewer-administered | FGI: DDS. Count of unknown food groups consumed. | Multi-adjusted logistic regression | Null association: DDS was not found to be associated with overweight/obesity |
| Haws et al. (33) | 2017 | Long. | USA | Female adults (21-50 y.); *n=*134 | From 2 to 12 quantitative 24-hr recall within a 16 days period (randomly selected days); Interviewer-administered; Food models, Measuring utensils and standardized food amounts booklets | FII and FGI:   - Daily overall variety. Count of individual foods, specific groups (range from 0 to 150) or broad groups (range from 0 to 15) consumed daily and average over two weeks.   Cumulative overall variety: Count of individual foods, specific groups (range from 0 to 150) or broad groups (range from 0 to 15) consumed over two weeks. | Pearson’s correlation | Mixed association: Daily overall variety was positively associated with weight loss (r=0.29 when based on individual foods, r=0.28 when specific groups and r=0.33 when broad groups) but not cumulative overall variety (r=-0.06 when based on individual foods, r=0.02 when specific groups and r=-0.004 when broad groups). |
| Ishikawa et al. (34) | 2017 | Cross. | Japan | Male and female adults (65-74 y.); *n=*307 | Two days quantitative food record; Interviewer-administered | FII: Food diversity. Count of food items consumed daily and average over two days.  FGI: Food group diversity. Count of fourteen food groups (cereals; potatoes; sugar and sweeteners; beans, nuts and seeds; green and yellow vegetables; white vegetables; fruits; fungi; algae; fish and shellfish; meat; eggs; milk and milk products; fats and oils; and confectionery) consumed daily and average over two days. Range from 0 to 15. | Multi-adjusted logistic regression | Mixed association: Low food diversity was associated with obesity (OR: 1.95, 95%CI: 1.12, 3.38, p=0.018) in women but not men. Food group diversity was not associated with obesity. |
| Nachvak et al. (25) | 2017 | Cross. | Iran | Male and female adults; *n=*190 | Semi-quantitative FFQ to estimate average food use during the last year (168 food items); Interviewer-administered; Standard serving size | FGI: DDS. Count of food items consumed among each of the five main food groups (grain, bread; vegetables; fruits; meats; dairy) with a maximal score up to 2. Sum of the scores from the five main food groups. Range from 0 to 10. Minimum reported amount for inclusion (half-serving per day). | ANOVA | Mixed association: Higher DDS was significantly associated with lower waist-to-hip ratio in men but not in women. DDS was not associated with BMI, waist circumference nor hip circumference. |
| Tian et al. (6) | 2017 | Cross. | China | Male and female adults (18-65 y.); *n=*17825 | Three consecutive quantitative 24-hr recalls; Interviewer-administered; Food models and pictures | FGI: DDS. Count of six food groups (grains; vegetables; fruits; meat, poultry and seafood; dairy; and beans, eggs and nuts) consumed daily. Range from 0 to 14. Minimum reported amount for inclusion (25 g).  OI: Entropy. Count of the shares of each food group in the total amount of food consumed multiplied by neperian logarithm of the inverse of the shares of each food group. | Multi-adjusted logistic regression | Mixed association: DDS and entropy increased the risk of overweight in men (DDS, OR: 1.09, 95%CI: 1.03, 1.17; entropy, OR: 1.60, 95%CI: 1.24, 2.07), but not in women (DDS, OR: 1.05, 95%CI: 0.99, 1.11; entropy, OR: 1.20, 95%CI: 0.94, 1.53). DDS and entropy were not associated with the risk of obesity. |
| Tsuchiya et al. (35) | 2017 | Case. | Solomon Islands | Male and female adults (≥20 y.); *n=*114 | One quantitative 24-hr recall; Interviewer-administered; Photos with three portion sizes (for IDDS calculation)  Qualitative FFQ to estimate average food use during the last 6 months (11 food items); Self-administered (for FDSK calculation) | FGI:   - IDDS. Count of fourteen food groups (cereals; vitamin A-rich vegetables; white tubers and roots; dark green leafy vegetables; other vegetables; vitamin A-rich fruits; other fruit; organ meat; flesh meats; eggs; fish; legumes, nuts and seeds; milk and milk products; oils and fats) consumed daily (based on 24-hr). Range from 0 to 14. - FDSK-11. Count of eleven food groups (grain; meat; fish and shellfish; eggs; milk; beans and bean products; potatoes; vegetables; seaweed; nuts; and fruits) consumed (based on FFQ). Range from 0 to 11. Minimum reported amount for inclusion (once a week). | Multi-adjusted logistic regression | Mixed association: Lower risk of being obese with higher IDDS (AOR: 0.71, 95%CI: 0.51, 0.99) with but not with higher FDSK-11. |
| Zhang et al. (57) | 2017 | Cross. | China | Male and female adults (≥18 y.); *n=*1105 | Three consecutive quantitative 24-hr recalls (including weekdays and one weekend day); Interviewer-administered | FGI: DDS. Count of nine food groups (cereals, tubers, and roots; vegetables; fruits; meat; beans nuts and seeds; eggs, fish and seafood; dairy; and oil) consumed over the 3 days. Range from 0 to 9. | Multi-adjusted logistic regression | Unfavorable association: Higher DDS was associated to higher risk of general obesity (OR: 1.9, 95%CI: 1.1,3.7) and of central obesity (OR: 1.9, 95%CI: 1.3, 2.8). |
| Farhangi et al. (51) | 2018 | Cross. | Iran | Male and female adults (≥20 y.); *n=*160 | Semi-quantitative FFQ to estimate average food use during the last year (147 food items); Self-administered; Standard serving size | FGI: DDS. Count of food items consumed among each of the five main food groups (grain, bread; vegetables; fruits; meats; dairy) with a maximal score up to 2. Sum of the scores from the five main food groups. Range from 0 to 10. Minimum reported amount for inclusion (half-serving per day). | ANOVA and Chi2 | Unfavorable association: High prevalence of obesity was observed in highest quartile of DDS |
| Karimbeiki et al.(52) | 2018 | Case. | Iran | Male and female adults (≥18 y.); *n=*500 | Semi-quantitative FFQ to estimate average food use during the last year (168 food items); Self-administered; Standard serving size and household measures | FGI: DDS. Count of food items consumed among each of the five main food groups (grain, bread; vegetables; fruits; meats; dairy) with a maximal score up to 2. Sum of the scores from the five main food groups. Range from 0 to 10. Minimum reported amount for inclusion (half-serving per day). | Multi-adjusted logistic regression | Unfavorable association: Each unit increase in DDS increased the probability of being obese (OR: 1.34, 95%CI: 1.07, 1.68, significant) and being overweight (OR: 1.16, 95%CI: 0.94, 1.45, non-significant). |
| Nithya et al. (63) | 2018 | Cross. | India | Male and female adolescents (≥13 y.); *n=*1645 | One quantitative 24-hr recall; Interviewer-administered | FGI: Food score. Count of thirteen food groups. Range from 0 to 13. | Multi-adjusted linear regression | Mixed association: Food score was not associated with BMI Z-scores and HAZ in adolescents while food score was positively associated with BMI in adults. |

1 Case., Case-control; Cross., Cross-sectional; Long., Longitudinal

**Supplemental Table 3.** Summary of 41 studies evaluating the association between dietary diversity indicators and non-communicable diseases and intermediate biomarkers of health in participants aged 10 years and older^1^.

| Study (ref) | Year | Design | Country | Population | Dietary assessment | Dietary diversity indicator | Statistical methods | Health Outcome | Associations |
| --- | --- | --- | --- | --- | --- | --- | --- | --- | --- |
| McCann et al. (73) | 1994 | Case. | USA | Male and female adults; *n=*856 | Quantitative FFQ to estimate average food use during the year preceding diagnosis or interview (128 food items); Self-administered; Portion size | FII: Total diet diversity. Count of foods consumed among 6 food groups (fruits; vegetables; grains; dairy; meats; nutrient nondense foods) after having standardized for the number of food in each food group (z-score). Range from 0 to 114. Minimum reported amount for inclusion (once a month). | Multi-adjusted logistic regression | Cancer | Mixed association: Colon cancer risk was positively associated with total diet diversity in men (OR: 1.99, 95%CI: 0.95, 4.15) but not in women (OR: 0.75, 95%CI: 0.37, 1.53). |
| Fernandez et al. (67) | 1996 | Case. | Italy | Male and female adults (19-74 y.); *n=*3350 | FFQ to estimate average food use during an unknown period prior to cancer diagnosis or hospital admission (29 food items); Interviewer-administered | FII: Diet diversity. Count of different food items consumed. Minimum reported amount for inclusion (once a week). | Multi-adjusted logistic regression | Cancer | Favorable association: Colorectal cancer risk was inversely associated with total diet diversity (RR: 0.7, 95%CI: 0.6, 0.9). |
| La Vecchia et al. (68) | 1997 | Case. | Italy | Male and female adults (19-74 y.); *n=*2799 | FFQ to estimate average food use during the last year prior to cancer diagnosis or hospital admission (29 food items); Interviewer-administered | FII: Diet diversity. Count of different food items consumed. Minimum reported amount for inclusion (once a week). | Multi-adjusted logistic regression | Cancer | Favorable association: Gastric cancer risk was inversely associated with total diet diversity (OR: 0.7, 95%CI: 0.5, 0.9). |
| Slattery et al. (41) | 1997 | Case. | USA | Male and female adults (30-79 y.); *n=*4403 | Diet history questionnaire with a referent period of a year (800 food items, 69 food groups); Interviewer-administered; Food models and plastic cups and spoons | FII: Total diet diversity. Count of different food items reported, excluding non-nutrient contributing food items such as coffee, tea, and water. | Multi-adjusted logistic regression | Cancer | Null association: Colon cancer risk was not associated with total diet diversity, either in men (OR: 1.2, 95%CI: 0.9, 1.6) or women (OR: 1.1, 95%CI: 0.8, 1.6). |
| Levi et al. (69) | 1998 | Case. | Switzerland | Male and female adults (23-75 y.); *n=*440 | FFQ to estimate average food use during the last 2 years prior to cancer diagnosis or hospital admission (79 food items); Interviewer-administered | FII: Diet diversity. Count of different food items consumed. Minimum reported amount for inclusion (once a week). | Multi-adjusted logistic regression | Cancer | Favorable association: Oral and pharyngeal cancer risk was inversely associated with highest tercile of total diversity (OR: 0.35, 95%CI: 0.17, 0.70). |
| Fernandez et al. (74) | 2000 | Case. | Italy | Old male and female adults; *n=*6107 | FFQ to estimate average food use during the last 2 years prior to cancer diagnosis or hospital admission (79 food items); Interviewer-administered | FII: Diet diversity. Count of different food items consumed. Minimum reported amount for inclusion (once a week). | Multi-adjusted logistic regression | Cancer | Mixed association: Colon cancer risk was inversely associated with diet diversity (OR: 0.76, 95%CI: 0.59, 0.99) while rectal cancer was not associated with diet diversity. |
| Garavello et al. (70) | 2008 | Case. | Italy | Male and female adults (19-79 y.); *n=*2886 | FFQ to estimate average food use during the last 2 years prior to cancer diagnosis or hospital admission (78 food items); Interviewer-administered | FII: Diet diversity. Count of different food items consumed. Minimum reported amount for inclusion (once a week). | Multi-adjusted logistic regression | Cancer | Favorable association: Oral and pharyngeal cancer risk was inversely associated with highest tercile of total diversity (OR: 0.78, 95%CI: 0.61, 0.98). |
| Lucenteforte et al. (71) | 2008 | Case. | Italy | Male and female adults (36-77 y.); *n=*1047 | FFQ to estimate average food use during the last 2 years prior to cancer diagnosis or hospital admission (78 food items); Interviewer-administered | FII: Diet diversity. Count of different food items consumed. Minimum reported amount for inclusion (once a week). | Multi-adjusted logistic regression | Cancer | Favorable association: Esophageal cancer risk was inversely associated with highest quartile of total diversity (OR: 0.42, 95%CI: 0.25, 0.71). |
| Garavello et al. (75) | 2009 | Case. | Italy and Switzerland | Male and female adults (30-79 y.); *n=*1824 | FFQ to estimate average food use during the last 2 years prior to cancer diagnosis or hospital admission (78 food items); Interviewer-administered | FII: Diet diversity. Count of different food items consumed. Minimum reported amount for inclusion (once a week). | Multi-adjusted logistic regression | Cancer | Null association: Laryngeal cancer risk was not associated with total diet diversity (OR: 0.76, 95%CI: 0.50, 1.15). |
| Isa et al. (72) | 2013 | Case. | China | Old male and female adults; *n=*956 | Qualitative FFQ to estimate average food use during the last year (35 food items); Interviewer-administered | FII: Total diet diversity score. Count of different food items consumed. Range from 0 to 35. Minimum reported amount for inclusion (once a week). | Multi-adjusted logistic regression | Cancer | Favorable association: Bladder cancer risk was inversely associated with higher diet diversity (>20 different foods a week, OR: 0.4, 95%CI: 0.2, 1.1). |
| Azadbakht et al. (24) | 2005 | Cross. | Iran | Male and female adults (18-74 y.); *n=*581 | Semi-quantitative FFQ to estimate average food use during the last year (168 food items); Interviewer-administered; Standard serving size | FGI: DDS. Count of food items consumed among each of the five main food groups (grain, bread; vegetables; fruits; meats; dairy) with a maximal score up to 2. Sum of the scores from the five main food groups. Range from 0 to 10. Minimum reported amount for inclusion (half-serving per day). | Multi-adjusted logistic regression | Metabolic syndrome | Favorable association: Having metabolic syndrome odds were lower in higher quartile of DDS compared to lower quartile (OR: 0.77, 95%CI: 0.59, 0.93). These lower odds were mostly related to the fact that having diabetes (OR: 0.78, 95%CI: 0.59, 0.98), high blood pressure (OR: 0.85, 95%CI: 0.58, 1.13) and high triglyceride level (OR: 0.84, 95%CI: 0.69, 0.99) were lower in higher quartile of DDS compared to lower quartile. |
| Azadbakht et al. (50) | 2006 | Cross. | Iran | Male and female adults (≥18 y.); *n=*581 | Semi-quantitative FFQ to estimate average food use during the last year (168 food items); Interviewer-administered; Standard serving size | FGI: DDS. Count of food items consumed among each of the five main food groups (grain, bread; vegetables; fruits; meats; dairy) with a maximal score up to 2. Sum of the scores from the five main food groups. Range from 0 to 10. Minimum reported amount for inclusion (half-serving per day). | Multi-adjusted logistic regression | Metabolic syndrome | Favorable association: Probabilities of having hypercholesterolemia, high LDL cholesterol, hypertension and diabetes decreased with increasing quartile of DDS. |
| Gregory et al. (14) | 2009 | Cross. | Guatemala | Male and female adults; *n=*1220 | FFQ to estimate average food use during the last 3 months (52 food items); Interviewer-administered | FII:   - RFS. Count of recommended food items consumed. Range from 0 to 16. Minimum reported amount for inclusion (once a week). - NRFS. Count of not recommended food items consumed. Range from 0 to 15. Minimum reported amount for inclusion (once a week). - FVS. Count of any unique food consumed (excluding alcoholic or sweetened beverages or condiments such as jam, honey, mayonnaise or butter). Range from 0 to 39. Minimum reported amount for inclusion (once a week). | Multi-adjusted linear regression | Metabolic syndrome | Mixed association: Globally, RFS, NRFS and FVS were not associated with the metabolic syndrome or its components, except for the RFS which was positively associated with triglycerides (B: 0.08; 95%CI: 0.01, 0.17) and glucose (B: 0.12; 95%CI: 0.02, 0.21) among men. |
| Kimura et al. (43) | 2009 | Cross. | China | Male and female adults (≥60 y.); *n=*240 | FFQ to estimate average food use during the last week (11 food items); Interviewer-administered | FGI: FDSK-11. Count of eleven food groups (grain; meat; fish and shellfish; eggs; milk; beans and bean products; potatoes; vegetables; seaweed; nuts; and fruits) consumed. Range from 0 to 11. | T-test | Metabolic syndrome | Mixed association: Higher FDSK-11 was associated with lower blood glucose in Han elderly subjects, but also with higher blood glucose in Tibetan elderly subjects. No association was found between FDSK-11 and triglyceride, HDL and LDL cholesterol. |
| Mohamadpour et al. (76) | 2012 | Cross. | Malaysia | Female adults (19-49 y.); *n=*169 | Quantitative FFQ to estimate average food use during an unknown period (29 food items); Interviewer-administered | FGI: DDS. Count of twenty-nine food groups consumed. Range from 0 to 29. Minimum reported amount for inclusion (twice a week). | Multi-adjusted logistic regression | Metabolic syndrome | Favorable association: Women with higher DDS were more likely to have less 3 health risks relating to metabolic syndrome (OR: 0.87, 95%CI: 0.76, 0.99). |
| Farhangi et al. (51) | 2018 | Cross. | Iran | Male and female adults (≥20 y.); *n=*160 | Semi-quantitative FFQ to estimate average food use during the last year (147 food items); Self-administered; Standard serving size | FGI: DDS. Count of food items consumed among each of the five main food groups (grain, bread; vegetables; fruits; meats; dairy) with a maximal score up to 2. Sum of the scores from the five main food groups. Range from 0 to 10. Minimum reported amount for inclusion (half-serving per day). | ANOVA and Chi2 | Metabolic syndrome | Mixed association: Higher serum triglyceride concentrations and systolic blood pressure values and lower serum adiponectin concentrations were observed lowest quartile of DDS. Fasting serum glucose, triglyceride, LDL and HDL cholesterol were not associated with DDS. |
| de Oliveira et al. (77) | 2012 | Cross. | Brazil | Male and female adults (≥35 y.); *n=*305 | One quantitative 24-hr recall; Interviewer-administered; Homemade measurements | FII: Diet variety. Count of different foods consumed. | Multi-adjusted logistic regression | Metabolic syndrome | Favorable association: Higher diet variety (≥ 8 foods) was associated to a lower metabolic syndrome risk (OR: 0.31, 95%CI: 0.12, 0.79). |
| Vadiveloo et al. (18) | 2015 | Cross. | USA | Male and female adults (≥20 y.); *n=*7470 | Two non-consecutive quantitative 24-hr recalls within a 2-week period; Interviewer-administered | DGI: US HFD index. Multiplication of the Berry Index by the health value of the diet (which is the sum of volume share of each food multiplied by a health factor based on the US Dietary Guidelines). Range from 0 to 1. | Multi-adjusted logistic regression | Metabolic syndrome | Favorable association: Having metabolic syndrome odds were lower in higher tercile of US HFD index compared to lower tercile (OR: 0.79, 95%CI: 0.64, 0.98). These lower odds were mostly related to the fact that low serum HDL cholesterol (OR: 0.83, 95%CI: 0.68, 1.01, p=0.06) and hypertension level (OR: 0.83, 95%CI: 0.70, 0.995, p<0.05) were lower in higher tercile of US HFD index compared to lower tercile. |
| Tian et al. (79) | 2017 | Cross. | China | Male and female adults (≥18 y.); *n=*4308 | Three consecutive quantitative 24-hr recalls; Interviewer-administered; Food models and pictures | FGI: DDS. Count of six food groups (grains; vegetables; fruits; meat, poultry and seafood; dairy; and beans, eggs and nuts) consumed daily. Range from 0 to 6. Minimum reported amount for inclusion (10 g for dairy and 25 g when other groups). | Multi-adjusted logistic regression | Metabolic syndrome | Mixed association: Higher DDS was associated with lower risk of having metabolic syndrome in young women (≤45) (OR: 0.37, 95%CI: 0.22-0.64) but higher risk in old women (>60) (OR: 1.69, 95%CI: 1.05, 2.72) and men (OR: 2.18, 95%CI: 1.30, 3.65). |
| Gholizadeh et al. (78) | 2018 | Cross. | Iran | Male and female adults (20-60 y.); *n=*300 | Semi-quantitative FFQ to estimate average food use during the last year (168 food items); Interviewer-administered; Standard serving size | FGI: DDS. Count of nine food groups (starch; milk; green leafy vegetables; vegetables and fruits rich in vitamin A; other fruits; meat; fish; legumes; oils and fats) consumed over the year. Range from 0 to 9. | Multi-adjusted linear and logistic regression | Metabolic syndrome | Favorable association: Having metabolic syndrome odds were lower by 80% in the fourth quartile of DDS quartile compared to first quartile. These lower odds were mostly related to the fact that higher DDS was associated with low levels of fasting blood glucose, triglyceride and HDL-cholesterol. |
| Wahlqvist et al. (81) | 1989 | Case. | Australia | Old male and female adults; *n=*53 | Seven days quantitative food record; Self-administered (followed by an interview with a nutritionist for review) ; Food models | FII: Food variety. Count of unique foods consumed. Range from 0 to 53. Minimum reported amount for inclusion (one serving over the week). | T-test and correlation analyses | Diabetes | Favorable association: Food variety was lower in adults with diabetes than healthy adults. Food variety was positively associated with arterial compliance (r=0.36, p<0.01) and negatively associated with proximal resistance in common femoral artery (r= -0.36, p<0.01) and in posterior tibial artery (r= -0.43, p<0.001). |
| Woo et al. (9) | 2003 | Cross. | Hong Kong SAR, China | Male and female adults (24-74 y.); *n=*988 | Quantitative FFQ to estimate average food use during the last week (266 food items); Pictures of food portions | OI: Variety ratio. Percentage of different food items consumed within snacks over the week divided by the sum of percentages of different food items consumed within grains, meats, fruit and vegetables over the week. | ANCOVA | Diabetes | Null association: No significant difference in variety ratio across glucose tolerance groups. |
| Mayega et al. (82) | 2013 | Cross. | Uganda | Male and female adults (35-60 y.); *n=*1497 | FFQ to estimate average food use during the last week; Interviewer-administered | FGI: Dietary diversity. Count of nine food groups (cereals; tubers and plantains; pulses; vegetables; fruits; milk and dairy products; meats, offal and poultry; fish; and oils, fat) consumed. Range from 0 to 9. | Multi-adjusted logistic regression | Diabetes | Favorable association: Adults with higher dietary diversity had a lower risk of abnormal glucose regulation (RR: 0.5, 95%CI: 0.3, 0.9). |
| Masset et al. (85) | 2015 | Long. | UK | Male and female adults (35-55 y.); *n=*7251 | Quantitative FFQ to estimate average food use during the last year (127 food items); Self-administered; Common unit or portion size | FII:   - FVS. Count of food items reported to be consumed. Minimum reported amount for inclusion (once a week). - RFVOfcom and RFV(SAIN,LIM). Count of foods identified as ‘healthy’ by the Ofcom and SAIN,LIM models. Minimum reported amount for inclusion (once a week). - NRFVOfcom and NRFV(SAIN,LIM). Count of foods identified as ‘less healthy’ aby the Ofcom and SAIN,LIM models (the higher, the worst). Minimum reported amount for inclusion (once a week). | Multi-adjusted Cox's proportional-hazards regression | Diabetes | Null association: No robust association were found between FVS, RFV(Ofcom), RFV(SAIN,LIM), NRFV(Ofcom) and NRFV(SAIN,LIM) and diabetes risk. |
| Otto et al. (2) | 2015 | Long. | USA | Male and female adults (45-84 y.); *n=*5160 | Quantitative FFQ to estimate average food use during the last year (120 food items); Self-administered; Portion sizes (small, medium and large) | FII: Food count. Count of food items consumed. Range from 0 to 120. Minimum reported amount for inclusion (once a week). OI:   - Evenness. Represents the number of different foods consumed and how evenly the amounts consumed are distributed. Range from 0 to 1. Minimum reported amount for inclusion (once a week). - Dissimilarity. Average distance for pairwise comparisons of all food items consumed by each participant using 12 different food attributes based on likely evidence for associations on cardio-metabolic health. Range from 0 to 1. Minimum reported amount for inclusion (once a week). | Multi-adjusted linear regression | Diabetes | Null association: Incident type-2 diabetes was not associated with Food Count, Evenness or Dissimilarity. |
| Azadbakht et al. (84) | 2016 | Cross. | Iran | Male and female adults (≥19 y.); *n=*1571 | Semi-quantitative FFQ to estimate average food use during the last year (48 food items); Interviewer-administered | FGI: DDS. Count of food items consumed among each of the five main food groups (grain, bread; vegetables; fruits; meats; dairy) with a maximal score up to 2. Sum of the scores from the five main food groups. Range from 0 to 10. Minimum reported amount for inclusion (once a day). | ANOVA | Diabetes | Mixed association: DDS was inversely related to dietary glycemic index but was not related to glycemic load. |
| Conklin et al. (80) | 2016 | Long. | United Kingdom | Male and female adults (40-79 y.); *n=*23238 | Semi-quantitative FFQ to estimate average food use during the last year (130 food items); Self-administered; Standard serving size | FGI: Total diet diversity score. Count of five food groups (dairy products; fruits; vegetables; grain/cereal products; and meat and alternatives) consumed. Range from 0 to 5. Minimum reported amount for inclusion (twice per week). | Multi-adjusted Cox's proportional-hazards regression | Diabetes | Favorable association: Highest total diet diversity score (=5) was associated lower incidence of type-2 diabetes (HR: 0.70, 95%CI: 0.51, 0.95) compared to lower scores (≤3). |
| Danquah et al. (83) | 2017 | Cross. | Ghana and Europe (Netherlands, England, Germany) | Male and female adults (25-70 y.); *n=*3810 | Semi-quantitative FPQ to estimate average food use during the last year (134 food items); Interviewer-administered | FGI:   - FVS. Count of twenty food groups consumed. Range from 0 to 20. Minimum reported amount for inclusion (once a week). - DDS. Count of seven food groups consumed. Range from 0 to 7. Minimum reported amount for inclusion (once a day). | Multi-adjusted logistic regression | Diabetes | Mixed association: FVS was inversely associated with type-2 diabetes risk (OR: 0.81, 95%CI: 0.71, 0.93) while DDS was not associated. |
| Gicevic et al. (86) | 2018 | Long. | USA | Female adults (24-44 y.); *n=*unknown | Semi-quantitative FFQ every 4 years to estimate average food use during the last year (131 food items); Self-administered; Portion sizes | FGI:   - FGI. Count of eight food groups (fruits and vegetables; legumes and nuts; animal flesh; eggs; dairy; grains and roots; vegetables with >130 RE/100 g; added fats and oils) consumed. Range from 0 to 8. Minimum reported amount for inclusion (10 g except for fats and oils where it was 1 g). - MDDW. Count of ten food groups (grains and starchy vegetables; pulses; nuts and seeds; dairy; animal flesh; eggs; dark green leafy vegetables; vitamin A–rich vegetables and fruits; other vegetables; other fruits) consumed. Range from 0 to 10. Minimum reported amount for inclusion (once a day). | Multi-adjusted logistic regression | Diabetes / Hypertension | Null association: FGI and MDD-W were not associated with risk of gestational diabetes mellitus and hypertensive disorders of pregnancy. |
| Miller et al. (87) | 1992 | Cross. | Saba Island (Netherlands) | Male and female adults (≥21 y.); *n=*82 | Semi-quantitative FFQ to estimate average food use during the last year (481food items); Interviewer-administered; Household dinnerware | FGI: Diet diversity. Count of five food groups (grains and tubers; vegetables; fruits; legumes; and animal products) consumed. Range from 0 to 5. Minimum reported amount for inclusion (five servings a week). | Multi-adjusted logistic regression | Hypertension | Favorable association: Lower diet diversity was associated with hypertension risk (OR: 4.25, 95%CI: 1.47, 12.30). |
| Mayega et al. (29) | 2012 | Cross. | Uganda | Male and female adults (35-60 y.); *n=*1653 | FFQ to estimate average food use during the last week; Interviewer-administered | FGI: Dietary diversity. Count of nine food groups (cereals; tubers and plantains; pulses; vegetables; fruits; milk and dairy products; meats, offal and poultry; fish; and oils, fat) consumed. Range from 0 to 9. | Multi-adjusted logistic regression | Hypertension | Null association: Dietary diversity was not associated with being hypertensive. |
| Oliveira et al. (88) | 2012 | Cross. | Brazil | Male and female adults (44-65 y.); *n=*335 | One quantitative 24-hr recall; Interviewer-administered; Household measures | FII: Dietary variety. Count of different food items consumed. | Multi-adjusted logistic regression | Hypertension | Mixed association: Higher dietary variety (≥ 8 foods) was associated with a favorable association for alterations in systolic blood pressure (OR: 0.36, 95%CI: 0.15, 0.88) but was not associated with hypertension and diastolic blood pressure. |
| Mwanri et al. (89) | 2015 | Cross. | Tanzania | Pregnant female adults (≥20 y.); *n=*902 | One 24-hr recall; Interviewer-administered | FGI: DDS. Count of sixteen food groups (cereals; white roots and tubers; vitamin A-rich vegetables and tubers; dark green leafy vegetables; other vegetables; vitamin A-rich fruits; other fruits; organ meat; flesh meat; eggs; legume, nuts and seeds; fish and sea foods; milk and milk products; oil and fats; sweets; spices, condiments and beverages). Range from 0 to 16. | Multi-adjusted logistic regression | Hypertension | Mixed association: Higher DDS was associated with high blood pressure in urban women (OR: 1.31, 95%CI: 1.20, 1.61) but not in rural women. |
| Hsu-Hage et al. (39) | 1996 | Cross. | Australia | Male and female adults (≥25 y.); *n=*545 | Assumption of a FFQ to estimate average food use during the last year | FII: Food variety. Count of unique foods consumed over the year. | Pearson's correlation adjusted for age | Biomarkers of metabolic risk | Null association: Food variety was barely correlated with SBP (r= -0.09, p=0.0326) and not correlated with fasting glucose, triglycerides, HDL and LDL cholesterol. |
| Bernstein et al. (27) | 2002 | Cross. | USA | Male and female adults (72-98 y.); *n=*98 | Three consecutive days food records; Interviewer-administered; Weighted | FII: Dietary variety score. Count of different foods consumed during 3 days. | Multi-adjusted linear regression | Biomarkers of metabolic risk | Mixed association: Dietary variety score was associated with HDL cholesterol (B: 1.02), VLDL cholesterol (B: -3.58) and triglycerides (B: -3.51) in men but not in women. |
| Kant et al. (15) | 2005 | Cross. | USA | Male and female adults (≥20 y.); *n=*8719 | One quantitative 24-hr recall; Interviewer-administered; Abstract food models, special charts, measuring cups, and rulers | FII: FRS. Count of foods considered to be recommended consumed (low fat dairy; lean meats, poultry, fish, and alternatives; low fat whole grains such as whole wheat breads and cereals; all fruits and juices; all vegetables that were not fried, pickled, or creamed). Minimum reported amount for inclusion (15 g for non-beverages and 30 g for beverages). FGI: DDS-R. Count of five food groups (dairy; meat; grain; fruit; and vegetable) consumed daily. Range from 0 to 5. Minimum reported amount for inclusion (15 g for non-beverages and 30 g for beverages). | Multi-adjusted linear regression | Biomarkers of metabolic risk | Favorable association: RFS and DDS-R were independent negative predictors of serum homocysteine, serum C-reactive protein, plasma glucose, hemoglobin A1C, systolic and diastolic blood pressure, total serum cholesterol. |
| Drescher et al. (1) | 2007 | Cross. | Germany | Male and female adults; *n=*4030 | Validated computerized dietary history method about the diet of the preceding month; Interviewer-administered | FII: Count Index. Count of different foods consumed.  DGI: Berry Index. Represents the number of different species consumed and how evenly the amounts consumed are distributed. Range from 0 to 1. OI: HFD Index. Multiplication of the Berry Index by the health value of the diet (which is the sum of volume share of each food multiplied by a health factor based on the German Nutrition Society). Range from 0 to 1. | Pearson’s correlation | Biomarkers of metabolic risk | Favorable association: HFD Index was positively associated with serum HDL cholesterol concentration (r=0.169) and negatively associated with serum triacylglycerol, uric acid, and homocysteine (r from -0.098 to -0.065). Berry Index and Count Index showed similar correlations but with lower magnitude. |
| Truthmann et al. (38) | 2012 | Cross. | Germany | Male and female adolescents (12-17 y.); *n=*5198 | Semi-quantitative FFQ to estimate average food use during the last few weeks (45 food items); Self-administered; Five portion sizes illustrated by pictures | DGI: HFD Index. Multiplication of the Berry Index by the health value of the diet (which is the sum of volume share of each food multiplied by a health factor based on the German Nutrition Society). Range from 0 to 1. | Multi-adjusted linear regression | Biomarkers of metabolic risk | Mixed association: While a negative association was found between homocysteine and HFD Index in boys but not in girls. Uric acid, CRP, total cholesterol, LDL and HDL cholesterol, systolic and diastolic blood pressure were not associated with HFD Index. |
| Dzien et al. (90) | 2011 | Cross. | - | Male and female adolescents and adults (11-94 y.); *n=*2548 | Semi-quantitative FFQ to estimate average food use during the last week (51 food items); Self-administered | FII: Food consumption variety. Count of unique foods consumed over the week. Range from 0 to 51. | Multi-adjusted logistic regression | Cardiovascular disease | Favorable association: Consumption of one additional food item reduced the probability of a cardiovascular disease by about 0.5% (AME: -0.478). |
| Fung et al. (91) | 2018 | Long. | USA | Male and female adults (25-75 y.); *n=*212142 | Semi-quantitative FFQ to estimate average food use during the last year (135 food items); Self-administered; Standard serving sizes | FGI:   - FGI. Count of eight food groups (fruits and vegetables; legumes and nuts; animal flesh; eggs; dairy; grains and roots; vegetables with >130 RE/100 g; added fats and oils) consumed. Range from 0 to 8. Minimum reported amount for inclusion (once a week). - MDDW. Count of ten food groups (grains and starchy vegetables; pulses; nuts and seeds; dairy; animal flesh; eggs; dark green leafy vegetables; vitamin A–rich vegetables and fruits; other vegetables; other fruits) consumed. Range from 0 to 10. Minimum reported amount for inclusion (once a day). | Multi-adjusted Cox's proportional-hazards regression | Cardiovascular disease | Mixed association: Higher MDDW was associated with lower risk of ischemic heart disease pooled RR of 0.93 for MDDW (RR: 0.93, 95%CI: 0.90, 0.96) while higher FGI was not associated (RR: 0.98, 95%CI: 0.95, 1.01). |
| Hashemi Kani et al. (92) | 2013 | Case. | Iran | Male and female adults; *n=*200 | Three consecutive days quantitative food record (2 weekdays and 1 weekend day); Interviewer-administered; Household measures | FGI: DDS. Count of food items consumed among each of the five main food groups (grain, bread; vegetables; fruits; meats; dairy) with a maximal score up to 2. Sum of the scores from the five main food groups. Range from 0 to 10. Minimum reported amount for inclusion (half-serving). | Multi-adjusted logistic regression | Non-alcoholic fatty liver diseases | Null association: DDS was not associated with non-alcoholic fatty liver diseases (OR: 1.30, 95%CI: 0.56, 2.14). |

1 Case., Case-control; Cross., Cross-sectional; Long., Longitudinal

**Supplemental Table 4.** Summary of 19 studies evaluating the association between dietary diversity indicators and biomarkers of nutritional status in participants aged 10 years and older^1^.

| Study (ref) | Year | Design | Country | Population | Dietary assessment | Dietary diversity indicator | Statistical methods | Health Outcome | Associations |
| --- | --- | --- | --- | --- | --- | --- | --- | --- | --- |
| Clausen et al. (42) | 2005 | Cross. | Botswana | Male and female adults (≥60 y.); *n=*372 | Qualitative FFQ to estimate average food use during an unknown period (21 food items); Interviewer-administered | FII: FVS. Count of the frequency scores (0 to 7) of 16 selected food consumed. Range from 0 to 112. Minimum reported amount for inclusion (once a week). | T-test | Anemia | Null association: FVS was not different across old adults with or without anemia. |
| Gebremedhin et al. (97) | 2011 | Cross. | Ethiopia | Female adults (15-49 y.); *n=*5963 | One 24-hr recall; Interviewer-administered | FGI: DDS. Count of twelve food groups (cereals; roots and tubers; pulse, legumes, nuts; vegetables; fruits; meat and poultry; eggs; fish and seafood; milk and milk products; oils and fats; sugar and sweets; and condiments and miscellaneous). Range from 0 to 12. | Univariate logistic regression | Anemia | Favorable association: Women with low DDS had higher odds of having anemia (OR: 1.33, 95%CI: 1.11, 1.58) compared with those with medium and high DDS. |
| Rawat et al. (102) | 2013 | Long. | Uganda | Male and female adults; *n=*876 | One 24-hr recall; Interviewer-administered | FGI: IDDS. Count of twelve food groups (cereals; roots and tubers; pulse, legumes, nuts; vegetables; fruits; meat and poultry; eggs; fish and seafood; milk and milk products; oils and fats; sugar and sweets; and condiments and miscellaneous). Range from 0 to 12. | Multi-adjusted linear and logistic regression | Anemia | Null association: IDDS was not associated with CD4 count (B: 2.153, SE: 1.403) nor moderate anemia (OR: 1.07, 95%CI: 0.83, 1.38). |
| Abriha et al. (95) | 2014 | Cross. | Ethiopia | Pregnant female adults (16-40 y.); *n=*619 | One 24-hr recall; Interviewer-administered | FGI: DDS. Count of unknown food groups consumed. | Multi-adjusted logistic regression | Anemia | Favorable association: Pregnant women with low DDS had higher odds of having anemia (OR: 12.82, 95%CI: 6.42, 25.62) compared with those with high DDS. |
| Ali et al. (30) | 2014 | Cross. | Pakistan | Pregnant female adults (17-40 y.); *n=*350 | One qualitative dietary recall of the foods and drinks consumed over the previous 3 days; Interviewer-administered; | FGI: Dietary diversity score. Count of ten food groups consumed daily and average over the 3 days. Range from 0 to 10. Minimum reported amount for inclusion (almost once a day). | Univariate linear regression | Anemia | Null association: Dietary diversity was not associated with hemoglobin status of pregnant women. |
| Roba et al. (99) | 2015 | Long. | Ethiopia | Lactating female adults (15-49 y.); *n=*216 | One qualitative 24-hr recall; Interviewer-administered | FGI: WDDS. Count of nine food groups consumed daily. Range from 0 to 9. | Multi-adjusted linear regression | Anemia | Mixed association: WDDS was positively associated with hemoglobin level (B: 0.29, SE: 0.11) during postharvest season but was not associated (B: 0.02, SE: 0.13) during preharvest season. |
| Saaka et al. (94) | 2015 | Cross. | Ghana | Pregnant female adults (18-38 y.); *n=*307 | Quantitative FFQ to estimate average food use during the past week (11 food items); Interviewer-administered | FGI: IDDS. Count of eleven food groups (flesh meats; fish; eggs; milk and milk products; organ meat; legumes; cereals; roots and tubers; dark green leafy vegetables; vitamin A-rich fruits; and fats and oils) consumed. Each food group was assigned a score of 0 if not consumed during the previous week, 1 if consumed on 1–3 days, and 2 if consumed for at least 4 days. Range from 0 to 22. | Multi-adjusted linear regression | Anemia | Favorable association: High IDDS (>7) was positively associated with hemoglobin level in the third trimester of pregnancy (B: 0.24, 95%CI: 0.39, 0.95). |
| Abubakari et al. (31) | 2016 | Cross. | Ghana | Pregnant female adults (≥16 y.); *n=*578 | FFQ to estimate average food use since women became pregnant or were pregnant (55 food items); Interviewer-administered | FGI: WDDS. Count of nine food groups (starchy staples and white roots; vitamin A-rich fruits and vegetables; dark green leafy vegetables; other fruits and vegetables; organ meat; meat and fish; eggs; legumes, nuts and seeds; and milk and milk products) consumed daily. Range from 0 to 9. | Univariate logistic regression | Anemia | Favorable association: higher DDS were observed among mothers who had normal hemoglobin level (≥10.5 g/dL). |
| Zerfu et al. (93) | 2016 | Long. | Ethiopia | Pregnant female adults; *n=*374 | Four non-consecutive 24-hr recalls collected each month from enrollment to delivery; Interviewer-administered | FGI: WDDS. Count of nine food groups (cereals, roots and tubers; vitamin A–rich fruit and vegetables; other fruit; other vegetables; legumes and nuts; meat, poultry, and fish; fats and oils; dairy; and eggs) consumed over the 4 days. Range from 0 to 9. | Multi-adjusted logistic regression | Anemia | Favorable association: Women in the inadequate WDDS group had a higher risk of being anemic (RR: 2.29, 95%CI: 1.62, 3.24). |
| Abay et al. (96) | 2017 | Cross. | Ethiopia | Pregnant female adults; *n=*761 | One 24-hr recall; Interviewer-administered | FGI: DDS. Count of nine food groups consumed daily. Range from 0 to 9. | Multi-adjusted logistic regression | Anemia | Favorable association: Women with higher DDS (≥4) had lower odds of having anemia (OR: 0.58, 95%CI: 0.38-0.93) compared with those with lower DDS. |
| Lebso et al. (98) | 2017 | Cross. | Ethiopia | Pregnant female adults (15-49 y.); *n=*504 | One 24-hr recall; Interviewer-administered | FGI: WDDS. Count of nine food groups (cereals, starchy staples; oils, fat; dark green leafy vegetables and vitamin A rich fruits and vegetables; legumes, nuts and seeds; other fruits and vegetables; meat and fish; organ meat; milk and products; and egg) consumed daily. Range from 0 to 9. | Multi-adjusted logistic regression | Anemia | Favorable association: Women with low WDDS had higher odds of having anemia (OR: 3.18, 95%CI: 1.37, 7.37) compared with those with high DDS. |
| Saaka et al. (103) | 2017 | Cross. | Ghana | Pregnant female adults (15-49 y.); *n=*400 | One qualitative 24-hr recall (closed-list); Interviewer-administered | FGI: MDDW. Count of ten food groups (starchy staple foods; beans; peas; nuts; seeds; dairy; flesh foods; eggs; vitamin A-rich dark green leafy vegetables; other vitamin A-rich vegetables and fruits; other vegetables; and other fruits) consumed daily. Range from 0 to 10. Dichotomy indicator based on 5 food groups. | Multi-adjusted linear regression | Anemia | Null association: MDDW was not associated with hemoglobin level. |
| Alaofè et al. (100) | 2017 | Cross. | Benin | Female adults (15-49 y.); *n=*765 | One qualitative 24-hr recall; Interviewer-administered | FGI: DDS. Count of nine food groups (starchy staples and white roots; vitamin A-rich fruits and vegetables; dark green leafy vegetables; other fruits and vegetables; organ meat; meat and fish; eggs; legumes, nuts and seeds; and milk and milk products) consumed daily. Range from 0 to 9. | Multi-adjusted logistic regression | Anemia and micronutrient status | Mixed association: Women with low DDS had higher odds of iron deficiency risk (OR: 2.13, 95%CI=1.68, 6·61) compared with those with high DDS. DDS was not associated with anemia and vitamin A deficiency risks. |
| Korkalo et al. (101) | 2017 | Cross. | Mozambique | Female adolescents (14-19 y.); *n=*450 | One 24-hr recall; Interviewer-administered;  Qualitative FFQ to estimate average food use during the last week (37 food items); Interviewer-administered; | FGI:   - WDDS. Count of nine food groups (cereals, starchy staples; oils, fat; dark green leafy vegetables and vitamin A rich fruits and vegetables; legumes, nuts and seeds; other fruits and vegetables; meat and fish; organ meat; milk and products; and egg) consumed daily (based on 24-hr recall). Range from 0 to 9. - WDDS15g. Count of the same nine food groups consumed daily (based on 24-hr recall). Range from 0 to 9. Minimum reported amount for inclusion (15 g). - 7dWDDS. Count of the same nine food groups consumed over 7 days (based on FFQ). Range from 0 to 9. | Multi-adjusted logistic regression | Anemia and micronutrient status | Mixed association: Women with low WDDS, WDDS15g and 7dWDDS had higher odds of having low serum zinc (OR: 3.85, 95%CI: 1.57, 9.46; OR: 2.32, 95%CI: 1.04, 5.18; OR: 3.47, 95%CI: 1.25, 9.60; respectively). WDDS, WDDS15g and 7dWDDS were not associated with hemoglobin, serum ferritin, folate and plasma retinol levels. |
| Gebremedhin et al. (105) | 2011 | Cross. | Ethiopia | Pregnant female adults (15-49 y.); *n=*700 | One 24-hr recall; Interviewer-administered | FGI: DDS. Count of twelve food groups (cereals; roots and tubers; pulse, legumes, nuts; vegetables; fruits; meat and poultry; eggs; fish and seafood; milk and milk products; oils and fats; sugar and sweets; and condiments and miscellaneous). Range from 0 to 12. | Multi-adjusted logistic regression | Micronutrient status | Favorable association: Women with low DDS had higher odds of zinc deficiency risk (OR: 1.87, 95%CI: 1.02, 2.91 when DDS=[4-5]; OR: 2.57, 95%CI: 1.57, -4.18 when DDS ≤ 3) compared with those with high DDS (≥6). |
| Fujita et al. (106) | 2012 | Cross. | Kenya | Lactating female adults (18-46 y.); *n=*214 | One quantitative 24-hr recall; Interviewer-administered; Common household utensils, food models and filling utensils | FGI: DDS. Count of ten food groups (cereals, tubers; meat, poultry, fish; vitamin A-rich fruits and vegetables; other vegetables; other fruit; oils and fats; dairy; eggs; pulses, nuts; and other foods) consumed daily. Range from 0 to 10. | Multi-adjusted linear and logistic regression | Micronutrient status | Favorable association: DDS was positively associated with serum retinol (B=0.05) and higher DDS was associated with lower vitamin A insufficiency risk (OR: 0.64, 95%CI: 0.43, 0.95). |
| Truthmann et al. (38) | 2012 | Cross. | Germany | Male and female adolescents (12-17 y.); *n=*5198 | Semi-quantitative FFQ to estimate average food use during the last few weeks (45 food items); Self-administered; Five portion sizes illustrated by pictures | DGI: HFD Index. Multiplication of the Berry Index by the health value of the diet (which is the sum of volume share of each food multiplied by a health factor based on the German Nutrition Society). Range from 0 to 1. | Multi-adjusted linear regression | Micronutrient status | Mixed association: Mean values of serum folate increased for increasing quintiles of the HFD Index, but not mean values of serum vitamin B12, HbA1c and ferritin. |
| Rodríguez-Rodríguez et al. (104) | 2015 | Cross. | Spain | Male and female adults (18-60 y.); *n=*329 | Two consecutive quantitative 24-hr recall; Interviewer-administered; Approximate weights or household measures | FII: Diet variety. Count of different foods consumed (sub-component of the HIE) which was rescaled (less than 7 foods was 0, more than 15 foods was 10). Range from 0 to 10. | Multi-adjusted linear regression | Micronutrient status | Favorable association: Diet variety was positively associated with excretion of potassium in the urine, a biomarker of potassium dietary intake (B: 3.45, SE: 0.73). |

1 Cross., Cross-sectional; Long., Longitudinal

**Supplemental Table 5.** Summary of 17 studies evaluating the association between dietary diversity indicators and mental health and cognitive functions in participants aged 10 years and older^1^.

| Study (ref) | Year | Design | Country | Population | Dietary assessment | Dietary diversity indicator | Statistical methods | Health Outcome | Associations |
| --- | --- | --- | --- | --- | --- | --- | --- | --- | --- |
| Tangney et al. (107) | 2002 | Cross. | USA | Old female adults; *n=*117 | Quantitative FFQ to estimate average food use during the last year (100 food items); Self-administered | FII: Diet variety. Count of different foods consumed (sub-component of the HIE) which was rescaled (less than 31 foods was 0, more than 49 foods was 10). Range from 0 to 10. Minimum reported amount for inclusion (once per month). | Mann-Whitney U and Kruskal-Wallis tests | Mental health | Favorable association: Inverse relationship between the variety score and depression score (r=-0.19). |
| Kimura et al. (43) | 2009 | Cross. | China | Male and female adults (≥60 y.); *n=*240 | FFQ to estimate average food use during the last week (11 food items); Interviewer-administered | FGI: FDSK-11. Count of eleven food groups (grain; meat; fish and shellfish; eggs; milk; beans and bean products; potatoes; vegetables; seaweed; nuts; and fruits) consumed. Range from 0 to 11. | T-test | Mental health | Null association: No association was found between FDSK-11 and depression scale. |
| Milte et al. (110) | 2015 | Long. | Australia | Male and female adults (55-65 y.); *n=*2457 | FFQ to estimate average food use during the past 6 months (111 food items); Self-administered | FII: RFS. Count of recommended food items consumed. Range from 0 to 49. Minimum reported amount for inclusion (once a week). | Multi-adjusted logistic regression | Mental health | Mixed association: Higher RFS was associated with better emotional wellbeing in women (OR: 1.41, 95%CI: 1.12, 1.77) but not in men. |
| Perez-Cornago et al. (109) | 2015 | Cross. | Spain | Male and female adults; *n=*84 | Two consecutive days quantitative food record; Interviewer-administered; Weighted | FII: Dietary variety. Count of different food items consumed daily. | ANCOVA | Mental health | Favorable association: Adults in the highest mood state reported a significant greater dietary variety than their counterparts. |
| Poorrezaeian et al. (108) | 2015 | Cross. | Iran | Female adults (20-49 y.); *n=*360 | One quantitative 24-hr recall; Interviewer-administered | FGI: Dietary diversity score. Count of nine food groups (cereals and white roots; milk and dairy products; vitamin A-rich vegetable and fruits; green leafy vegetables; other vegetable and fruits; meat, fish and seafood; organ meat; eggs; nut, seeds and legumes) consumed daily. Range from 0 to 9. Minimum reported amount for inclusion (half-serving). | ANCOVA | Mental health | Favorable association: Mean anxiety score was lower in women who had high Dietary diversity score compared to those with low Dietary diversity score. |
| Poorrezaeian et al. (111) | 2017 | Cross. | Iran | Female adults (20-49 y.); *n=*360 | One quantitative 24-hr recall; Interviewer-administered | FGI: DDS. Count of nine food groups (cereals and white roots; milk and dairy products; vitamin A-rich vegetable and fruits; green leafy vegetables; other vegetable and fruits; meat, fish and seafood; organ meat; eggs; nut, seeds and legumes) consumed daily. Range from 0 to 9. Minimum reported amount for inclusion (half-serving). | Multi-adjusted logistic regression | Mental health | Mixed association: Women with high DDS had lower odds of severe depression (OR: 0.61, 95%CI: 0.40, 0·91) compared with those with low DDS. DDS was not associated with stress status. |
| Masa et al. (11) | 2018 | Cross. | Zambia | Female adults (18-50 y.); *n=*101 | FFQ to estimate average food use during past week (6 food items); Interviewer-administered | OI: IDD. Count of six food groups (staples; meat; fish; beans; seeds and nuts; and vegetables) consumed over 6 months, frequency of consumption (0 = never; 1 = rarely; 2 = sometimes; and 3 = often) and weighting to confer more importance to foods of animal origin (weight = 4), then beans and nuts (weight = 3), staples (weight = 2), and vegetables (weight = 1). Range from 0 to 51. | Multi-adjusted linear regression | Mental health | Null association: IDD was not significantly associated with perceived stress. |
| Pomer et al. (112) | 2018 | Long. | Vanuatu | Pregnant female adults (15-49 y.); *n=*70 | One 24-hr recall; Self-administered | FGI: WDDS. Count of nine food groups consumed daily. Range from 0 to 9. | Multi-adjusted linear regression | Mental health | Null association: WDDS was not associated with psychosocial distress during pregnancy. |
| Clausen et al. (42) | 2005 | Cross. | Botswana | Male and female adults (≥60 y.); *n=*372 | Qualitative FFQ to estimate average food use during an unknown period (21 food items); Interviewer-administered | FII: FVS. Count of the frequency scores (0 to 7) of 16 selected food consumed. Range from 0 to 112. Minimum reported amount for inclusion (once a week). | T-test | Cognitive function | Favorable association: FVS was higher in old adults with higher cognitive functioning level and memory test. |
| Chen et al. (113) | 2011 | Long. | Taiwan | Male and female adults (65-97 y.); *n=*1839 | One quantitative 24-hr recall; Interviewer-administered | FGI: DDS. Count of six food groups (dairy; eggs, legumes, fish, meat; grain; fruit; vegetable; and oil, fat) consumed daily. Range from 0 to 6. Minimum reported amount for inclusion (half-serving). | Multi-adjusted Cox's proportional-hazards regression | Cognitive function | Favorable association: Severe cognitive impairment was associated with lower DDS |
| Tomioka et al. (114) | 2015 | Cross. | Japan | Male and female adults (65-100 y.); *n=*8910 | FFQ to estimate average food use during the last week (10 food items); Self-administered | FGI: DVS. Count of ten food groups (meat; fish and shellfish; eggs; milk; soybean products; potatoes; green and yellow vegetables; fruits; seaweed; and fat and oil) consumed. Range from 0 to 10. Minimum reported amount for inclusion (almost once a day). | Multi-adjusted logistic regression | Cognitive function | Favorable association: Poor intellectual activity was associated with the lowest DVS quartile (OR: 1.86, 95%CI: 1.61, 2.15) compared to the highest DVS quartile. |
| Otsuka et al. (4) | 2016 | Long. | Japan | Male and female adults (40-79 y.); *n=*1317 | Three consecutive days quantitative food record (2 weekdays and 1 weekend day); Self-administered; Weighted | OI: QUANTIDD. Calculated by the proportion of foods that contribute to total energy (or amount) and the number of food groups among 17. Range from 0 to 1. | Multi-adjusted logistic regression | Cognitive function | Favorable association: Increasing QUANTIDD score was associated with lower risk of decline in intellectual activity (OR: 0.47, 95%CI: 0.23, 0.95 when Q2 vs Q1, OR: 0.44, 95%CI: 0.22, 0.90) when Q3 vs Q1; OR: 0.41, 95%CI: 0.20, 0.83 when Q4 vs Q1). |
| Otsuka et al. (5) | 2017 | Long. | Japan | Male and female adults (60-81 y.); *n=*570 | Three consecutive days quantitative food record (2 weekdays and 1 weekend day); Self-administered; Weighted | OI: QUANTIDD. Calculated by the proportion of foods that contribute to total energy (or amount) and the number of food groups among 17. Range from 0 to 1. | Multi-adjusted linear regression | Cognitive function | Favorable association: Increasing QUANTIDD score was associated with lower risk of decline in intellectual activity (OR: 0.68, 95%CI: 0.46, 0.99) when Q3 vs Q1; OR: 0.56, 95%CI: 0.38, 0.83 when Q4 vs Q1). |
| Yin et al. (115) | 2017 | Cross. | China | Male and female adults (≥65 y.); *n=*8571 | Qualitative FFQ to estimate average food use during an unknown period; Self-administered | FGI: DDS. Count of nine food groups (vegetables; fruits; legumes and its products; nuts; meat; eggs; fish; milk and dairy products; tea) consumed. Range from 0 to 9. Minimum reported amount for inclusion (once per week). | Multi-adjusted linear and logistic regression | Cognitive function | Favorable association: Lower DDS was associated with decreased cognitive function (B: −0.11, 95%CI: -0.14, -0.08) and higher odds of cognitive impairment (OR = 1.29, 95%CI: 1.14, 1.47, p<0.01). |
| Schebendach et al. (116) | 2008 | Long. | USA | Female adult (18-45 y.); *n=*41 | Four days quantitative food record; Self-administered; Instructions to estimate serving sizes and pictorial examples of food portions | FII: DVS. Count of different foods consumed divided by the total number of food record days. | Multi-adjusted linear regression | Anorexia nervosa treatment | Favorable association: Higher DVS was associated with successful anorexia nervosa treatment. |
| Schebendach et al. (117) | 2011 | Long. | USA | Female adult (18-45 y.); *n=*41 | Four days quantitative food record; Self-administered; Instructions to estimate serving sizes and pictorial examples of food portions | FII: Total diet variety. Count of different foods consumed divided by the total number of food record days. | T-test | Anorexia nervosa treatment | Favorable association: Success group in anorexia nervosa treatment had higher total diet variety than failure group. |
| Schebendach et al. (118) | 2012 | Long. | USA | Female adult (18-45 y.); *n=*16 | Four days quantitative food record; Self-administered; Instructions to estimate serving sizes and pictorial examples of food portions | FII: DVS. Count of different foods consumed divided by the total number of food record days. | T-test | Anorexia nervosa treatment | Null association: DVS did not differ between failure group and success group in anorexia nervosa treatment. |

1 Cross., Cross-sectional; Long., Longitudinal

**Supplemental Table 6.** Summary of 10 studies evaluating the association between dietary diversity indicators and mortality in participants aged 10 years and older^1^.

| Study (ref) | Year | Design | Country | Population | Dietary assessment | Dietary diversity indicator | Statistical methods | Associations |
| --- | --- | --- | --- | --- | --- | --- | --- | --- |
| Kant et al. (133) | 1993 | Long. | USA | Male and female adults (25-74 y.); *n=*10424 | One quantitative 24-hr recall; Interviewer-administered; 3D food models | FGI: DDS. Count of five food groups (dairy; meat; grain; fruit; and vegetable) consumed daily. Range from 0 to 5. Minimum reported amount for inclusion (15 g for solids or 30 g for liquids and mixed dishes when dairy, 30 g or 60 g when other groups). | Multi-adjusted Cox's proportional-hazards regression | Favorable association: Relative risk of mortality was inversely related to DDS in men (RR: 1.5, 95%CI:1.2, 1.8) and women (RR: 1.4, 95%CI: 1.1, 1.9). |
| Kant et al. (134) | 1995 | Long. | USA | Male and female adults (25-74 y.); *n=*10337 | One quantitative 24-h recall; Interviewer-administered; 3D food models | FGI: DDS. Count of five food groups (dairy; meat; grain; fruit; and vegetable) consumed daily. Range from 0 to 5. Minimum reported amount for inclusion (15 g for solids or 30 g for liquids and mixed dishes when dairy, 30 g or 60 g when other groups). | Multi-adjusted Cox's proportional-hazards regression | Null association: Age-adjusted death rates from cardiovascular disease, cancer, and other causes of mortality were inversely associated with DDS. After adjustment for multiple covariates, the associations were no longer significant. |
| Chen et al. (113) | 2011 | Long. | Taiwan | Male and female adults (65-94 y.); *n=*1839 | One quantitative 24-hr recall; Interviewer-administered | FGI: DDS. Count of six food groups (dairy; eggs, legumes, fish, meat; grain; fruit; vegetable; and oil, fat) consumed daily. Range from 0 to 6. Minimum reported amount for inclusion (half-serving). | Multi-adjusted Cox's proportional-hazards regression | Favorable association: Compared to normal cognitive function and highest DDS, the greatest HR was where impaired cognition was combined with the lowest DDS (HR: 2.24, 95%CI: 1.19, 4.24). Attributability for mortality amounted to 18% for impaired cognition and 33% for less diverse diet. |
| Lee et al. (47) | 2011 | Long. | Taiwan | Male and female adults (≥ 65 y.); *n=*1743 | One quantitative 24-hr recall; Interviewer-administered | FGI: DDS. Count of six food groups (dairy; eggs, legumes, fish, meat; grain; fruit; vegetable; and oil, fat) consumed daily. Range from 0 to 6. Minimum reported amount for inclusion (half-serving). | Multi-adjusted Cox's proportional-hazards regression | Mixed association: Risks of all-cause, cancer and diabetes mortality decreased as the DDS increased while but not risks of circulatory disease, cerebrovascular disease and pneumonia mortality) were not associated with DDS. |
| Rawat et al. (102) | 2013 | Long. | Uganda | Male and female adults; *n=*876 | One 24-hr recall; Interviewer-administered | FGI: IDDS. Count of twelve food groups (cereals; roots and tubers; pulse, legumes, nuts; vegetables; fruits; meat and poultry; eggs; fish and seafood; milk and milk products; oils and fats; sugar and sweets; and condiments and miscellaneous). Range from 0 to 12. | Multi-adjusted linear and logistic regression | Favorable association: IDDS was associated with lower mortality in both unadjusted (HR: 0.80; 95%CI: 0.69, 0.93) and adjusted models (HR: 0.79, 95%CI: 0.65, 0.94). |
| Huang et al. (158) | 2014 | Long. | Taiwan | Male and female adults (65-97 y.); *n=*1856 | One quantitative 24-h recall; Interviewer-administered; | FGI: DDS. Count of six food groups (dairy; eggs, legumes, fish, meat; grain; fruit; vegetable; and oil, fat) consumed daily. Range from 0 to 6. Minimum reported amount for inclusion (half-serving). | Multi-adjusted Cox's proportional-hazards regression | Favorable association: Adult with lower DDS and poor appetites had higher risk of mortality (HR: 1.77, 95%CI: 1.04, 3.00) compared to those with higher DDS and good appetites. |
| Huang et al. (159) | 2015 | Long. | Taiwan | Male and female adults (65-97 y.); *n=*1400 | One quantitative 24-h recall; Interviewer-administered; | FGI: DDS. Count of six food groups (dairy; eggs, legumes, fish, meat; grain; fruit; vegetable; and oil, fat) consumed daily. Range from 0 to 6. Minimum reported amount for inclusion (half-serving). | Multi-adjusted Cox's proportional-hazards regression | Favorable association: Adult with moderate magnesium intake and higher DDS (>4) had lower risk of mortality (HR: 0.57, 95%CI:0.44, 0.74 when Mg intake in Q2 and HR: 0.59, 95%CI: 0.39, 0.88 when Mg intake in Q3) compared with participants with the lowest Mg intake and DDS (≤4). |
| Masset et al. (85) | 2015 | Long. | UK | Male and female adults (35-65 y.); *n=*7251 | Quantitative FFQ to estimate average food use during the last year (127 food items); Self-administered; Common unit or portion size | FII:  - FVS: Count of food items reported to be consumed. Minimum reported amount for inclusion (once a week).  - RFVOfcom and RFV(SAIN,LIM): Count of foods identified as ‘healthy’ by the Ofcom and SAIN,LIM models. Minimum reported amount for inclusion (once a week).  - NRFVOfcom and NRFV(SAIN,LIM): Count of foods identified as ‘less healthy’ aby the Ofcom and SAIN,LIM models (the higher, the worst). Minimum reported amount for inclusion (once a week). | Multi-adjusted Cox's proportional-hazards regression | Mixed association: FVS (fourth v. first quartile) and RFVOfcom (third v. first quartile) were associated with a 26% and 27% reduction all-cause mortality risk while RFV(SAIN,LIM), NRFV(Ofcom) and NRFV(SAIN,LIM) were not associated. Only FVS (fourth v. first quartile) was associated with 39% reduction of CHD mortality risk. Only RFVOfcom (third v. first quartile) was associated with a 35% cancer mortality risk. None DDI was associated with reduction of CVD mortality risk. |
| Letois et al. (160) | 2016 | Long. | France | Male and female adults (≥ 65 y.); *n=*8937 | Semi-quantitative FFQ to estimate average food use during an unknown period (9 food items); Interviewer-administered | FGI: DDS. Count of five food groups (dairy; meat; grain; fruit; and vegetable) consumed daily. Range from 0 to 5. Minimum reported amount for inclusion (once per day). | Multi-adjusted Cox's proportional-hazards regression | Null association: Higher DDS was associated with better survival in crude analysis but not in adjusted analysis (OR: 0.94, 95%CI: 0.85, 1.04). |
| Wahlqvis et al. (161) | 2016 | Long. | Taiwan | Male and female adults (65-754 y.); *n=*1412 | One 24-hr recall; Interviewer-administered | FGI: DDS. Count of six food groups (dairy; eggs, legumes, fish, meat; grain; fruit; vegetable; and oil, fat) consumed daily. Range from 0 to 6. Minimum reported amount for inclusion (half-serving). | Multi-adjusted Cox's proportional-hazards regression | Mixed association: Higher DDS reduced mortality risk of homocysteinaemia in elderly Taiwanese (HR: 1.76, 95%CI: 1.29, 2.41) , but not in those who have diabetes. |

1 Long., Longitudinal

**Supplemental Table 7.** Summary of 18 studies evaluating the association between dietary diversity indicators and other health outcomes in participants aged 10 years and older^1^.

| Study (ref) | Year | Design | Country | Population | Dietary assessment | Dietary diversity indicator | Statistical methods | Health Outcome | Associations |
| --- | --- | --- | --- | --- | --- | --- | --- | --- | --- |
| Abubakari et al. (31) | 2016 | Cross. | Ghana | Pregnant female adult (≥16 y.); *n=*578 | FFQ to estimate average food use since women became pregnant or were pregnant (55 food items); Interviewer-administered | FGI: WDDS. Count of nine food groups (starchy staples and white roots; vitamin A-rich fruits and vegetables; dark green leafy vegetables; other fruits and vegetables; organ meat; meat and fish; eggs; legumes, nuts and seeds; and milk and milk products) consumed daily. Range from 0 to 9. | Univariate logistic regression | Pregnancy and birth | Favorable association: Higher DDS showed a reduced risk of low weight birth (OR: 0.10, 95%CI: 0.04, 0.13) |
| Zerfu et al. (93) | 2016 | Long. | Ethiopia | Pregnant female adult; *n=*374 | Four non-consecutive 24-hr recalls collected each month from enrollment to delivery; Interviewer-administered | FGI: WDDS. Count of nine food groups (cereals, roots and tubers; vitamin A–rich fruit and vegetables; other fruit; other vegetables; legumes and nuts; meat, poultry, and fish; fats and oils; dairy; and eggs) consumed over the 4 days. Range from 0 to 9. | Multi-adjusted logistic regression | Pregnancy and birth | Favorable association: Women in the inadequate WDDS group had higher risk of preterm birth (RR: 4.61, 95%CI: 2.31, 9.19), higher risk of low birth weight (RR: 2.06, 95%CI: 1.03, 4.11) and tended to have higher risk of stillbirth (RR: 2.71, 95%CI: 0.88, 8.36). |
| Manerkar et al. (119) | 2017 | Long. | India | Pregnant female adult (19-35 y.); *n=*121 | One 24-hr recall; Interviewer-administered | FGI: Diet diversity score. Count of nine food groups consumed daily. Range from 0 to 9. | Chi2 | Pregnancy and birth | Null association: Diet diversity score was not associated with birth weight. |
| Pomer et al. (112) | 2018 | Long. | Vanuatu | Pregnant female adult (19-49 y.); *n=*70 | One 24-hr recall; Self-administered | FGI: WDDS. Count of nine food groups consumed daily. Range from 0 to 9. | Multi-adjusted linear regression | Pregnancy and birth | Null association: WDDS was not associated with birth weight. |
| Wagenaar et al. (122) | 2015 | Case. | Bangladesh | Male and female adult (18-50 y.); *n=*152 | One qualitative 24-hr recall; Interviewer-administered | FGI: DDS. Count of nine food groups (starchy staples; dark green leafy vegetables; other vitamin A rich fruits and vegetables; other fruits and vegetables; organ meat; meat and fish; eggs; legumes, nuts and seeds; and milk and milk products) consumed daily. Range from 0 to 9. | Multi-adjusted logistic regression | Leprosy | Null association: IDDS was not associated with leprosy. |
| Oktaria et al. (123) | 2018 | Case. | Indonesia | Male and female adult (16-65 y.); *n=*300 | One qualitative 24-hr recall; Interviewer-administered | FGI: IDDS. Count of nine food groups (starchy staples and white roots; vitamin A-rich fruits and vegetables; dark green leafy vegetables; other fruits and vegetables; organ meat; meat and fish; eggs; legumes, nuts and seeds; and milk and milk products) consumed daily. Range from 0 to 9. | Multi-adjusted logistic regression | Leprosy | Null association: IDDS was not associated with leprosy. |
| Yokoyama et al. (120) | 2017 | Long. | Japan | Male and female adult (≥65 y.); *n=*779 | FFQ to estimate average food use during an unknown period (10 food items); Interviewer-administered | FGI: DVS. Count of ten food groups (meat; fish and shellfish; eggs; milk; soybean products; potatoes; green and yellow vegetables; fruits; seaweed; and fat and oil) consumed. Range from 0 to 10. Minimum reported amount for inclusion (almost once a day). | Multi-adjusted logistic regression | Sarcopenia | Mixed association: Higher DDS was associated with lower risk for future declines in physical performance (muscle strength (OR: 0.43, 95%CI: 0.19, 0.99) and gait speed (OR: 0.43, 95%CI: 0.19, 0.99)) but was not associated with lean body mass and appendicular lean mass. |
| Momoki et al. (121) | 2017 | Cross. | Japan | Female adult (≥65 y.); *n=*186 | Unknown | FGI: DVS. Count of unknown food groups consumed. | Multi-adjusted logistic regression | Sarcopenia | Unfavorable association: Higher DVS (≥9) tended to be associated with higher sarcopenia risk (OR: 4.98, 95%CI 0.97, 25.56) |
| Tangney et al. (107) | 2002 | Cross. | USA | Old female adult; *n=*117 | Quantitative FFQ to estimate average food use during the last year (100 food items); Self-administered | FII: Diet variety. Count of different foods consumed (sub-component of the HIE) which was rescaled (less than 31 foods was 0, more than 49 foods was 10). Range from 0 to 10. Minimum reported amount for inclusion (once per month). | Mann-Whitney U and Kruskal-Wallis tests | Symptomatology of breast cancer | Favorable association: Higher variety score was associated with less advanced stages of breast cancer and negative node status |
| Ghadirian et al. (125) | 2009 | Cross. | Canada | Old female adult; *n=*739 | Quantitative FFQ to estimate average food use during the last year prior to the diagnosis of cancer or hospital admission (164 food items); Interviewer-administered | FII: Diet diversity. Count of different food items consumed. Minimum reported amount for inclusion (once a week). | Multi-adjusted logistic regression | Combination of BRCA mutation | Null association: Diet diversity was not associated with BRCA mutations. |
| Lim et al. (131) | 2010 | Cross. | South Korea | Old male and female adult; *n=*73 | Unknown | FII: DVS. Count of different foods consumed daily. Minimum reported amount for inclusion (15 g for solids or 30 g for liquids and mixed dishes when dairy, 30 g or 60 g when other groups).  FGI: DDS. Count of five food groups (dairy; meat; grain; fruit; and vegetable) consumed daily. Range from 0 to 5. Minimum reported amount for inclusion (15 g for solids or 30 g for liquids and mixed dishes when dairy, 30 g or 60 g when other groups). | Pearson’s correlation | Nutritional status assessed by PG-SGA | Favorable association: DVS and DDS were negatively correlated with nutritional status assess by PG-SGA (r=-0.693 and r=-0.649, respectively) |
| Whittle et al. (124) | 2012 | Long. | UK | Male and female adult (20-25 y.); *n=*489 | Diet history questionnaire with a referent period of seven days; Interviewer-administered; Photographs of servings, common household cups, glasses and dishes | FGI: DDS. Count of five food groups (dairy; meat; grain; fruit; and vegetable) consumed daily and average over the 7 days. Range from 0 to 5. Minimum reported amount for inclusion (15 g for solids or 30 g for liquids and mixed dishes when dairy, 30 g or 60 g when other groups). | Multi-adjusted linear regression | Bone mineral status | Null association: DDS was not associated with bone mineral content and density. |
| Erickson et al. (130) | 2012 | Cross. | USA | Male and female adult (≥40 y.); *n=*1385 | One quantitative 24-hr recall; Interviewer-administered | FII: Dietary variety. Count of different foods consumed (sub-component of the HIE) which was rescaled (less than 4 foods was 0, more than 9 foods was 10). Range from 0 to 10. | Multi-adjusted logistic regression | Lower Urinary Tract Symptoms | Null association: Dietary variety was not associated with and Lower Urinary Tract Symptoms. |
| Abebe et al. (126) | 2014 | Cross. | Ethiopia | Pregnant female adult (15-49 y.); *n=*104 | One 24-hr recall; Interviewer-administered | FGI: DDS. Count of unknown food groups consumed daily. Minimum reported amount for inclusion (1 tablespoon of food). | Univariate linear regression | Dark adaptation threshold | Favorable association: Higher DDS was associated with decrease in dark adaptation measurement (B: -0.29, 95%CI: -0.75, -0.10). |
| Saito et al. (128) | 2014 | Long. | Japan | Male and female adult (≥70 y.); *n=*1084 | FFQ to estimate average food use during an unknown period (10 food items); Interviewer-administered | FGI: DVS. Count of ten food groups (meat; fish and shellfish; eggs; milk; soybean products; potatoes; green and yellow vegetables; fruits; seaweed; and fat and oil) consumed. Range from 0 to 10. Minimum reported amount for inclusion (almost once a day). | Multi-adjusted Cox's proportional-hazards regression | Functional disability | Favorable association: Lower DVS (<9) was associated with functional disability (HR: 5.30; 95%CI:1.34, 20.94). |
| Shiraseb et al. (132) | 2016 | Cross. | Iran | Male and female adult (20-50 y.); *n=*400 | One quantitative 24-hr recall; Interviewer-administered; Common household utensils | FGI: DDS. Count of nine food groups (starchy staples and white roots; vitamin A-rich fruits and vegetables; dark green leafy vegetables; other fruits and vegetables; organ meat; meat and fish; eggs; legumes, nuts and seeds; and milk and milk products) consumed daily. Range from 0 to 9. Minimum reported amount for inclusion (half-serving). | ANCOVA | Visual and auditory sustained attention | Favorable association: Mean subscales of attention (auditory consistency and vigilance, visual persistence, visual and auditory focus, speed, comprehension and full attention) increased as the quartiles of DDS increased. |
| Motokawa et al. (127) | 2018 | Cross. | Japan | Male and female adult (65-84 y.); *n=*400 | Diet history questionnaire with a referent period of a month (but a period of one week was used to calculate DVS); Self-administered | FGI: DVS. Count of ten food groups (meat; fish and shellfish; eggs; milk; soybean products; potatoes; green and yellow vegetables; fruits; seaweed; and fat and oil) consumed. Range from 0 to 10. Minimum reported amount for inclusion (almost once a day). | Univariate logistic regression | Frailty severity | Favorable association: Higher DVS was associated with lower frailty severity (B: -0.102, 95%CI: -0.190, -0.014). |

1 Case., Case-control; Cross., Cross-sectional; Long., Longitudinal

**Supplemental References**

1. Drescher LS, Thiele S, Mensink GBM. A new index to measure healthy food diversity better reflects a healthy diet than traditional measures. J Nutr. 2007 Mar;137(3):647–51.

2. Otto MC de O, Padhye NS, Bertoni AG, Jacobs DR, Mozaffarian D. Everything in Moderation--Dietary Diversity and Quality, Central Obesity and Risk of Diabetes. PloS One. 2015;10(10):e0141341.

3. Lachat C, Raneri JE, Smith KW, Kolsteren P, Van Damme P, Verzelen K, et al. Dietary species richness as a measure of food biodiversity and nutritional quality of diets. Proc Natl Acad Sci U S A. 2018 Jan 2;115(1):127–32.

4. Otsuka R, Kato Y, Nishita Y, Tange C, Nakamoto M, Tomida M, et al. Dietary diversity and 14-year decline in higher-level functional capacity among middle-aged and elderly Japanese. Nutr Burbank Los Angel Cty Calif. 2016 Aug;32(7–8):784–9.

5. Otsuka R, Nishita Y, Tange C, Tomida M, Kato Y, Nakamoto M, et al. Dietary diversity decreases the risk of cognitive decline among Japanese older adults. Geriatr Gerontol Int. 2017 Jun;17(6):937–44.

6. Tian X, Wu M, Zang J, Zhu Y, Wang H. Dietary diversity and adiposity in Chinese men and women: an analysis of four waves of cross-sectional survey data. Eur J Clin Nutr. 2017;71(4):506–11.

7. Woo J, Cheung B, Ho S, Sham A, Lam TH. Influence of dietary pattern on the development of overweight in a Chinese population. Eur J Clin Nutr. 2008 Apr;62(4):480–7.

8. Sea MM-M, Woo J, Tong PC-Y, Chow C-C, Chan JC-N. Associations between food variety and body fatness in Hong Kong Chinese adults. J Am Coll Nutr. 2004 Oct;23(5):404–13.

9. Woo J, Ho SC, Sham A, Sea MM, Lam KSL, Lam TH, et al. Diet and glucose tolerance in a Chinese population. Eur J Clin Nutr. 2003 Apr;57(4):523–30.

10. McCrory MA, Fuss PJ, McCallum JE, Yao M, Vinken AG, Hays NP, et al. Dietary variety within food groups: association with energy intake and body fatness in men and women. Am J Clin Nutr. 1999 Mar;69(3):440–7.

11. Masa R, Chowa G, Nyirenda V. Socioeconomic correlates of dietary diversity and its association with adherence and psychosocial functioning of people living with HIV in rural Zambia. Nutr Health. 2018 Jan 1;260106018761282.

12. Benefice E, Lopez R, Monroy SL, Rodríguez S. Fatness and overweight in women and children from riverine Amerindian communities of the Beni River (Bolivian Amazon). Am J Hum Biol Off J Hum Biol Counc. 2007 Feb;19(1):61–73.

13. Roberts SB, Hajduk CL, Howarth NC, Russell R, McCrory MA. Dietary variety predicts low body mass index and inadequate macronutrient and micronutrient intakes in community-dwelling older adults. J Gerontol A Biol Sci Med Sci. 2005 May;60(5):613–21.

14. Gregory CO, McCullough ML, Ramirez-Zea M, Stein AD. Diet scores and cardio-metabolic risk factors among Guatemalan young adults. Br J Nutr. 2009 Jun;101(12):1805–11.

15. Kant AK, Graubard BI. A comparison of three dietary pattern indexes for predicting biomarkers of diet and disease. J Am Coll Nutr. 2005 Aug;24(4):294–303.

16. Mirmiran P, Azadbakht L, Esmaillzadeh A, Azizi F. Dietary diversity score in adolescents - a good indicator of the nutritional adequacy of diets: Tehran lipid and glucose study. Asia Pac J Clin Nutr. 2004;13(1):56–60.

17. Azadbakht L, Akbari F, Esmaillzadeh A. Diet quality among Iranian adolescents needs improvement. Public Health Nutr. 2015 Mar;18(4):615–21.

18. Vadiveloo M, Parekh N, Parkeh N, Mattei J. Greater healthful food variety as measured by the US Healthy Food Diversity index is associated with lower odds of metabolic syndrome and its components in US adults. J Nutr. 2015 Mar;145(3):564–71.

19. Vadiveloo M, Dixon LB, Mijanovich T, Elbel B, Parekh N. Dietary variety is inversely associated with body adiposity among US adults using a novel food diversity index. J Nutr. 2015 Mar;145(3):555–63.

20. Vadiveloo M, Sacks FM, Champagne CM, Bray GA, Mattei J. Greater Healthful Dietary Variety Is Associated with Greater 2-Year Changes in Weight and Adiposity in the Preventing Overweight Using Novel Dietary Strategies (POUNDS Lost) Trial. J Nutr. 2016;146(8):1552–9.

21. Shariff ZM, Khor GL. Obesity and household food insecurity: evidence from a sample of rural households in Malaysia. Eur J Clin Nutr. 2005 Sep;59(9):1049–58.

22. Azadbakht L, Esmaillzadeh A. Dietary diversity score is related to obesity and abdominal adiposity among Iranian female youth. Public Health Nutr. 2011 Jan;14(1):62–9.

23. Florêncio TMMT, Bueno NB, Clemente APG, Albuquerque FCA, Britto RPA, Ferriolli E, et al. Weight gain and reduced energy expenditure in low-income Brazilian women living in slums: a 4-year follow-up study. Br J Nutr. 2015 Aug 14;114(3):462–71.

24. Azadbakht L, Mirmiran P, Azizi F. Dietary diversity score is favorably associated with the metabolic syndrome in Tehranian adults. Int J Obes 2005. 2005 Nov;29(11):1361–7.

25. Nachvak SM, Abdollahzad H, Mostafai R, Moradi S, Pasdar Y, Rezaei M, et al. Dietary Diversity Score and Its Related Factors among Employees of Kermanshah University of Medical Sciences. Clin Nutr Res. 2017 Oct;6(4):247–55.

26. Tiew KF, Chan YM, Lye MS, Loke SC. Factors associated with dietary diversity score among individuals with type 2 diabetes mellitus. J Health Popul Nutr. 2014 Dec;32(4):665–76.

27. Bernstein MA, Tucker KL, Ryan ND, O’Neill EF, Clements KM, Nelson ME, et al. Higher dietary variety is associated with better nutritional status in frail elderly people. J Am Diet Assoc. 2002 Aug;102(8):1096–104.

28. Kent LM, Worsley A. Trends in BMI, diet and lifestyle between 1976 and 2005 in North Sydney. Asia Pac J Clin Nutr. 2009;18(3):453–61.

29. Mayega RW, Makumbi F, Rutebemberwa E, Peterson S, Östenson C-G, Tomson G, et al. Modifiable socio-behavioural factors associated with overweight and hypertension among persons aged 35 to 60 years in eastern Uganda. PloS One. 2012;7(10):e47632.

30. Ali F, Thaver I, Khan SA. Assessment of dietary diversity and nutritional status of pregnant women in Islamabad, Pakistan. J Ayub Med Coll Abbottabad JAMC. 2014 Dec;26(4):506–9.

31. Abubakari A, Jahn A. Maternal Dietary Patterns and Practices and Birth Weight in Northern Ghana. PloS One. 2016;11(9):e0162285.

32. Amugsi DA, Dimbuene ZT, Bakibinga P, Kimani-Murage EW, Haregu TN, Mberu B. Dietary diversity, socioeconomic status and maternal body mass index (BMI): quantile regression analysis of nationally representative data from Ghana, Namibia and Sao Tome and Principe. Bmj Open. 2016;6(9):e012615.

33. Haws KL, Liu PJ, Redden JP, Silver HJ. Exploring the Relationship Between Varieties of Variety and Weight Loss: When More Variety Can Help People Lose Weight. J Mark Res. 2017 Aug;54(4):619–35.

34. Ishikawa M, Moriya S, Yokoyama T. Relationship between diet-related indicators and overweight and obesity in older adults in rural Japan. J Nutr Health Aging. 2017;21(7):759–65.

35. Tsuchiya C, Tagini S, Cafa D, Nakazawa M. Socio-environmental and behavioral risk factors associated with obesity in the capital (Honiara), the Solomon Islands; case-control study. Obes Med. 2017 Sep;7:34–42.

36. Kant AK, Thompson FE. Measures of overall diet quality from a food frequency questionnaire: National Health Interview Survey, 1992. Nutr Res. 1997 Sep;17(9):1443–56.

37. Tavakoli S, Dorosty-Motlagh AR, Hoshiar-Rad A, Eshraghian MR, Sotoudeh G, Azadbakht L, et al. Is dietary diversity a proxy measurement of nutrient adequacy in Iranian elderly women? Appetite. 2016 01;105:468–76.

38. Truthmann J, Richter A, Thiele S, Drescher L, Roosen J, Mensink GB. Associations of dietary indices with biomarkers of dietary exposure and cardiovascular status among adolescents in Germany. Nutr Metab. 2012 Oct 24;9:92.

39. Hsu-Hage BH, Wahlqvist ML. Food variety of adult Melbourne Chinese: a case study of a population in transition. World Rev Nutr Diet. 1996;79:53–69.

40. Vandevijvere S, De Vriese S, Huybrechts I, Moreau M, Van Oyen H. Overall and within-food group diversity are associated with dietary quality in Belgium. Public Health Nutr. 2010 Dec;13(12):1965–73.

41. Slattery ML, Berry TD, Potter J, Caan B. Diet diversity, diet composition, and risk of colon cancer (United States). Cancer Causes Control CCC. 1997 Nov;8(6):872–82.

42. Clausen T, Charlton KE, Gobotswang KSM, Holmboe-Ottesen G. Predictors of food variety and dietary diversity among older persons in Botswana. Nutr Burbank Los Angel Cty Calif. 2005 Jan;21(1):86–95.

43. Kimura Y, Okumiya K, Sakamoto R, Ishine M, Wada T, Kosaka Y, et al. Comprehensive geriatric assessment of elderly highlanders in Qinghai, China IV: Comparison of food diversity and its relation to health of Han and Tibetan elderly. Geriatr Gerontol Int. 2009 Dec;9(4):359–65.

44. Saibul N, Shariff ZM, Lin KG, Kandiah M, Ghani NA, Rahman HA. Food variety score is associated with dual burden of malnutrition in Orang Asli (Malaysian indigenous peoples) households: implications for health promotion. Asia Pac J Clin Nutr. 2009;18(3):412–22.

45. Haemamalar K, Zalilah MS, Neng Azhanie A. Nutritional status of orang asli (che wong tribe) adults in krau wildlife reserve, pahang. Malays J Nutr. 2010 Apr;16(1):55–68.

46. Jovanović GK, Zezelj SP, Malatestinić D, Sutić IM, Stefanac VN, Dorcić F. Diet quality of middle age and older women from Primorsko-Goranska County evaluated by healthy eating index and association with body mass index. Coll Antropol. 2010 Apr;34 Suppl 2:155–60.

47. Lee M-S, Huang Y-C, Su H-H, Lee M-Z, Wahlqvist ML. A simple food quality index predicts mortality in elderly Taiwanese. J Nutr Health Aging. 2011 Dec;15(10):815–21.

48. Vakili M, Abedi P, Sharifi M, Hosseini M. Dietary diversity and its related factors among adolescents: a survey in Ahvaz-Iran. Glob J Health Sci. 2013 Jan 13;5(2):181–6.

49. Gali N, Tamiru D, Tamrat M. The Emerging Nutritional Problems of School Adolescents: Overweight/Obesity and Associated Factors in Jimma Town, Ethiopia. J Pediatr Nurs. 2017 Aug;35:98–104.

50. Azadbakht L, Mirmiran P, Esmaillzadeh A, Azizi F. Dietary diversity score and cardiovascular risk factors in Tehranian adults. Public Health Nutr. 2006 Sep;9(6):728–36.

51. Farhangi MA, Jahangiry L. Dietary diversity score is associated with cardiovascular risk factors and serum adiponectin concentrations in patients with metabolic syndrome. BMC Cardiovasc Disord. 2018 Apr 17;18(1):68.

52. Karimbeiki R, Pourmasoumi M, Feizi A, Abbasi B, Hadi A, Rafie N, et al. Higher dietary diversity score is associated with obesity: a case-control study. Public Health. 2018 Apr;157:127–34.

53. Jayawardena R, Byrne NM, Soares MJ, Katulanda P, Yadav B, Hills AP. High dietary diversity is associated with obesity in Sri Lankan adults: an evaluation of three dietary scores. BMC Public Health. 2013 Apr 8;13:314.

54. Ponce X, Ramirez E, Delisle H. A more diversified diet among Mexican men may also be more atherogenic. J Nutr. 2006 Nov;136(11):2921–7.

55. Keding GB, Msuya JM, Maass BL, Krawinkel MB. Obesity as a public health problem among adult women in rural Tanzania. Glob Health Sci Pract. 2013 Nov;1(3):359–71.

56. Yao M, McCrory MA, Ma G, Tucker KL, Gao S, Fuss P, et al. Relative influence of diet and physical activity on body composition in urban Chinese adults. Am J Clin Nutr. 2003 Jun;77(6):1409–16.

57. Zhang Q, Chen X, Liu Z, Varma DS, Wan R, Zhao S. Diet diversity and nutritional status among adults in southwest China. PloS One. 2017;12(2):e0172406.

58. Hadgu TH, Worku W, Tetemke D, Berhe H. Undernutrition among HIV positive women in Humera hospital, Tigray, Ethiopia, 2013: antiretroviral therapy alone is not enough, cross sectional study. BMC Public Health. 2013 Oct 9;13:943.

59. Savy M, Martin-Prével Y, Sawadogo P, Kameli Y, Delpeuch F. Use of variety/diversity scores for diet quality measurement: relation with nutritional status of women in a rural area in Burkina Faso. Eur J Clin Nutr. 2005 May;59(5):703–16.

60. Savy M, Martin-Prével Y, Traissac P, Eymard-Duvernay S, Delpeuch F. Dietary diversity scores and nutritional status of women change during the seasonal food shortage in rural Burkina Faso. J Nutr. 2006 Oct;136(10):2625–32.

61. Kadiyala S, Rawat R. Food access and diet quality independently predict nutritional status among people living with HIV in Uganda. Public Health Nutr. 2013 Jan;16(1):164–70.

62. Savy M, Martin-Prével Y, Traissac P, Delpeuch F. Measuring dietary diversity in rural Burkina Faso: comparison of a 1-day and a 3-day dietary recall. Public Health Nutr. 2007 Jan;10(1):71–8.

63. Nithya DJ, Bhavani RV. Dietary diversity and its relationship with nutritional status among adolescents and adults in rural India. J Biosoc Sci. 2018 May;50(3):397–413.

64. Savy M, Martin-Prével Y, Danel P, Traissac P, Dabiré H, Delpeuch F. Are dietary diversity scores related to the socio-economic and anthropometric status of women living in an urban area in Burkina Faso? Public Health Nutr. 2008 Feb;11(2):132–41.

65. Benzekri NA, Sambou J, Diaw B, Sall EHI, Sall F, Niang A, et al. High Prevalence of Severe Food Insecurity and Malnutrition among HIV-Infected Adults in Senegal, West Africa. PloS One. 2015;10(11):e0141819.

66. Benzekri NA, Sambou JF, Diaw B, Sall EHI, Sall F, Niang A, et al. The dimensions of food insecurity and malnutrition among people living with HIV in Senegal, West Africa. AIDS Care. 2017 Dec;29(12):1510–6.

67. Fernandez E, D’Avanzo B, Negri E, Franceschi S, La Vecchia C. Diet diversity and the risk of colorectal cancer in northern Italy. Cancer Epidemiol Biomark Prev Publ Am Assoc Cancer Res Cosponsored Am Soc Prev Oncol. 1996 Jun;5(6):433–6.

68. La Vecchia C, Muñoz SE, Braga C, Fernandez E, Decarli A. Diet diversity and gastric cancer. Int J Cancer. 1997 Jul 17;72(2):255–7.

69. Levi F, Pasche C, La Vecchia C, Lucchini F, Franceschi S, Monnier P. Food groups and risk of oral and pharyngeal cancer. Int J Cancer. 1998 Aug 31;77(5):705–9.

70. Garavello W, Giordano L, Bosetti C, Talamini R, Negri E, Tavani A, et al. Diet diversity and the risk of oral and pharyngeal cancer. Eur J Nutr. 2008 Aug;47(5):280–4.

71. Lucenteforte E, Garavello W, Bosetti C, Talamini R, Zambon P, Franceschi S, et al. Diet diversity and the risk of squamous cell esophageal cancer. Int J Cancer. 2008 Nov 15;123(10):2397–400.

72. Isa F, Xie L-P, Hu Z, Zhong Z, Hemelt M, Reulen RC, et al. Dietary consumption and diet diversity and risk of developing bladder cancer: results from the South and East China case-control study. Cancer Causes Control CCC. 2013 May;24(5):885–95.

73. McCann SE, Randall E, Marshall JR, Graham S, Zielezny M, Freudenheim JL. Diet diversity and risk of colon cancer in western New York. Nutr Cancer. 1994;21(2):133–41.

74. Fernandez E, Negri E, La Vecchia C, Franceschi S. Diet diversity and colorectal cancer. Prev Med. 2000 Jul;31(1):11–4.

75. Garavello W, Lucenteforte E, Bosetti C, Talamini R, Levi F, Tavani A, et al. Diet diversity and the risk of laryngeal cancer: a case-control study from Italy and Switzerland. Oral Oncol. 2009 Jan;45(1):85–9.

76. Mohamadpour M, Sharif ZM, Keysami MA. Food insecurity, health and nutritional status among sample of palm-plantation households in Malaysia. J Health Popul Nutr. 2012 Sep;30(3):291–302.

77. de Oliveira EP, McLellan KCP, Vaz de Arruda Silveira L, Burini RC. Dietary factors associated with metabolic syndrome in Brazilian adults. Nutr J. 2012 Mar 14;11:13.

78. Gholizadeh F, Moludi J, Lotfi Yagin N, Alizadeh M, Mostafa Nachvak S, Abdollahzad H, et al. The relation of Dietary diversity score and food insecurity to metabolic syndrome features and glucose level among pre-diabetes subjects. Prim Care Diabetes. 2018 Apr 9;

79. Tian X, Xu X, Zhang K, Wang H. Gender difference of metabolic syndrome and its association with dietary diversity at different ages. Oncotarget. 2017 Sep 26;8(43):73568–78.

80. Conklin AI, Monsivais P, Khaw K-T, Wareham NJ, Forouhi NG. Dietary Diversity, Diet Cost, and Incidence of Type 2 Diabetes in the United Kingdom: A Prospective Cohort Study. PLoS Med. 2016 Jul;13(7):e1002085.

81. Wahlqvist ML, Lo CS, Myers KA. Food variety is associated with less macrovascular disease in those with type II diabetes and their healthy controls. J Am Coll Nutr. 1989 Dec;8(6):515–23.

82. Mayega RW, Guwatudde D, Makumbi F, Nakwagala FN, Peterson S, Tomson G, et al. Diabetes and pre-diabetes among persons aged 35 to 60 years in eastern Uganda: prevalence and associated factors. PloS One. 2013;8(8):e72554.

83. Danquah I, Galbete C, Meeks K, Nicolaou M, Klipstein-Grobusch K, Addo J, et al. Food variety, dietary diversity, and type 2 diabetes in a multi-center cross-sectional study among Ghanaian migrants in Europe and their compatriots in Ghana: the RODAM study. Eur J Nutr. 2017 Sep 25;

84. Azadbakht L, Mohammadifard N, Akhavanzanjani M, Taheri M, Golshahi J, Haghighatdoost F. The association between dietary glycemic index, glycemic load and diet quality indices in Iranian adults: results from Isfahan Healthy Heart Program. Int J Food Sci Nutr. 2016;67(2):161–9.

85. Masset G, Scarborough P, Rayner M, Mishra G, Brunner EJ. Can nutrient profiling help to identify foods which diet variety should be encouraged? Results from the Whitehall II cohort. Br J Nutr. 2015 Jun 14;113(11):1800–9.

86. Gicevic S, Gaskins AJ, Fung TT, Rosner B, Tobias DK, Isanaka S, et al. Evaluating pre-pregnancy dietary diversity vs. dietary quality scores as predictors of gestational diabetes and hypertensive disorders of pregnancy. PloS One. 2018;13(4):e0195103.

87. Miller WL, Crabtree BF, Evans DK. Exploratory study of the relationship between hypertension and diet diversity among Saba Islanders. Public Health Rep Wash DC 1974. 1992 Aug;107(4):426–32.

88. Oliveira EP de, Camargo KF de, Castanho GKF, Nicola M, Portero-McLellan KC, Burini RC. Dietary variety is a protective factor for elevated systolic blood pressure. Arq Bras Cardiol. 2012 Apr;98(4):338–43.

89. Mwanri AW, Kinabo JL, Ramaiya K, Feskens EJM. High blood pressure and associated risk factors among women attending antenatal clinics in Tanzania. J Hypertens. 2015 May;33(5):940–7.

90. Dzien A, Winner H, Theurl E, Dzien-Bischinger C, Lechleitner M. Food intake patterns and cardiovascular disease in different age cohorts: The relevance of food variety. E-SPEN Eur E-J Clin Nutr Metab. 2011 Jun 1;6(3):e116–20.

91. Fung TT, Isanaka S, Hu FB, Willett WC. International food group-based diet quality and risk of coronary heart disease in men and women. Am J Clin Nutr. 2018 Jan 1;107(1):120–9.

92. Hashemi Kani A, Alavian SM, Esmaillzadeh A, Adibi P, Azadbakht L. Dietary Quality Indices and Biochemical Parameters Among Patients With Non Alcoholic Fatty Liver Disease (NAFLD). Hepat Mon. 2013;13(7):e10943.

93. Zerfu TA, Umeta M, Baye K. Dietary diversity during pregnancy is associated with reduced risk of maternal anemia, preterm delivery, and low birth weight in a prospective cohort study in rural Ethiopia. Am J Clin Nutr. 2016 Jun;103(6):1482–8.

94. Saaka M, Rauf AA. Role of Dietary Diversity in Ensuring Adequate Haematological Status During Pregnancy. Int J Med Res Health Sci. 2015;4(4):749–55.

95. Abriha A, Yesuf ME, Wassie MM. Prevalence and associated factors of anemia among pregnant women of Mekelle town: a cross sectional study. BMC Res Notes. 2014 Dec 9;7:888.

96. Abay A, Yalew HW, Tariku A, Gebeye E. Determinants of prenatal anemia in Ethiopia. Arch Public Health Arch Belg Sante Publique. 2017;75:51.

97. Gebremedhin S, Enquselassie F. Correlates of anemia among women of reproductive age in Ethiopia: Evidence from Ethiopian DHS 2005. Ethiop J Health Dev. 2011;25(1):22–30.

98. Lebso M, Anato A, Loha E. Prevalence of anemia and associated factors among pregnant women in Southern Ethiopia: A community based cross-sectional study. PloS One. 2017;12(12):e0188783.

99. Roba KT, O’Connor TP, Belachew T, O’Brien NM. Seasonal variation in nutritional status and anemia among lactating mothers in two agro-ecological zones of rural Ethiopia: A longitudinal study. Nutrition. 2015 Oct;31(10):1213–8.

100. Alaofè H, Burney J, Naylor R, Taren D. Prevalence of anaemia, deficiencies of iron and vitamin A and their determinants in rural women and young children: a cross-sectional study in Kalalé district of northern Benin. Public Health Nutr. 2017 May;20(7):1203–13.

101. Korkalo L, Erkkola M, Heinonen AE, Freese R, Selvester K, Mutanen M. Associations of dietary diversity scores and micronutrient status in adolescent Mozambican girls. Eur J Nutr. 2017 Apr;56(3):1179–89.

102. Rawat R, McCoy SI, Kadiyala S. Poor diet quality is associated with low CD4 count and anemia and predicts mortality among antiretroviral therapy-naive HIV-positive adults in Uganda. J Acquir Immune Defic Syndr 1999. 2013 Feb 1;62(2):246–53.

103. Saaka M, Oladele J, Larbi A, Hoeschle-Zeledon I. Dietary Diversity Is Not Associated with Haematological Status of Pregnant Women Resident in Rural Areas of Northern Ghana. J Nutr Metab. 2017;2017:8497892.

104. Rodríguez-Rodríguez E, Ortega RM, Andrés Carvajales P, González-Rodríguez LG. Relationship between 24 h urinary potassium and diet quality in the adult Spanish population. Public Health Nutr. 2015 Apr;18(5):850–9.

105. Gebremedhin S, Enquselassie F, Umeta M. Prevalence of prenatal zinc deficiency and its association with socio-demographic, dietary and health care related factors in rural Sidama, Southern Ethiopia: a cross-sectional study. BMC Public Health. 2011;11:898.

106. Fujita M, Lo Y-J, Baranski JR. Dietary diversity score is a useful indicator of vitamin A status of adult women in Northern Kenya. Am J Hum Biol Off J Hum Biol Counc. 2012 Dec;24(6):829–34.

107. Tangney CC, Young JA, Murtaugh MA, Cobleigh MA, Oleske DM. Self-reported dietary habits, overall dietary quality and symptomatology of breast cancer survivors: a cross-sectional examination. Breast Cancer Res Treat. 2002 Jan;71(2):113–23.

108. Poorrezaeian M, Siassi F, Qorbani M, Karimi J, Koohdani F, Asayesh H, et al. Association of dietary diversity score with anxiety in women. Psychiatry Res. 2015 Dec 15;230(2):622–7.

109. Perez-Cornago A, Zulet MA, Martinez JA. Association between mood and diet quality in subjects with metabolic syndrome participating in a behavioural weight-loss programme: a cross-sectional assessment. Nutr Neurosci. 2015 Apr;18(3):137–44.

110. Milte CM, Thorpe MG, Crawford D, Ball K, McNaughton SA. Associations of diet quality with health-related quality of life in older Australian men and women. Exp Gerontol. 2015 Apr 1;64:8–16.

111. Poorrezaeian M, Siassi F, Milajerdi A, Qorbani M, Karimi J, Sohrabi-Kabi R, et al. Depression is related to dietary diversity score in women: a cross-sectional study from a developing country. Ann Gen Psychiatry. 2017;16:39.

112. Pomer A, Buffa G, Taleo F, Sizemore JH, Tokon A, Taleo G, et al. Relationships between psychosocial distress and diet during pregnancy and infant birthweight in a lower-middle income country: “Healthy mothers, healthy communities” study in Vanuatu. Ann Hum Biol. 2018 Apr 1;1–28.

113. Chen RC-Y, Chang Y-H, Lee M-S, Wahlqvist ML. Dietary quality may enhance survival related to cognitive impairment in Taiwanese elderly. Food Nutr Res. 2011;55.

114. Tomioka K, Okamoto N, Kurumatani N, Hosoi H. Association of Psychosocial Conditions, Oral Health, and Dietary Variety with Intellectual Activity in Older Community-Dwelling Japanese Adults. PloS One. 2015;10(9):e0137656.

115. Yin Z, Fei Z, Qiu C, Brasher MS, Kraus VB, Zhao W, et al. Dietary diversity and cognitive function among elderly people: A population-based study. J Nutr Health Aging. 2017 Dec;21(10):1089–94.

116. Schebendach JE, Mayer LE, Devlin MJ, Attia E, Contento IR, Wolf RL, et al. Dietary energy density and diet variety as predictors of outcome in anorexia nervosa. Am J Clin Nutr. 2008 Apr;87(4):810–6.

117. Schebendach JE, Mayer LE, Devlin MJ, Attia E, Contento IR, Wolf RL, et al. Food choice and diet variety in weight-restored patients with anorexia nervosa. J Am Diet Assoc. 2011 May;111(5):732–6.

118. Schebendach J, Mayer LES, Devlin MJ, Attia E, Walsh BT. Dietary energy density and diet variety as risk factors for relapse in anorexia nervosa: a replication. Int J Eat Disord. 2012 Jan;45(1):79–84.

119. Manerkar K, Gokhale D. Effect of Maternal Diet Diversity and Physical Activity on Neonatal Birth Weight: A Study from Urban Slums of Mumbai. J Clin Diagn Res. 2017 Oct;11(10):YC7–11.

120. Yokoyama Y, Nishi M, Murayama H, Amano H, Taniguchi Y, Nofuji Y, et al. Dietary Variety and Decline in Lean Mass and Physical Performance in Community-Dwelling Older Japanese: A 4-year Follow-Up Study. J Nutr Health Aging. 2017;21(1):11–6.

121. Momoki C, Habu D, Ogura J, Tada A, Hasei A, Sakurai K, et al. Relationships between sarcopenia and household status and locomotive syndrome in a community-dwelling elderly women in Japan. Geriatr Gerontol Int. 2017 Jan;17(1):54–60.

122. Wagenaar I, van Muiden L, Alam K, Bowers R, Hossain MA, Kispotta K, et al. Diet-related risk factors for leprosy: a case-control study. PLoS Negl Trop Dis. 2015 May;9(5):e0003766.

123. Oktaria S, Hurif NS, Naim W, Thio HB, Nijsten TEC, Richardus JH. Dietary diversity and poverty as risk factors for leprosy in Indonesia: A case-control study. PLoS Negl Trop Dis. 2018 Mar;12(3):e0006317.

124. Whittle CR, Woodside JV, Cardwell CR, McCourt HJ, Young IS, Murray LJ, et al. Dietary patterns and bone mineral status in young adults: the Northern Ireland Young Hearts Project. Br J Nutr. 2012 Oct 28;108(8):1494–504.

125. Ghadirian P, Narod S, Fafard E, Costa M, Robidoux A, Nkondjock A. Breast cancer risk in relation to the joint effect of BRCA mutations and diet diversity. Breast Cancer Res Treat. 2009 Sep;117(2):417–22.

126. Abebe H, Abebe Y, Loha E, Stoecker BJ. Consumption of vitamin A rich foods and dark adaptation threshold of pregnant women at Damot Sore District, Wolayita, Southern Ethiopia. Ethiop J Health Sci. 2014 Jul;24(3):219–26.

127. Motokawa K, Watanabe Y, Edahiro A, Shirobe M, Murakami M, Kera T, et al. Frailty Severity and Dietary Variety in Japanese Older Persons: A Cross-Sectional Study. J Nutr Health Aging. 2018;22(3):451–6.

128. Saito E, Ueki S, Yasuda N, Yamazaki S, Yasumura S. Risk factors of functional disability among community-dwelling elderly people by household in Japan: a prospective cohort study. BMC Geriatr. 2014 Aug 26;14:93.

129. De Meyer T, Bekaert S, De Buyzere ML, De Bacquer DD, Langlois MR, Shivappa N, et al. Leukocyte telomere length and diet in the apparently healthy, middle-aged Asklepios population. Sci Rep. 2018 Apr 25;8(1):6540.

130. Erickson BA, Vaughan-Sarrazin M, Liu X, Breyer BN, Kreder KJ, Cram P. Lower urinary tract symptoms and diet quality: findings from the 2000-2001 National Health and Nutrition Examination Survey. Urology. 2012 Jun;79(6):1262–7.

131. Lim HJ, Choue R. Nutritional status assessed by the Patient-Generated Subjective Global Assessment (PG-SGA) is associated with qualities of diet and life in Korean cerebral infarction patients. Nutr Burbank Los Angel Cty Calif. 2010 Aug;26(7–8):766–71.

132. Shiraseb F, Siassi F, Qorbani M, Sotoudeh G, Rostami R, Narmaki E, et al. Higher dietary diversity is related to better visual and auditory sustained attention. Br J Nutr. 2016 Apr 28;115(8):1470–80.

133. Kant A, Schatzkin A, Harris T, Ziegler R, Block G. Dietary Diversity and Subsequent Mortality in the 1st National-Health and Nutrition Examination Survey Epidemiologic Follow-up-Study. Am J Clin Nutr. 1993 Mar;57(3):434–40.

134. Kant AK, Schatzkin A, Ziegler RG. Dietary diversity and subsequent cause-specific mortality in the NHANES I epidemiologic follow-up study. J Am Coll Nutr. 1995 Jun;14(3):233–8.

135. Schuette LK, Song WO, Hoerr SL. Quantitative Use of the Food Guide Pyramid to Evaluate Dietary Intake of College Students. J Am Diet Assoc. 1996 May;96(5):453–7.

136. Song WO, Schuette LK, Huang Y-L, Hoerr S. Food group intake patterns in relation to nutritional adequacy of young adults. Nutr Res. 1996 Sep;16(9):1507–19.

137. Drewnowski A, Henderson SA, Driscoll A, Rolls BJ. The Dietary Variety Score: assessing diet quality in healthy young and older adults. J Am Diet Assoc. 1997 Mar;97(3):266–71.

138. Kant AK, Graubard BI. Variability in selected indexes of overall diet quality. Int J Vitam Nutr Res Int Z Vitam- Ernahrungsforschung J Int Vitaminol Nutr. 1999 Nov;69(6):419–27.

139. Löwik MR, Hulshof KF, Brussaard JH. Food-based dietary guidelines: some assumptions tested for The Netherlands. Br J Nutr. 1999 Apr;81 Suppl 2:S143-149.

140. Ogle BM, Hung PH, Tuyet HT. Significance of wild vegetables in micronutrient intakes of women in Vietnam: an analysis of food variety. Asia Pac J Clin Nutr. 2001;10(1):21–30.

141. Torheim LE, Barikmo I, Parr CL, Hatløy A, Ouattara F, Oshaug A. Validation of food variety as an indicator of diet quality assessed with a food frequency questionnaire for Western Mali. Eur J Clin Nutr. 2003 Oct;57(10):1283–91.

142. Foote JA, Murphy SP, Wilkens LR, Basiotis PP, Carlson A. Dietary variety increases the probability of nutrient adequacy among adults. J Nutr. 2004 Jul;134(7):1779–85.

143. Thiele S, Mensink GBM, Beitz R. Determinants of diet quality. Public Health Nutr. 2004 Feb;7(1):29–37.

144. Torheim LE, Ouattara F, Diarra MM, Thiam FD, Barikmo I, Hatløy A, et al. Nutrient adequacy and dietary diversity in rural Mali: association and determinants. Eur J Clin Nutr. 2004 Apr;58(4):594–604.

145. Azadbakht L, Mirmiran P, Azizi F. Variety scores of food groups contribute to the specific nutrient adequacy in Tehranian men. Eur J Clin Nutr. 2005 Nov;59(11):1233–40.

146. Mirmiran P, Azadbakht L, Azizi F. Dietary diversity within food groups: an indicator of specific nutrient adequacy in Tehranian women. J Am Coll Nutr. 2006 Aug;25(4):354–61.

147. Murphy SP, Foote JA, Wilkens LR, Basiotis PP, Carlson A, White KKL, et al. Simple Measures of Dietary Variety Are Associated with Improved Dietary Quality. J Am Diet Assoc. 2006 Mar;106(3):425–9.

148. Oldewage-Theron WH, Kruger R. Food variety and dietary diversity as indicators of the dietary adequacy and health status of an elderly population in Sharpeville, South Africa. J Nutr Elder. 2008;27(1–2):101–33.

149. Roche ML, Creed-Kanashiro HM, Tuesta I, Kuhnlein HV. Traditional food diversity predicts dietary quality for the Awajún in the Peruvian Amazon. Public Health Nutr. 2008 May;11(5):457–65.

150. Arimond M, Wiesmann D, Becquey E, Carriquiry A, Daniels MC, Deitchler M, et al. Simple food group diversity indicators predict micronutrient adequacy of women’s diets in 5 diverse, resource-poor settings. J Nutr. 2010 Nov;140(11):2059S-69S.

151. Oldewage-Theron W, Kruger R. Dietary diversity and adequacy of women caregivers in a peri-urban informal settlement in South Africa. Nutrition. 2011 Apr;27(4):420–7.

152. Rathnayake KM, Madushani P, Silva K. Use of dietary diversity score as a proxy indicator of nutrient adequacy of rural elderly people in Sri Lanka. BMC Res Notes. 2012 Aug 29;5:469.

153. Arsenault JE, Yakes EA, Islam MM, Hossain MB, Ahmed T, Hotz C, et al. Very low adequacy of micronutrient intakes by young children and women in rural Bangladesh is primarily explained by low food intake and limited diversity. J Nutr. 2013 Feb;143(2):197–203.

154. Vadiveloo M, Dixon LB, Mijanovich T, Elbel B, Parekh N. Development and evaluation of the US Healthy Food Diversity index. Br J Nutr. 2014 Nov 14;112(9):1562–74.

155. Henjum S, Torheim LE, Thorne-Lyman AL, Chandyo R, Fawzi WW, Shrestha PS, et al. Low dietary diversity and micronutrient adequacy among lactating women in a peri-urban area of Nepal. Public Health Nutr. 2015 Dec;18(17):3201–10.

156. Bianchi CM, Egnell M, Huneau J-F, Mariotti F. Plant Protein Intake and Dietary Diversity Are Independently Associated with Nutrient Adequacy in French Adults. J Nutr. 2016 Nov;146(11):2351–60.

157. Nguyen PH, Huybregts L, Sanghvi TG, Tran LM, Frongillo EA, Menon P, et al. Dietary Diversity Predicts the Adequacy of Micronutrient Intake in Pregnant Adolescent Girls and Women in Bangladesh, but Use of the 5-Group Cutoff Poorly Identifies Individuals with Inadequate Intake. J Nutr. 2018 May;148(5):790–7.

158. Huang Y-C, Wahlqvist ML, Lee M-S. Appetite predicts mortality in free-living older adults in association with dietary diversity. A NAHSIT cohort study. Appetite. 2014 Dec;83:89–96.

159. Huang Y-C, Wahlqvist ML, Kao M-D, Wang J-L, Lee M-S. Optimal Dietary and Plasma Magnesium Statuses Depend on Dietary Quality for a Reduction in the Risk of All-Cause Mortality in Older Adults. Nutrients. 2015 Jul 13;7(7):5664–83.

160. Letois F, Mura T, Scali J, Gutierrez L-A, Féart C, Berr C. Nutrition and mortality in the elderly over 10 years of follow-up: the Three-City study. Br J Nutr. 2016 Sep;116(5):882–9.

161. Wahlqvist ML, Xiu L, Lee M-S, Chen RC-Y, Chen K-J, Li D. Dietary diversity no longer offsets the mortality risk of hyperhomocysteinaemia in older adults with diabetes: a prospective cohort study. Asia Pac J Clin Nutr. 2016;25(2):414–23.
